# A conserved C-terminal domain of TamB interacts with multiple BamA POTRA domains in *Borreliella burgdorferi*

**DOI:** 10.1371/journal.pone.0304839

**Published:** 2024-08-29

**Authors:** Kari T. Hall, Melisha R. Kenedy, David K. Johnson, P. Scott Hefty, Darrin R. Akins

**Affiliations:** 1 Department of Microbiology and Immunology, University of Oklahoma Health Sciences Center, Oklahoma City, Oklahoma, United States of America; 2 Chemical Computational Biology Core and the Molecular Graphics and Modeling Laboratory, University of Kansas, Lawrence, Kansas, United States of America; 3 Department of Molecular Biosciences and the Center for Chemical Biology of Infectious Disease, University of Kansas, Lawrence, Kansas, United States of America; Cornell University, UNITED STATES OF AMERICA

## Abstract

Lyme disease is the leading tick-borne infection in the United States, caused by the pathogenic spirochete *Borreliella burgdorferi*, formerly known as *Borrelia burgdorferi*. Diderms, or bacteria with dual-membrane ultrastructure, such as *B*. *burgdorferi*, have multiple methods of transporting and integrating outer membrane proteins (OMPs). Most integral OMPs are transported through the β-barrel assembly machine (BAM) complex. This complex consists of the channel-forming OMP BamA and accessory lipoproteins that interact with the five periplasmic, polypeptide transport-associated (POTRA) domains of BamA. Another system, the translocation and assembly module (TAM) system, has also been implicated in OMP assembly and export. The TAM system consists of two proteins, the BamA paralog TamA which has three POTRA domains and the inner membrane protein TamB. TamB is characterized by a C-terminal DUF490 domain that interacts with the POTRA domains of TamA. Interestingly, while TamB is found in almost all diderms, including *B*. *burgdorferi*, TamA is found almost exclusively in *Proteobacteria*. This strongly suggests a TamA-independent role of TamB in most diderms. We previously demonstrated that BamA interacts with TamB in *B*. *burgdorferi* and hypothesized that this is facilitated by the BamA POTRA domains interacting with the TamB DUF490 domain. In this study, we utilized protein-protein co-purification assays to empirically demonstrate that the *B*. *burgdorferi* TamB DUF490 domain interacts with BamA POTRA2 and POTRA3. We also observed that the DUF490 domain of TamB interacts with the accessory lipoprotein BamB. To examine if the BamA-TamB interaction is more ubiquitous among diderms, we examined BamA-TamB interactions in *Salmonella enterica serovar* Typhimurium (St). Interestingly, even though St encodes a TamA protein that interacts with TamB, we observed that the TamB DUF490 of St interacts with BamA in this organism. Our combined findings strongly suggest that the TamB-BamA interaction occurs independent of the TamA component of the TAM protein export system.

## Introduction

Lyme disease is the most common tick-borne infection in the U.S. and is primarily caused by the pathogenic spirochete *Borreliella burgdorferi*, which was recently reclassified from its prior name *Borrelia burgdorferi* [1–5]. *B*. *burgdorferi* is a spirochete that is maintained in nature by a complex enzootic life cycle in which the spirochete is most commonly transferred from an infected *Ixodes* tick to the mammalian host [6–11]. Like Gram-negative bacteria, *B*. *burgdorferi* is a diderm organism possessing both an inner and outer membrane (OM). The borrelial OM differs from most Gram-negative organisms, however, in that it lacks lipopolysaccharide on its surface [12]. Instead, *B*. *burgdorferi* expresses various lipoproteins that are differentially expressed during different stages of its unique lifecycle [13–26]. Furthermore, its OM contains 10-fold fewer integral OM proteins (OMPs) as compared to *E*. *coli*, although *B*. *burgdorferi* has been shown to also contain three unique glycolipids [27–33]. Our priority has been to characterize the OMP transport systems in *B*. *burgdorferi* to facilitate the targeting of these systems and their integral OMP cargo for therapeutics and vaccine development.

One essential OMP transport system that has been identified and characterized in Gram-negative bacteria as well as other diderm organisms is the beta-barrel assembly machinery (BAM) complex. This system consists of the essential channel-forming OMP BamA (initially referred to as YaeT) that contains five N-terminal periplasmic polypeptide transport-associated (POTRA) domains, that are numbered from the N-terminus POTRA1 through POTRA5. The BAM system of different diderms also includes a variable number of accessory BAM lipoproteins [34–37]. While BamA can vary greatly at the genetic level between organisms, its overall structure is highly conserved. BamA is not only found in all diderms but orthologs to BamA are also observed in the dual-membraned mitochondria and chloroplasts of eukaryotes [38–40]. BamA is essential in these systems and is responsible for integrating OMPs into the OM by translocating beta-strands of OMPs into the OM [41,42]. The POTRA domains themselves are all structurally similar in that they contain beta-strands and alpha-helices always ordered in a β-α-α-β-β configuration [43]. The POTRA domains also facilitate interaction between BamA and the various periplasmic accessory lipoproteins that are anchored by their lipid moieties into the inner leaflet of the outer membrane [44–50]. As noted, the number of BAM accessory lipoproteins can differ between various diderm bacteria. For example, *Escherichia coli* and *Salmonella enterica serovar* Typhimurium (*Salmonella* Typhimurium) encode four accessory lipoproteins termed BamB/C/D/E [45,51] while *Neisseria meningitidis* encodes only three, BamC/D/E [35]. We have shown that *B*. *burgdorferi* encodes only BamB/D [37]. These BAM accessory lipoproteins interact with the POTRA domains of BamA in the periplasm to facilitate the export of some OMPs and maintain membrane integrity [34,52–54].

Another more recently identified OMP transport system is termed the translocation and assembly module (TAM) complex. Like the BAM system, the TAM complex is necessary for proper assembly of bacterial OMPs [55]. This system consists of the outer membrane channel-forming protein TamA and the inner membrane (IM) anchored protein TamB. TamA is closely related to BamA but contains only three periplasmic POTRA domains, which are similarly numbered. TamB is anchored to the IM via its N-terminus and is characterized by the presence of a conserved, C-terminal domain of unknown function termed DUF490 [55,56]. TamB spans the periplasm and interacts with TamA through a DUF490-POTRA interaction [55]. Interestingly, TamB is found in nearly all diderm and Gram-negative bacteria, but TamA is present primarily in *Proteobacteria* [56]. Of note, TamB itself does not show any striking amino acid differences between species that encode TamA and species that do not [55]. While it is hypothesized that TamB serves as a lever to open the lateral gate of TamA [57], the presence of TamB in almost all diderm organisms lacking TamA suggests TamB also could play other roles in these organisms. Consistent with this hypothesis, we have shown in *B*. *burgdorferi*, which lacks TamA, that TamB interacts with BamA [58], indicating another role for TamB in diderm bacteria.

Although the TamB-BamA interaction has been observed in *B*. *burgdorferi* [58], this interaction is poorly understood. Given that the *E*. *coli* TamA POTRA domains interact with TamB [57], we examined whether the *B*. *burgdorferi* BamA POTRA domains could be responsible for the interaction between BamA and TamB in *B*. *burgdorferi*. Using protein-protein interaction studies, we have shown that specific BamA POTRA domains are preferentially involved in the TamB-BamA interaction. Additionally, we observed that the TamB DUF490 domain interacts with the BAM accessory lipoprotein BamB, but not BamD. Because most diderm bacteria encode both TamB and BamA, we additionally hypothesized that these findings may be applicable to a larger range of bacterial species. To examine this issue, we interrogated the TamB-BamA interaction using *Salmonella* Typhimurium (St), which revealed that the St TamB DUF490 domain also interacts with POTRA domains of BamA. The combined findings suggest that the TamB-BamA interaction is broader than previously recognized and can occur in Gram-negative organisms that encode both BamA and TamA.

## Results

### Structural modeling of the *E*. *coli* TamB DUF490 domain and the *B*. *burgdorferi* TamB DUF490 and BamA POTRA domains

**Fig 1:**
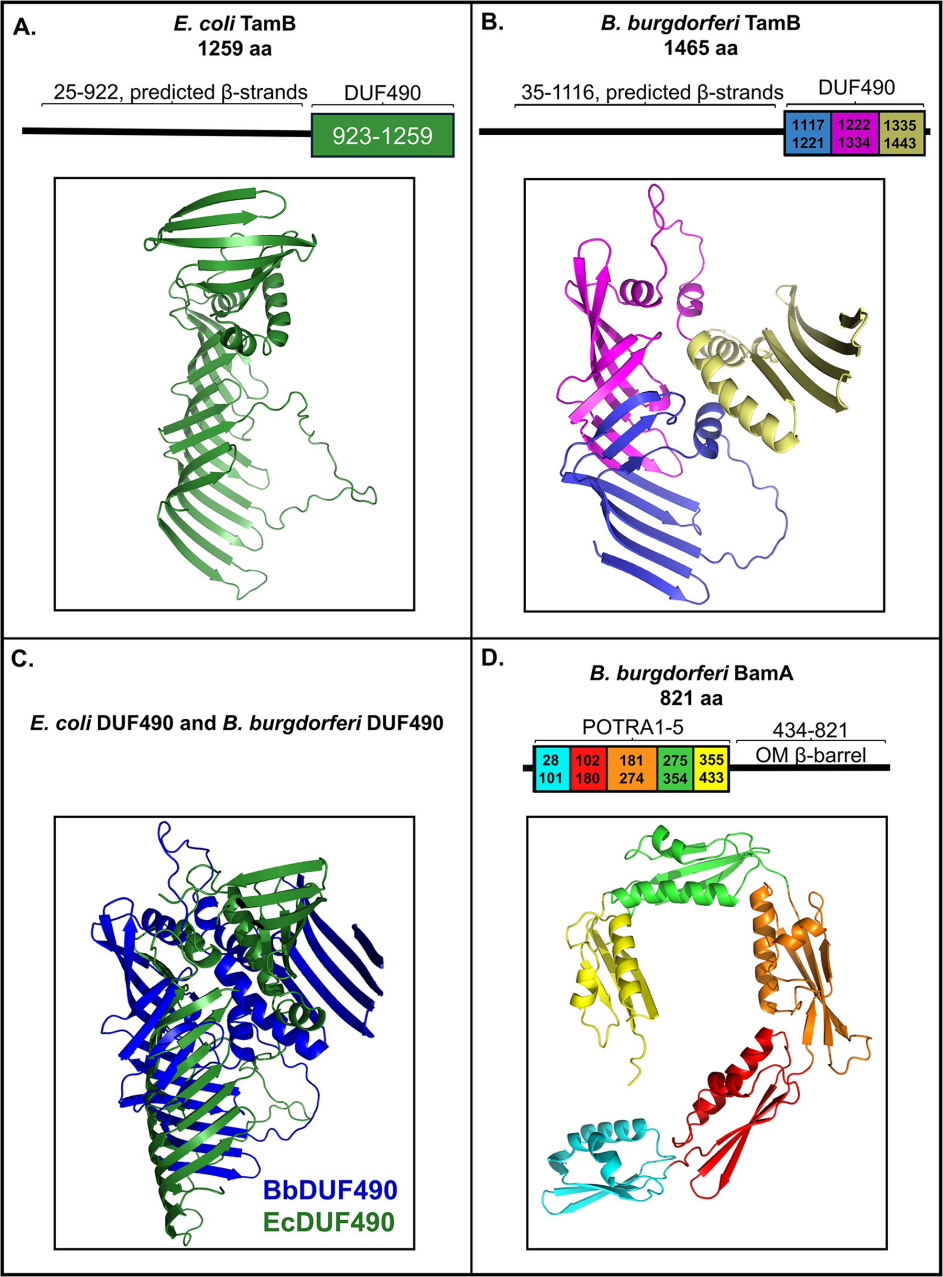
Structural models of DUF490 and POTRA domains. The colored regions under brackets were subjected to AlphaFold v2.0 modeling under default settings. All structures are oriented with the N-terminus down. **A.** AlphaFold v2.0 model of *E*. *coli* DUF490 (aa 923–1259 of TamB). **B.** AlphaFold v2.0 model of *B*. *burgdorferi* DUF490 (aa 1117–1443 of TamB) colorized to depict experimental segments dissected in this study: Segment 1 (aa 1117–1221) in blue, Segment 2 (aa 1222–1334) in magenta, and Segment 3 (aa 1335–1443) in wheat. **C.** AlphaFold v2.0 models of the DUF490 domains from *B*. *burgdorferi* (in blue) and *E*. *coli* (in green) superimposed. **D.** AlphaFold v2.0 model of *B*. *burgdorferi* BamA POTRA domains 1–5 colorized to depict individual POTRA domains: POTRA1 (aa 28–101) in cyan, POTRA2 (aa 102–180) in red, POTRA3 (aa 181–274) in orange, POTRA4 (aa 275–354) in green, and POTRA5 (aa 355–433) in yellow.

The DUF490 domain of TamB has been implicated as an important binding domain in *E*. *coli* and has been shown to interact with the POTRA domains of TamA in *Citrobacter rodentium* [55,57]. Therefore, we hypothesized that the *B*. *burgdorferi* TamB DUF490 domain might be important in the interaction we previously observed between BamA and TamB in this spirochete. Because the structure of TamB is largely undefined and predicted to be mostly beta-strands, we compared the predicted structure of the TamB-defining DUF490 domain between *B*. *burgdorferi* and *E*. *coli* utilizing AlphaFold v2.0 [59–61] and PyMOL v4.6.0 [62,63]. First, the *E*. *coli* model achieved a predicted local distant difference test (pLDDT) of 83.9, where scores closest to 100 indicate highest confidence, scores of 70–90 indicate high confidence, scores lower than 70 indicate low confidence, and scores below 50 indicate very low confidence. Of note, the AlphaFold v2.0 model in Fig 1A is similar to the only determined structure of DUF490 [64], a partial crystal structure showing the central half of *E*. *coli* DUF490 as a groove of beta-strands, known as a beta-taco.

The DUF490 of *B*. *burgdorferi* TamB (BbDUF490), consisting of the C-terminal amino acids 1117–1443, was modeled similarly and is shown in Fig 1B. The pLDDT of this model, although considered a confident score, was lower than that of the *E*. *coli* DUF490 at 73.1; this was expected, as there is no published structure for BbDUF490. The AlphaFold v2.0 model of the BbDUF490 is consistent with both the partial crystal structure of *E*. *coli* DUF490 [64] and previous secondary structure predictions for *B*. *burgdorferi* DUF490 in which its structure was predicted to consist of mostly beta-strands with some alpha-helices [58]. Interestingly, in spite of a low sequence identity of 18% and a 35% sequence similarity, the BbDUF490 model was predicted to form a beta-taco fold (Fig 1B), as was observed for the *E*. *coli* DUF490 crystal structure [64]. This can be further seen in the superimposition of the AlphaFold v2.0 models from *E*. *coli* and *B*. *burgdorferi* DUF490 models (Fig 1C) which emphasizes the beta-taco, the helical turns, and the small C-terminal beta-sheet of the domains. The beta-taco fold that makes up the majority of the structure has been hypothesized to serve as a channel for hydrophobic cargo to pass through the aqueous periplasm [64]. As described in further detail below, we sought to examine the potential interaction sites between BbDUF490 and the *B*. *burgdorferi* BamA POTRA domains. Therefore, we utilized sections of BbDUF490 to further delineate which regions of the 39 kDa DUF490 domain are important in the interaction. Modeled here, the N-terminal segment 1 forms approximately half of the beta-taco fold (Fig 1B, blue), a center segment 2 forms the second half of the beta-taco fold and a small disordered region (Fig 1B, magenta), and a C-terminal segment 3 folds back over the remainder of DUF490 with its three alpha-helices and small beta-sheet separate from the beta-taco fold (Fig 1B, wheat). Of note, when segments 1 and 2 are modeled in the absence of segment 3, the pLDDT of the AlphaFold v2.0 model increases to 81.0 while maintaining the same general structure displayed in Fig 1B (S1 Fig).

Because we aimed to examine the *B*. *burgdorferi* TamB-BamA interaction, we also modeled the POTRA domains of *B*. *burgdorferi* BamA (BbBamA). These POTRA domains are likely important in the interaction with *B*. *burgdorferi* TamB given that previous studies have shown that TamA POTRA domains interact with TamB DUF490 in other organisms [55]. The BbBamA POTRA domains are modeled in Fig 1D and obtained a pLDDT of 94.1. To better define the individual POTRA domains 1–5, the POTRAs are colorized separately despite being modeled together (Fig 1D). The five POTRA domains (BbBamAP1-5) together were predicted to be arranged in a spiral conformation that would come down from the 16-stranded beta-barrel of BamA in the OM (Fig 1D), consistent with previous crystal structures of POTRA domains from both BamA or TamA in other organisms [43,50,65–68]. Additionally, all five individual POTRA domains were predicted to have the characteristic POTRA structure of βααββ [43]. This, combined with the high pLDDT, allows a high level of confidence in the structural model of BbBamAP1-5 as a reference for the interaction studies described below.

### *In situ* modeling of the BamA-TamB interaction

**Fig 2:**
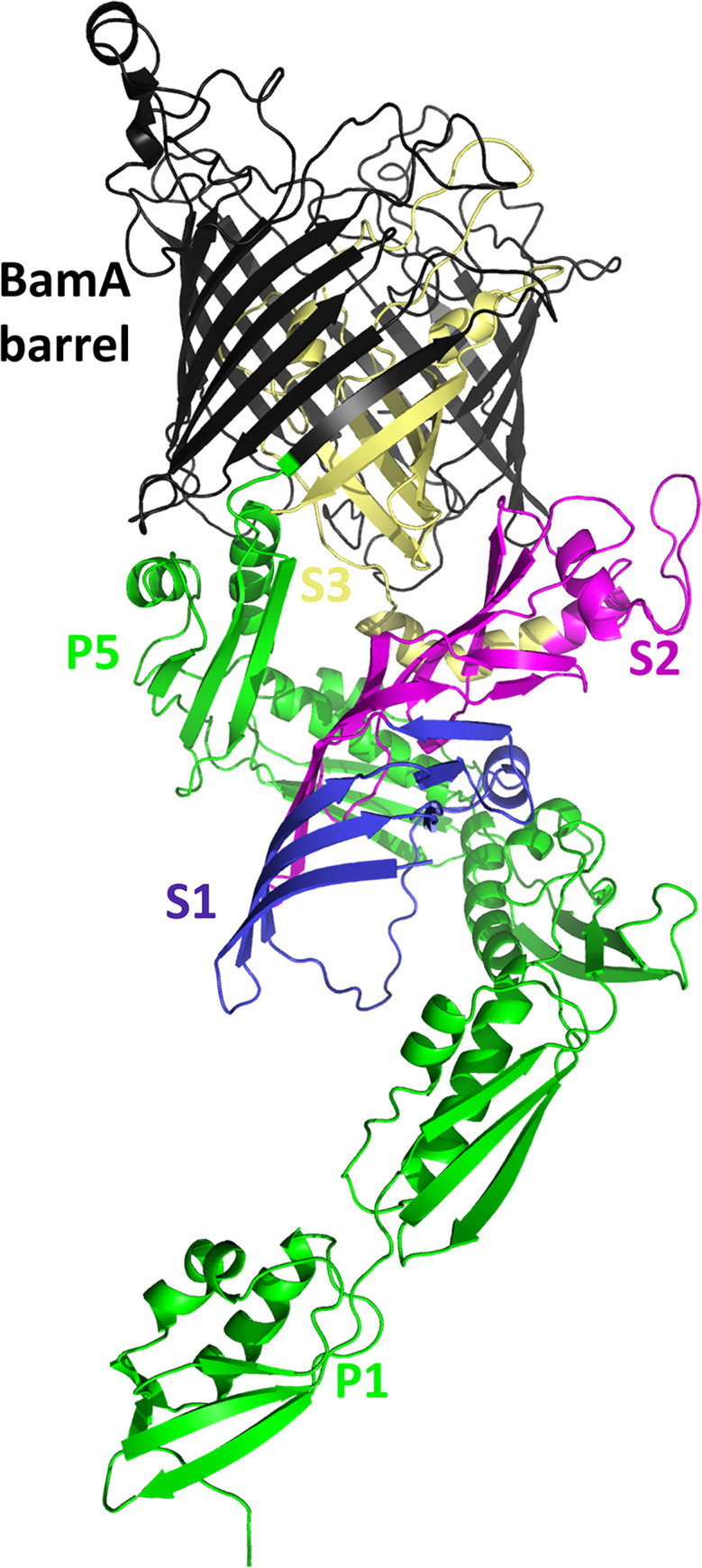
*In situ* model of *B*. *burgdorferi* TamB DUF490 interaction with *B*. *burgdorferi* BamA. The C-terminal BamA barrel is depicted in black and the N-terminal POTRA domains in green (POTRA1 labeled P1 and POTRA5 labeled P5). The DUF490 domain is depicted in three colors from the N-terminus: Segment 1 in blue (S1, aa 1117–1221), Segment 2 in magenta (S2, aa 1222–1334), and Segment 3 with the remainder of TamB (S3, aa 1335–1465) in wheat.

To investigate the hypothesis that the TamB DUF490 domain interacts with the BamA POTRA domains in *B*. *burgdorferi*, a model of the complex was attempted using AlphaFold v2.2 Multimer [69], which returns models with a predicted template modeling score (pTM) and an interface pTM (ipTM). Models are scored based on 0.8 ipTM+ 0.2 pTM, where higher values are higher confidence as with pLDDT scores. While individual chains were modeled with high confidence, with a pLDDT of 84.2 for BamA and 68.7 for segments 1 and 2 of TamB DUF490, modeled interactions generated low confidence scores with an ipTM+pTM of 0.30 and showed BamA oriented in the wrong direction. However, AlphaFold has been used to model the *E*. *coli* TamA-TamB complex [70], therefore we modeled *E*. *coli* TamB DUF490 with TamA. The resulting model was of higher confidence, with an ipTM+pTM 0f 0.59 and pLDDT values of 85.8 for TamA and 73.5 for segments 1 and 2 of TamB DUF490. This model was used as a template for homology modeling of *B*. *burgdorferi* TamB DUF490 with BamA (Fig 2) that depicts *B*. *burgdorferi* TamB DUF490 inserted between the *B*. *burgdorferi* BamA POTRA domains (green), and some contact at POTRA3 via a loop in BbDUF490 Segment 1 (Fig 2, blue). Also observed was a C-terminal portion of BbDUF490 Segment 2 (Fig 2, magenta) that is proximal to the periplasmic loops of the BbBamA barrel (Fig 2, black) and subsequently, the outer membrane, where BamA is critical for the integration of beta-barrel proteins. A region of BbDUF490 Segment 3 (Fig 2, wheat) was extended into the barrel of BbBamA. Given that the barrel of BamA is in an open conformation between β1 and β16 strands, this segment may facilitate the integration of OMPs.

### *B*. *burgdorferi* DUF490 interacts specifically with distinct *B*. *burgdorferi* BamA POTRA domains

As stated above, previous studies have shown that the TamB DUF490 domain interacts with the periplasmic POTRA domains of TamA [55,57], and the POTRA domains from BamA and TamA are structurally similar [43]. Furthermore, our structural modeling predicts that the *B*. *burgdorferi* TamB DUF490 (BbDUF490) has a similar beta-taco fold structure as described for the *E*. *coli* DUF490 domain and our initial interaction model also suggests an interaction between BbBamA POTRA3 and BbDUF490. We next examined whether a *B*. *burgdorferi* BamA POTRA-TamB DUF490 interaction could be represented using protein-protein interaction assays. To this end, we utilized a dual-expression system in which a fusion protein with a N-terminal glutathione-S-transferase (GST) tag and a fusion protein with a N-terminal 6xHis tag were co-expressed in the same *E*. *coli* strain. In these experiments, the BbDUF490 full length or the BbDUF490 segments described above (Fig 1B) served as the GST-tagged bait, while other proteins and domains served as His-tagged prey. After binding cell lysates to glutathione agarose beads, bound beads and elution fractions were prepared and immunoblots were performed with anti-GST and anti-His tag antibodies. By immunoblotting, we could confirm the purification of the GST-tagged protein and determine whether the His-tagged protein was co-purified, and thus interacting, with the GST-tagged protein. It should be noted that in these co-expression experiments, the GST tag is also present without its fusion, leading to the purification of GST on the agarose, causing a consistent band at approximately 26 kDa in both the whole cell lysate lanes and the co-purification lanes throughout the experiments.

**Fig 3:**
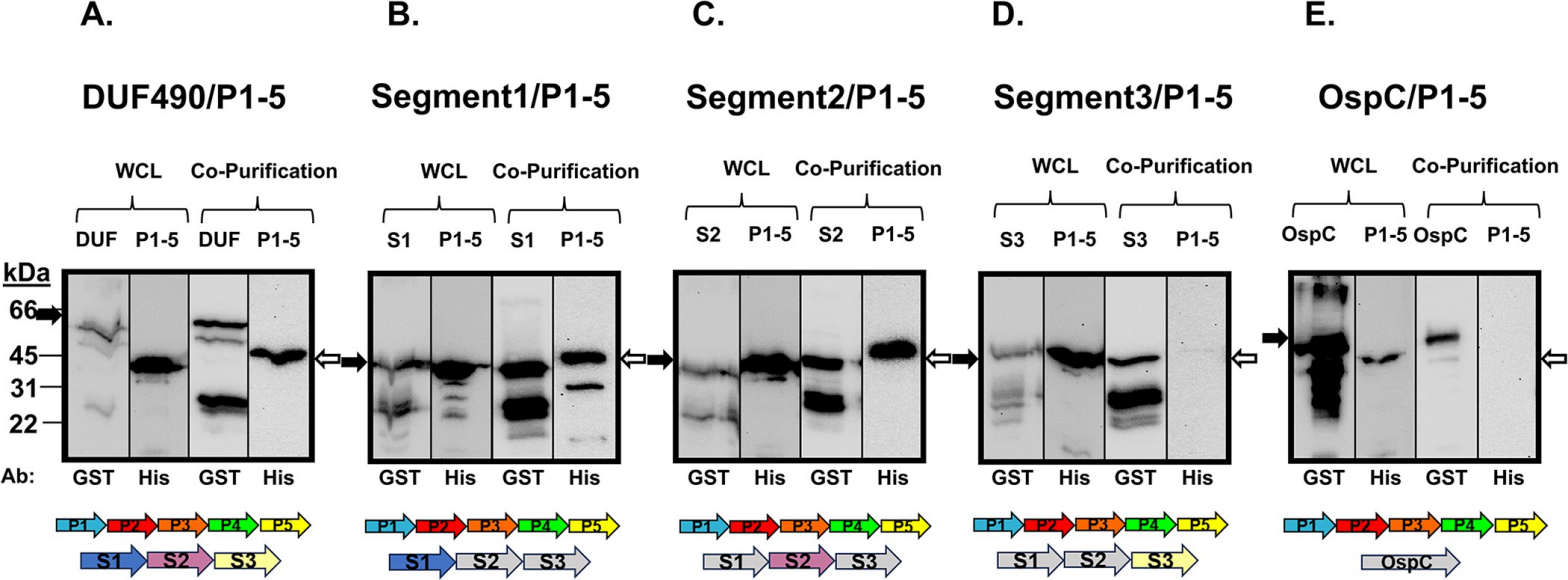
*B*. *burgdorferi* DUF490 interacts with the POTRA domains of BamA. Lower molecular weight bands (26 kDa) in anti-GST lanes correspond with the size of the GST tag. The black arrows next to blots indicate the GST fusion band and white arrows indicate the 6xHis fusion band. The colored arrows below the blots indicate proteins expressed in each assay and the expected molecular weights are listed here. **A.** Whole cell lysate and co-purification from *E*. *coli* expressing BbDUF490 (DUF, 66 kDa) as well as BbPOTRA1-5 (P1-5, 45 kDa), subjected to immunoblotting with anti-GST antibody for BbDUF490 and anti-6xHis antibody for BbPOTRA1-5. **B.** Whole cell lysate and co-purification from *E*. *coli* expressing BbDUF490 Segment 1 (S1, 39 kDa) as well as BbPOTRA1-5, subjected to immunoblotting with anti-GST antibody for Segment 1 and anti-6xHis antibody for BbPOTRA1-5. **C.** Whole cell lysate and co-purification from *E*. *coli* expressing BbDUF490 Segment 2 (S2, 39 kDa) as well as BbPOTRA1-5, subjected to immunoblotting with anti-GST antibody for Segment 2 and anti-6xHis antibody for BbPOTRA1-5. **D.** Whole cell lysate and co-purification from *E*. *coli* expressing BbDUF490 Segment 3 (S3, 39 kDa) as well as BbPOTRA1-5, subjected to immunoblotting with anti-GST antibody for Segment 3 and anti-6xHis antibody for BbPOTRA1-5. **E.** Whole cell lysate and co-purification from *E*. *coli* expressing OspC as well as BbPOTRA1-5, subjected to immunoblotting with anti-GST antibody for OspC (48 kDa) and anti-6xHis antibody for BbPOTRA1-5.

To examine the potential interaction between the *B*. *burgdorferi* DUF490 domain of TamB and the POTRA domains of the *B*. *burgdorferi* BamA, we co-expressed BbDUF490 and the *B*. *burgdorferi* POTRA 1–5 domains (BbPOTRA1-5) in the system described above and demonstrated that the proteins were both soluble in the whole cell lysate (WCL), with BbDUF490 consistently presenting as a doublet with the top band at the expected molecular weight (Fig 3A). When the GST-tagged BbDUF490 was purified, the His-tagged BbPOTRA1-5 was co-purified indicating that these domains do in fact interact (Fig 3A). We also performed these assays with the three truncated segments (Segments 1, 2, and 3) of BbDUF490. We found the interaction persisted when truncated segments of BbDUF490 were co-expressed with BbPOTRA1-5, primarily the N-terminal segment 1 (Fig 3B) and the central segment 2 (Fig 3C), but also the C-terminal segment 3 to a much lesser extent (Fig 3D). Despite the weak signal, we continued to examine potential segment 3 interactions throughout our studies due to the consistent co-purification that was detectable by immunoblot. Using GST-tagged *B*. *burgdorferi* lipoprotein OspC [71,72], the BbPOTRA1-5 did not co-purify (Fig 3E). Since OspC does not contain a BbDUF490 domain, we would not expect OspC to interact with any POTRA domains. Taken together, this data demonstrates that BbDUF490 does in fact interact specifically with the BbPOTRA domains of BamA.

**Fig 4:**
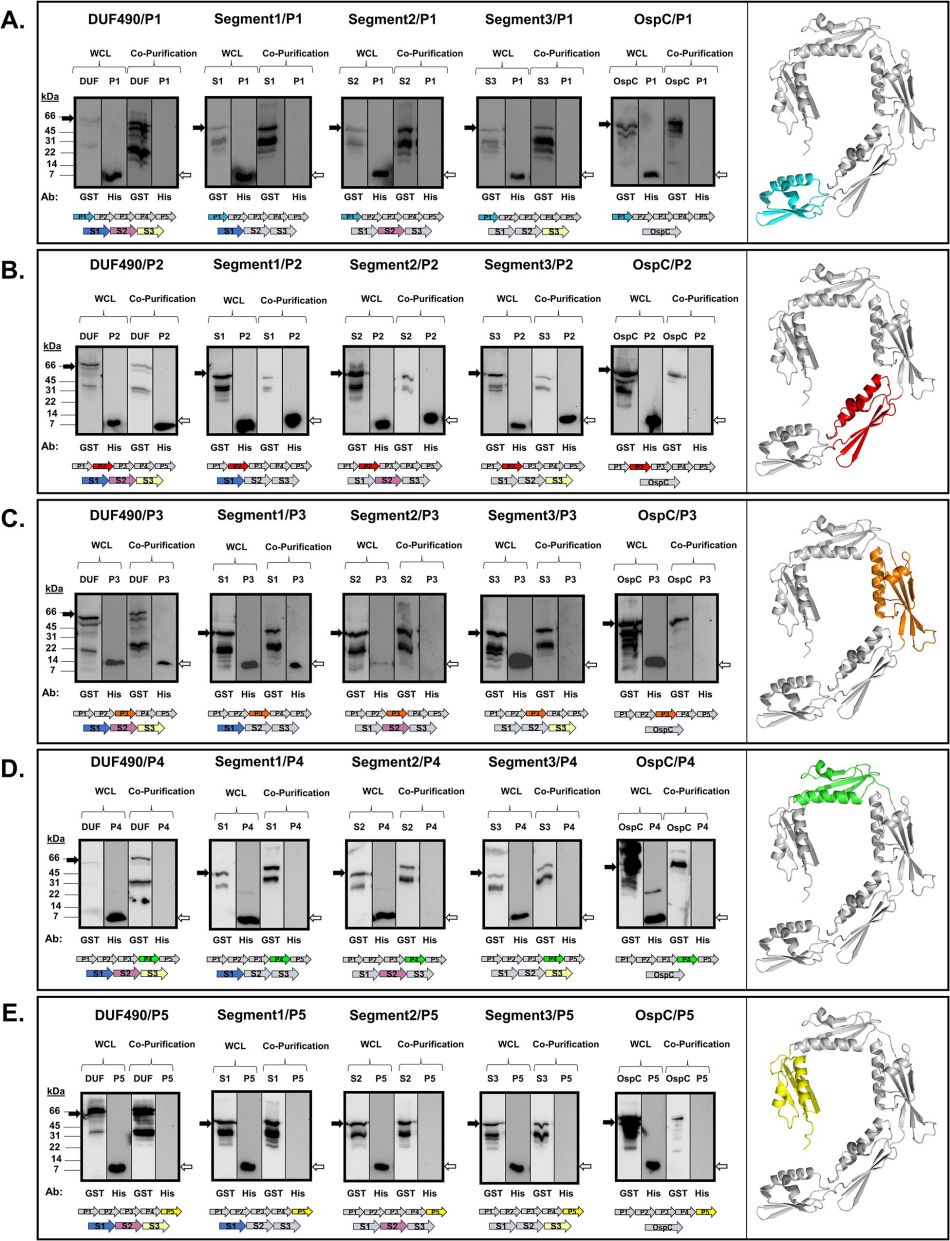
*B*. *burgdorferi* DUF490 interacts with the POTRA2 and POTRA3 domains of BamA. All samples were subjected to immunoblotting with anti-GST antibody for GST fusions and anti-6xHis antibody for BbPOTRA fusions. Lower molecular weight bands (26 kDa) in anti-GST lanes correspond with the size of the GST tag. The black arrows next to blots indicate the GST fusion band and white arrows indicate the 6xHis fusion band. The colored arrows below the blots indicate proteins co-expressed in each assay. **A.** Whole cell lysate and co-purification from *E*. *coli* expressing BbPOTRA1 (P1, 9 kDa) and BbDUF490 (66kD), BbDUF490 segments (39 kDa), or OspC (48 kDa). BbPOTRA1 is highlighted in the AlphaFold v2.0 model of BbPOTRA1-5 on the right. **B.** Whole cell lysate and co-purification from *E*. *coli* expressing BbPOTRA2 (P2, 9 kDa) and DUF490, DUF490 segments, or OspC. BbPOTRA2 is highlighted in the AlphaFold v2.0 model of BbPOTRA1-5 on the right. **C.** Whole cell lysate and co-purification from *E*. *coli* expressing BbPOTRA3 (P3, 11 kDa) and BbDUF490, BbDUF490 segments, or OspC. BbPOTRA3 is highlighted in the AlphaFold v2.0 model of BbPOTRA1-5 on the right. **D.** Whole cell lysate and co-purification from *E*. *coli* expressing BbPOTRA4 (P4, 9 kDa) and DUF490, BbDUF490 segments, or OspC. BbPOTRA4 is highlighted in the AlphaFold v2.0 model of BbPOTRA1-5 on the right. **E.** Whole cell lysate and co-purification from *E*. *coli* expressing BbPOTRA5 (P5, 9 kDa) and BbDUF490, BbDUF490 segments, or OspC. BbPOTRA5 is highlighted in the AlphaFold v2.0 model of BbPOTRA1-5 on the right.

We next examined whether the BbDUF490 full length and BbDUF490 truncations interact with specific POTRA domains. Therefore, we co-expressed the individual POTRA domains with BbDUF490 in its entirety as well as the BbDUF490 segments 1, 2, or 3 described above. For clarity, the structural model for each POTRA domain being examined is also shown and highlighted at the right of each panel. As shown in Fig 4A, we observed that BbPOTRA1 (P1) does not co-purify with BbDUF490 or any of the truncated DUF490 segments, indicating POTRA1 has no interaction with BbDUF490. In contrast, BbPOTRA2 (P2) co-purifies with the full length BbDUF490 as well as with the three BbDUF490 segments we examined, indicating specific contact points across the length of BbDUF490 (Fig 4B). Additionally, BbPOTRA3 (P3) co-purifies with the full length BbDUF490 as well as the N-terminal segment 1 truncation of BbDUF490, indicating that POTRA3 interacts specifically with the N-terminal region of the BbDUF490 domain (Fig 4C), consistent with the predicted interaction from the structural model depicted in Fig 2. It should be noted that we cannot rule out a BbPOTRA3-Segment 2 interaction due to the low expression of POTRA3 in this co-expression construct (Fig 4C). BbPOTRA4 (P4) and BbPOTRA5 (P5) did not co-purify with BbDUF490 or any segment of the BbDUF490 domain (Fig 4D and 4E). When OspC was co-expressed with any of the *B*. *burgdorferi* BamA POTRA domains, the tagged POTRA domains were not observed by immunoblot in the co-purification lanes, indicating that the interactions shown in Fig 4 were specific to the BbDUF490/BbPOTRA interactions (Fig 4A–4E). These data (also summarized in Table 1) suggest the BbDUF490 interacts with BbPOTRA2 and BbPOTRA3, but not with BbPOTRA1, BbPOTRA4, and BbPOTRA5 despite the structural similarity between all of the POTRA domains.

**Table 1:** DUF490 interacts with POTRA2 and POTRA3 in *Borreliella*.

Domain	BbDUF490	Segment 1	Segment 2	Segment 3	OspC
**BbPOTRA1**	**-**	**-**	**-**	**-**	**-**
**BbPOTRA2**	**+**	**+**	**+**	**+**	**-**
**BbPOTRA3**	**+**	**+**	**-**	**-**	**-**
**BbPOTRA4**	**-**	**-**	**-**	**-**	**-**
**BbPOTRA5**	**-**	**-**	**-**	**-**	**-**

### *B*. *burgdorferi* TamB-BamA and the BAM accessory lipoproteins

**Fig 5:**
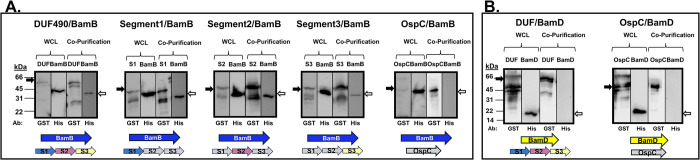
*B*. *burgdorferi* DUF490 interacts with BamB but not BamD. Lower molecular weight bands (26 kDa) in anti-GST lanes correspond with the size of the GST tag. The black arrows next to blots indicate the GST fusion band and white arrows indicate the 6xHis fusion band. Bold arrows below the immunoblot images indicate proteins co-expressed in each experiment. **A.** Whole cell lysate and co-purification from *E*. *coli* expressing BbBamB (BamB, 37 kDa) and BbDUF490 (66 kDa), BbDUF490 segments (39 kDa), or OspC (48 kDa). All samples were subjected to immunoblotting with anti-GST antibody for GST fusions and anti-6xHis antibody for BbBamB. **B.** Whole cell lysate and co-purification from *E*. *coli* expressing BbBamD (BamD, 12 kDa) and BbDUF490 (66 kDa) or OspC (48 kDa). All samples were subjected to immunoblotting with anti-GST antibody for DUF490 or OspC and anti-6xHis antibody for BbBamD.

The interaction observed in *B*. *burgdorferi* between BamA and TamB led us to investigate whether TamB might also interact with either of the borrelial BAM accessory lipoproteins BamB or BamD [37]. As previously mentioned, the BAM accessory lipoproteins are known to interact with BamA via the POTRA domains in other species [52–54], but their interaction with TamB has not been investigated. Therefore, we performed co-expression experiments with BbDUF490 and *B*. *burgdorferi* BamB (BbBamB) as well as BbDUF490 and *B*. *burgdorferi* BamD (BbBamD). As shown in Fig 5A, co-purification experiments indicate that BbDUF490 as well as any of its three segments co-purified BbBamB, indicating an interaction between the borrelial BamB and the length of BbDUF490. However, BbDUF490 did not co-purify BbBamD (Fig 5B). Again, OspC was utilized as a control for specificity and did not co-purify either BbBamB or BbBamD (Fig 5A and 5B). In conclusion, in the *B*. *burgdorferi* TAM-BAM complex, BbDUF490 interacts directly with BbBamA and BbBamB, but does not interact with BbBamD. However, we cannot rule out the possibility that a different region of TamB further interacts with the BAM lipoproteins, as our studies were limited to the BbDUF490 domain.

**Fig 6:**
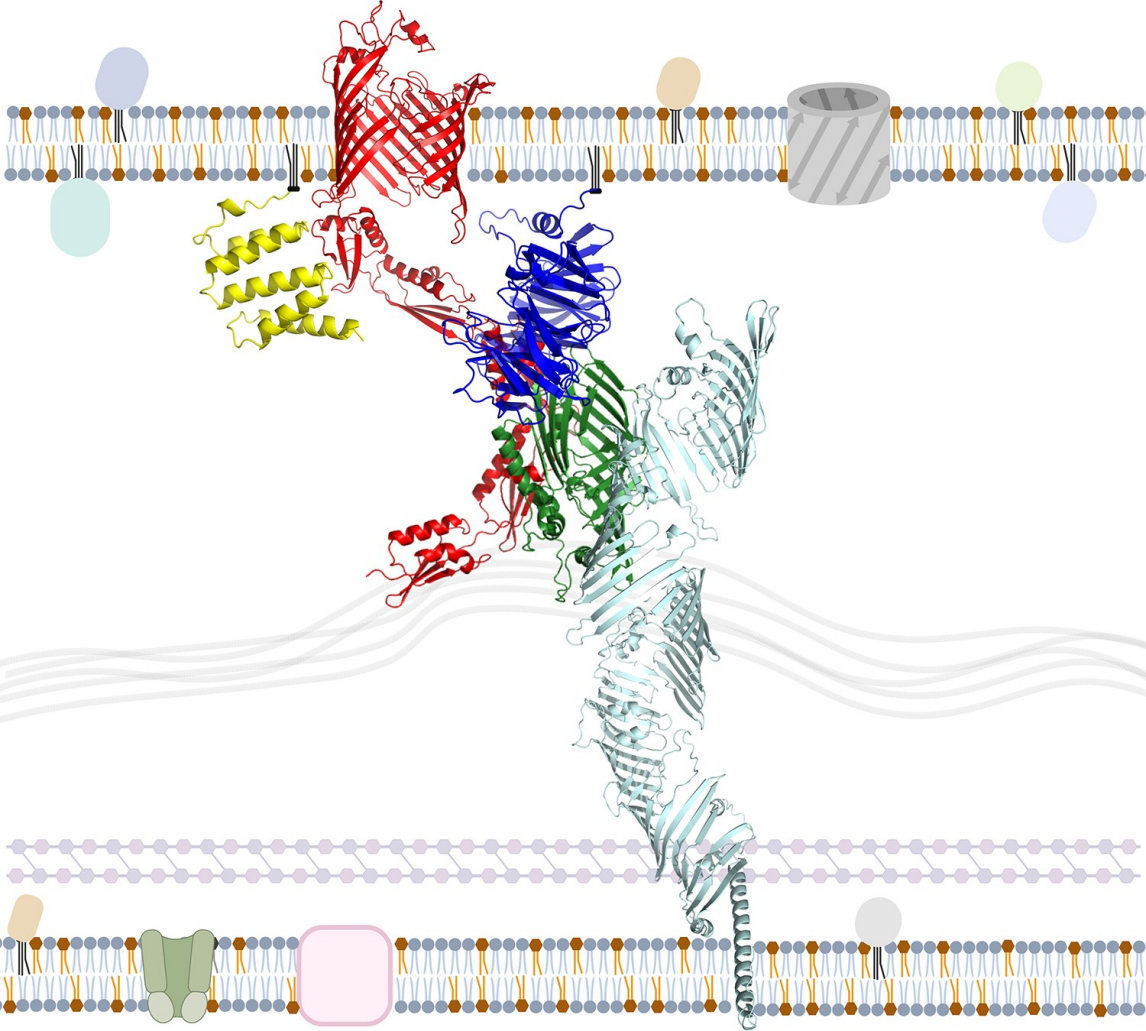
Current working model of interaction between DUF490 and the BAM complex in *Borreliella burgdorferi*. The *B*. *burgdorferi* TAM-BAM system based on combined interaction data and AlphaFold v2.0 modeling of each protein. BamA POTRA1-3 and DUF490 were modeled as a multimer and combined with the remainder of their structures. BamA is depicted in red, BamB depicted in blue, BamD depicted in yellow, TamB (aa 1–1116) depicted in mint green, and DUF490 (aa 1117–1443) depicted in dark green. The depicted membrane is a general representation of the borrelial membrane. Diacylated gray molecules represent phospholipids. Diacylated orange molecules represent glycolipids. Triacylated globular proteins represent other lipoproteins, pink squares represent metabolite transporters, green channels represent ABC transporters, and gray barrels represent porins. Created with BioRender.com.

Based on these findings and combined with the BbPOTRA-BbDUF490 data, we formed a revised working model utilizing a combination of structural models from the BAM complex and TamB. BbPOTRA1-3 and BbDUF490 were modeled using AlphaFold v2.0’s multimer model system [69] while all of the other proteins shown were modeled individually before the images were combined into one model. In the working model shown in Fig 6, we propose an interaction between BbBamB (blue) and BbDUF490 (dark green) as well as BbPOTRA2/3 and BbDUF490 whereas BbBamD (yellow) interacts with BamA POTRA domains away from the BbDUF490-BamB interaction sites.

### DUF490-POTRA domain specificity

While it is known that TamB works as part of a two-partner secretion system with TamA in other organisms [55–57], the observation that TamB directly interacts with BamA has only been described, to our knowledge, in *B*. *burgdorferi*, an organism that does not encode TamA. Therefore, we chose to investigate whether the TamB DUF490 domain might also interact specifically with BamA in organisms that encode both TamA and BamA. Here, we utilized *Salmonella enterica serovar* Typhimurium (St) as a model for these organisms given that this organism encodes both TamA and BamA, which will hereafter be referred to as StTamA and StBamA, respectively. This model selection was based on previous findings that the StTamB function in OM homeostasis was dependent on both TamA and TamB in this organism [55], that TamB is counter-selected in *Salmonella* infection models [73], and that TamA/TamB expression is at least partially controlled by the two-component virulence regulator PhoPQ [74,75]. This indicated to us that StTamB, also referred to as YtfM, is closely linked to its TamA counterpart in function and appears to play some role in infection.

**Fig 7:**
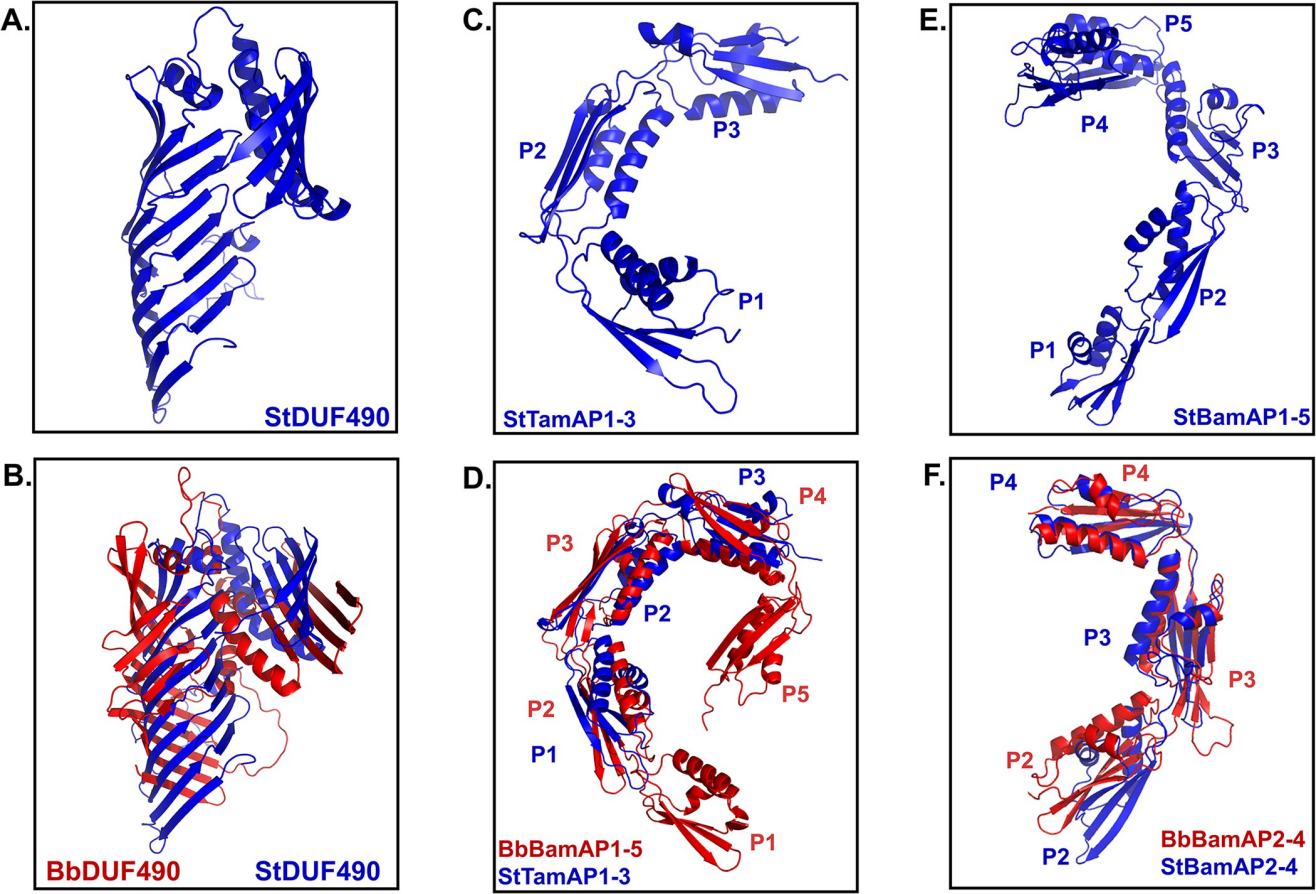
DUF490 and POTRA domains are structurally similar between species. All structures are oriented with the N-terminus down. **A.** AlphaFold v2.0 model of *S*. Typhimurium DUF490 (StDUF490). **B.** AlphaFold v2.0 model of *B*. *burgdorferi* DUF490 (BbDUF490; red) aligned with *S*. Typhimurium DUF490 (StDUF490; blue) using PyMOL. **C.** AlphaFold v2.0 model of *S*. Typhimurium TamA POTRA1-3 (StTamAP1-3). **D.** AlphaFold v2.0 model of *B*. *burgdorferi* BamA POTRA1-5 (BbBamAP1-5; red) domains aligned with *S*. Typhimurium TamA POTRA1-3 domains (StTamAP1-3; blue) using PyMOL. **E.** AlphaFold v2.0 model of *S*. Typhimurium BamA POTRA1-5 (StBamAP1-5). **F.** AlphaFold v2.0 model of *B*. *burgdorferi* BamA POTRA2-4 (BbBamAP2-4; red) aligned with *S*. Typhimurium BamA POTRA2-4 (StBamAP2-4; blue) using PyMOL.

We first modeled the structure of the StTamB DUF490 as well as the POTRA domains of StTamA and StBamA. In Fig 7, the AlphaFold v2.0 models of these domains are shown, modeled with the same parameters as the *B*. *burgdorferi* domains above. First, the St DUF490 domain (StDUF490) is predicted to have the same beta-taco fold structure as BbDUF490 as well as the same 3 alpha helices predicted between the beta-taco fold and the most C-terminal beta-strands (Fig 7A and 7B), with a pLDDT of 83.3 indicating a confident score. However, the model suggested a more pronounced bend of the C-terminal strands toward the beta-taco in the BbDUF490 model than there is in the StDUF490 model (Fig 7B). We also examined the structural similarities and differences between the three POTRA domains of StTamA (Fig 7C, pLDDT: 93.1) and five POTRA domains of StBamA (Fig 7E, pLDDT: 89.6) as compared to the *B*. *burgdorferi* POTRA domains of BbBamA. The StTamA POTRA domains have the predicted spiral architecture spanning the periplasm as was observed for the *B*. *burgdorferi* BamA POTRA domains (Fig 7C). Interestingly, the StTamA POTRA1 aligned with the BbBamA POTRA2 in PyMOL analysis of the AlphaFold v2.0 models (Fig 7D). Not surprisingly, the StBamA POTRA1-5 domains were also predicted to form a spiral configuration spanning the periplasm as was described for the other POTRA domains (Fig 7E). Because our interaction studies above indicate BbBamA POTRA2 and BbBamA POTRA3 are the sites of interaction for BbDUF490, we elected to focus specifically and compare the POTRA2-4 regions of StBamA (pLDDT: 88.1) and BbBamA (pLDDT: 95.5) (Fig 7F). We noted that the angle between StBamA POTRA2 and POTRA3 was greater than that of the angle between BbBamA POTRA2 and POTRA3, which is consistent with the prior observations that this region is flexible [76,77].

**Fig 8:**
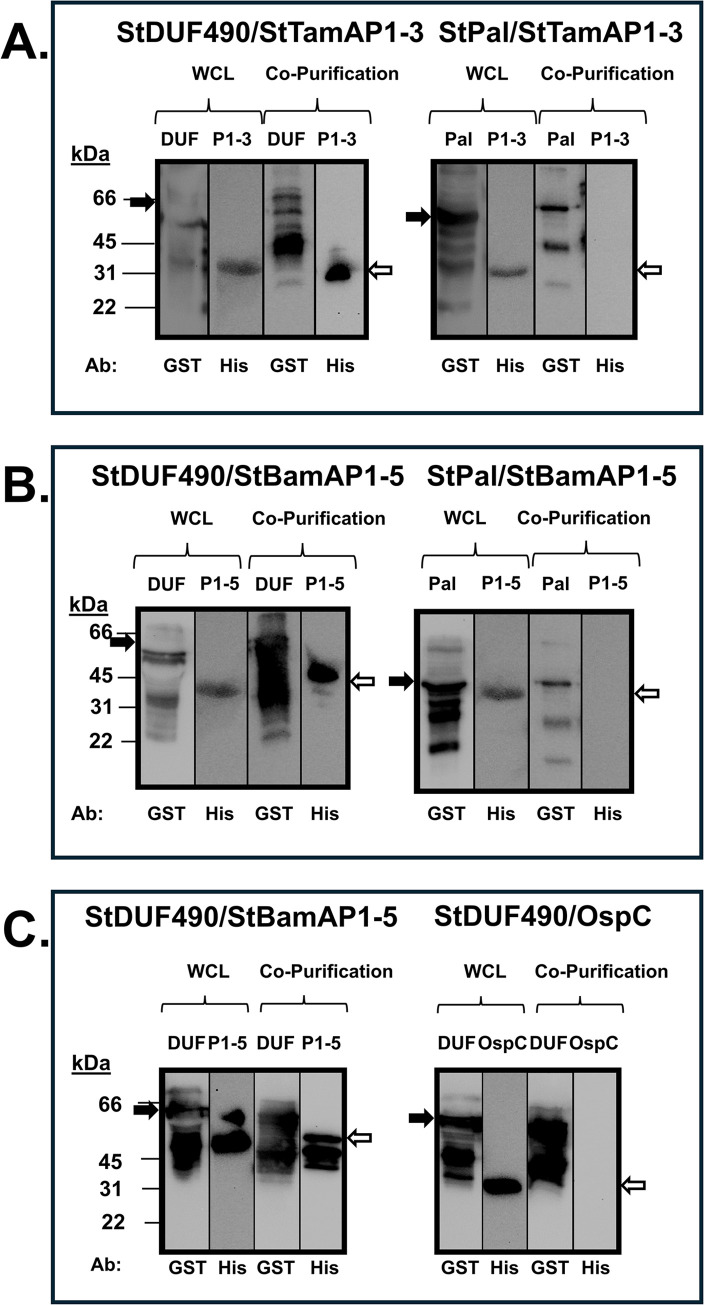
*Salmonella* DUF490 interacts with the POTRA domains of both TamA and BamA. Lower molecular weight bands (26 kDa) in anti-GST lanes correspond with the size of the GST tag. The black arrows next to blots indicate the GST fusion band and white arrows indicate the 6xHis fusion band. **A.** Whole cell lysate and co-purification from *E*. *coli* expressing one of the following: StDUF490 (DUF, 66 kDa) or Pal (42 kDa) as well as StTamA POTRA1-3 (P1-3, 31 kDa), subjected to immunoblotting with anti-GST antibody for GST fusions and anti-6xHis antibody for StTamAPOTRA1-3. **B.** Whole cell lysate and co-purification from *E*. *coli* expressing one of the following: StDUF490 (DUF, 66 kDa) or Pal (42 kDa) as well as StBamA POTRA1-5 (P1-5, 45 kDa), subjected to immunoblotting with anti-GST antibody for GST fusions and anti-6xHis antibody for StBamAPOTRA1-5. **C.** Whole cell lysate and co-purification from *E*. *coli* expressing StDUF490 (DUF, 66 kDa) and one of the following: StBamA POTRA1-5 (P1-5, 45 kDa) or OspC (22 kDa), subjected to immunoblotting with anti-GST antibody for StDUF490 and anti-6xHis antibody for StBamA POTRA1-5 and OspC.

Next, we performed similar co-purification experiments as described above to examine the potential interactions between the StDUF490 and POTRA domains from both StTamA and StBamA. As seen in Fig 8A, StDUF490 co-purifies StTamA POTRA1-3 (StTamAP1-3), which was expected given that the interaction between TamA and TamB has been established in *E*. *coli* and *C*. *rodentium* [55,57]. Interestingly, StDUF490 also co-purifies the StBamA POTRA1-5 (StBamAP1-5) domain (Fig 8B). In contrast, a GST-tagged Pal lipoprotein from St, which served as a control for specificity for these experiments, did not co-purify either of these POTRA domains (Fig 8A and 8B). As an additional control, we show that StDUF490 also did not interact non-specifically with a His-tagged OspC protein when compared to His-tagged StBamAP1-5 (Fig 8C). Importantly, the co-purifications shown here suggest that the StTamB can interact with the POTRA domains from both StTamA and StBamA, indicating that there are conserved motifs within these POTRA domains that are important for interaction with StDUF490.

**Fig 9:**
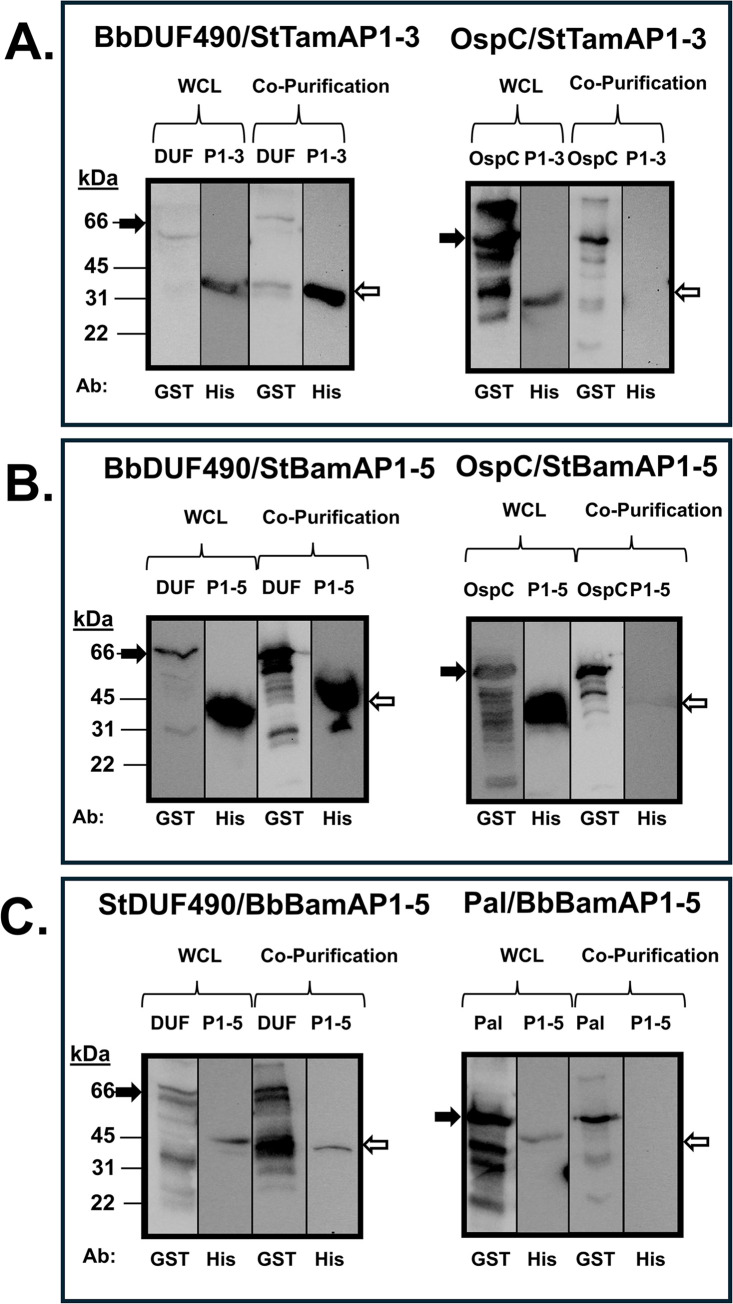
DUF490 interacts with BamA/TamA POTRA domains regardless of source species. Lower molecular weight bands (26 kDa) in anti-GST lanes correspond with the size of the GST tag. The black arrows next to blots indicate the GST fusion band and white arrows indicate the 6xHis fusion band. **A.** Whole cell lysate and co-purification from *E*. *coli* expressing one of the following: BbDUF490 (DUF, 66 kDa) or OspC (48 kDa) as well as StTamA POTRA1-3 (P1-3, 31 kDa), subjected to immunoblotting with anti-GST antibody for GST fusions and anti-6xHis antibody for StTamA POTRA1-3. **B.** Whole cell lysate and co-purification from *E*. *coli* expressing one of the following: BbDUF490 (DUF, 66 kDa) or OspC (48 kDa) as well as StBamA POTRA1-5 (P1-5, 45 kDa), subjected to immunoblotting with anti-GST antibody for GST fusions and anti-6xHis antibody for StBamA POTRA1-5. **C.** Whole cell lysate and co-purification from *E*. *coli* expressing one of the following: StDUF490 (DUF, 66 kDa) or Pal (42 kDa) as well as BbBamA POTRA1-5 (P1-5, 45 kDa), subjected to immunoblotting with anti-GST antibody for GST fusions and anti-6xHis antibody for BbBamA POTRA1-5.

To expand upon the hypothesis that DUF490 recognizes a conserved motif within POTRA domains, we sought to determine whether the BbDUF490 domain also would display promiscuity for TamA/BamA POTRA domain binding, as we observed for *Salmonella*. To this end, we first co-expressed the BbDUF490 in combination with either the StTamA POTRA1-3 or the StBamA POTRA1-5. Interestingly, the BbDUF490 co-purifies with POTRA domains from both StTamA (Fig 9A) and StBamA (Fig 9B). We also examined whether the inverse interaction would also occur and determined that StDUF490 was able to co-purify BbBamA POTRA1-5 (Fig 9C). This again suggests a conserved interaction motif within the POTRA domains, DUF490, or both, regardless of species. As in previous experiments, OspC and Pal were utilized here as controls to demonstrate that the expressed POTRA domains did not bind other proteins non-specifically (Fig 9A–9C).

**Fig 10:**
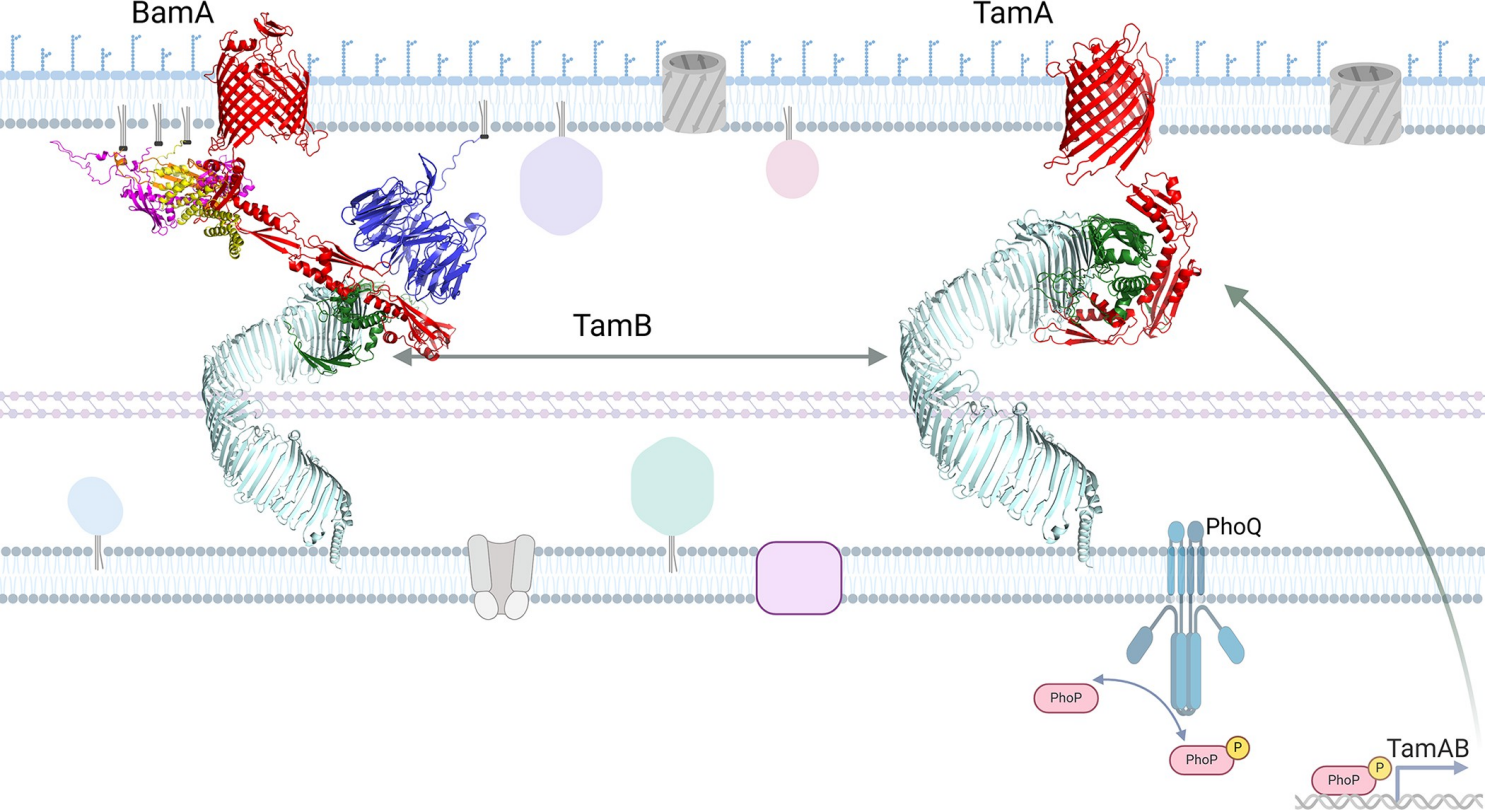
Model of *Salmonella* OMP transport systems. The *Salmonella enterica serovar* Typhimurium TAM-BAM system based on combined interaction data and AlphaFold v2.0 modeling of each protein. On the left, BamA is depicted in red, BamB in blue, BamC in magenta, BamD in yellow, BamE in orange, TamB in mint green, and DUF490 in dark green. StBamAPOTRA1-3 and StDUF490 were modeled as a multimer and combined with the remainder of their structures. On the right, TamA is depicted in red, TamB in mint green, and DUF490 in dark green. StTamAPOTRA1-3 were modeled in multimer mode with StDUF490. The depicted membrane is a general representation of a *Salmonella* membrane. Diacylated gray molecules represent phospholipids. Diacylated blue molecules represent lipopolysaccharide. Triacylated globular proteins represent other lipoproteins, pink squares represent metabolite transporters, tan channels represent ABC transporters, and gray barrels represent porins and other beta-barrel proteins. The PhoQ histidine kinase is depicted on the inner membrane and labeled. PhoP is represented as a pink rod shape, either phosphorylated (represented by the addition of a yellow circle with a P) or unphosphorylated. Created with BioRender.com.

Based on these findings, we have assembled a revised working model for TamB in St, as shown in Fig 10, where StTamB (mint green) interacts directly with StBamA (red) via StDUF490 (dark green) as illustrated on the left side. Additionally, StBamA interacts directly with StBamB (blue) and StBamD (yellow) while StBamC (magenta) and StBamE (orange) interact with StBamA indirectly via StBamD as indicated by previous studies in *E*. *coli* [52,53,78]. Interactions between BAM lipoproteins and TamB may occur in *Salmonella* but have not been investigated. As illustrated in Fig 10 on the right side, StTamB (mint green) interacts directly with StTamA (red) via StDUF490 (dark green). It has been recently demonstrated that PhoPQ directly induces expression of StTamA and StTamB, which appear to be translationally coupled [74]. As a result, transcription of TamA/B can be induced by several different environmental signals including pH, cation concentrations, and the presence of antimicrobial peptides [79–82]. Once expressed, however, TamB could then interact with either TamA and/or BamA.

## Discussion

Both the BAM and TAM complexes have an important role in transporting OMPs and maintaining OM integrity in diderm organisms [36,37,46,48,58,66,74,83–87]. Depletion of the channel-forming BamA in bacteria results in increased antibiotic sensitivity and defects in OMP transport [36,46,48,88]. The BAM complex involves interaction between BAM accessory lipoproteins and the N-terminal BamA POTRA domains for OMP export [47,49,50,67,86,88–90]. While a number of studies have sought to elucidate the role of the TAM complex [55,58,74,83,84,91,92], the majority of these studies have focused on *Proteobacteria* that encode both the TamA and TamB proteins [55,84,91–94]. In these organisms, the DUF490 domain of TamB has been shown to interact with the most N-terminal POTRA domain of TamA [55,57]. The TAM system has been shown to be associated with the secretion of virulence associated OMPs, including the adhesins Ag43 and EhaA in *E*. *coli* [55]. While studies of TamB in diderms that lack TamA are limited, they have indicated that TamB plays important roles in OM integrity, OMP transport, and cell division [58,83]. We have previously shown that there is a TamB-BamA interaction in *B*. *burgdorferi* and other studies have also suggested this interaction [56,58,95–98]. There have been no studies, however, that have examined the TamB-BamA interaction empirically or examined which POTRA domains of BamA might be involved in the interaction.

To examine this, a model of BbBamA in complex with the BbTamB DUF490 was created using a combination of AlphaFold and homology modeling. Although AlphaFold was unable to model the complex, the individual chains were modeled with moderate confidence. McDonnell et al. had successfully used AlphaFold to model *E*. *coli* TamA (EcTamA) in complex with *E*. *coli* TamB (EcTamB) [70]; therefore, we generated a model of EcTamA in complex with the EcTamB DUF490. Comparison of the individual *B*. *burgdorferi* proteins to those of *E*. *coli* suggested that, while the sequences had low similarity, they did have structural similarity, although EcTamA was in an opened state to accommodate a beta-sheet at the far C-terminus of EcTamB. Thus, we turned to homology modeling using the AlphaFold generated EcTamA-EcTamB complex and the periplasmic portions of the AlphaFold generated BbBamA and BbTamB models as templates. The final model featured the C-terminus of BbTamB inserted into the BamA beta-barrel similarly to the EcTamB with EcTamA, but only included an interaction with POTRA3. However, the interacting BbTamB sequence was selected based on the *E*. *coli* TamA-TamB model, which only contains three TamA POTRA domains rather than the five BamA POTRA domains seen in the *B*. *burgdorferi* model and lacks any intermediate interactions that would be involved with recognition or insertion of TamB into BamA.

To further examine this, we utilized protein-protein interaction assays. We demonstrated that the *B*. *burgdorferi* DUF490 domain of TamB interacts preferentially with BamA POTRA2 and POTRA3, with the POTRA3 interaction occurring specifically with the N-terminal region of DUF490. The latter interaction is consistent with that predicted from the model while the POTRA2 interaction was less expected. Interestingly, structural studies on different organisms have revealed that the region between BamA POTRA2 and POTRA3 is the most flexible portion within the BamA POTRA periplasmic region [65,77], with a more open angle represented in our *in situ* model. Additionally, a characteristic of BamA proteins is a lateral gate between the first transmembrane β-strand and the final β-strand of BamA, which serves as an opening for the integration of OMPs into the OM lipid bilayer [42,66,68]. The DUF490 domain of TamB has been proposed to serve as a lever to open the lateral gate of TamA [57] and hold the POTRA domains of BamA in an extended conformation to facilitate the opening of the BamA lateral gate [98]. Our combined data lead us to postulate that the TamB DUF490 domain interacts preferentially with BamA POTRA2 and POTRA3 to help stabilize this highly flexible POTRA region into an open angle, which in turn could potentially extend the POTRA domains and help to modulate the lateral gate of BamA.

The protein-protein interaction studies also revealed an interaction between the *B*. *burgdorferi* TamB DUF490 domain and the BAM accessory lipoprotein BamB. While BamB is known to interact with the BamA POTRA domains [49,89,99], no direct interaction between TamB and BamB has been previously described to our knowledge. BamB is generally considered to be an accessory lipoprotein that enhances overall OMP transport into the OM [48,49,54,99–102]. Its proposed functions include beta-augmentation of cargo OMPs, in which the beta-strands of OMPs are pre-oriented along BamB before transport by BamA [100], as well as spatial coordination of the BAM complex within the membrane [101]. A recent study in St has also shown severe OM defects in a TamA-TamB-BamB triple mutant over a TamA-TamB double mutant, demonstrating the potential for an important role in BamB-TAM interactions in overall membrane biogenesis [74]. These observations could suggest that in coordination with the BamA POTRA2/3-TamB DUF490 interaction in *B*. *burgdorferi* that the BamB-TamB interaction may serve to assist proper protein integration into the OM. Future mutational analyses will be required to directly examine this hypothesis.

To further analyze the BamA-TamB interaction, we also examined a species in which both TamA and TamB are present. This line of experimentation demonstrated that the DUF490 domain of TamB from St interacts with the POTRA domains of TamA and the POTRA domains of BamA indicating that a direct interaction occurs between TamB and BamA in organisms that also encode TamA. Our observation that TamB interacts with both BamA and TamA is not unprecedented [58,96,98] but leads to the intriguing notion that specific OMPs may utilize only one transport system (i.e., either the TamA-TamB system or the BamA-TamB system). Consistent with this conjecture, the TamA and BamA barrels of *E*. *coli* have structural distinctions at the lateral gate between the first and last beta-strands, possibly providing specificity for specific OMP translocation [92]. Of note, the TamAB operon has recently been shown to be regulated by the virulence regulator PhoPQ in *Salmonella* [74], suggesting a role in response to host environment and the corresponding threats to bacterial survival. While TamA and TamB seem to play a role in virulence, BamA is essential in all diderm bacteria [36,46,48]. Therefore, we propose that BamA/TamA-encoding species utilize the TAM complex as a specialty exporter for virulence and stress-related membrane responses while nearly all diderm bacteria utilize TamB in cooperation with the BAM complex for homeostatic membrane biogenesis. The mechanism by which export of specific OMPs is regulated between these transport systems is an important area for future studies.

Further analysis of the BAM and TAM complexes’ interaction sites and substrate interaction sites could serve to elucidate their specific roles. Our results clearly demonstrate that the POTRA1 domain of TamA from St aligned best with the POTRA2 domain of BamA from *B*. *burgdorferi* using PyMOL analysis of the AlphaFold v2.0 models. This was an interesting observation given that TamB interacts with the TamA POTRA1 domain in *C*. *rodentium* and *E*. *coli* [55,57]. This finding is also consistent with our observation that the *B*. *burgdorferi* BamA POTRA2 domain interacted with the TamB DUF490. These combined observations strongly suggest that there are likely conserved residues and/or structural motifs in either the POTRA domains, DUF490, or both that are important for the interactions between TamB and POTRA1 or POTRA2 in TamA or BamA, respectively. Consistent with these observations, we found that POTRA domains from one organism can interact with the DUF490 of another organism, presumably reflecting the important role of structural similarity in these interactions given the low sequence similarities between POTRA and DUF490 domains among different organisms. While the DUF490 domain shares more similarity between TamB proteins than the remainder of TamB, sequence similarity between species is frequently less than 40%. The BamA POTRA domains present a similar challenge. While this study was aimed at demonstrating the general interaction between BamA and TamB, future studies will be important to fully identify the key motifs and/or residues responsible for the DUF490/POTRA interactions identified in this study.

## Methods

### Structural modeling

The sequences of the respective domains were inputted to AlphaFold v2.0 [59–61] with default parameters. The model utilized was selected based on pLDDT score and visual appearance. For the structural superimposition between the *E*. *coli* and *B*. *burgdorferi* DUF490 models, the ‘super’ function in PyMOL v4.6.0 was utilized [62,63]. For the remaining structural comparison images, the AlphaFold v2.0 models were aligned using the ‘align’ function in PyMOL v4.6.0 [62,63]. These models are shown in Figs 1, 7 and S1.

In an attempt to determine the structural basis for the interaction between TamB and BamA in *B*. *burgdorferi*, AlphaFold v2.2 was used to attempt to model full-length BbBamA in complex with the BbTamB DUF490 (residues 901–1465), but no models were generated with a realistic interaction [60,69]. Therefore, AlphaFold was used to model full length *E*. *coli* TamA in complex with *E*. *coli* TamB DUF490 (residues 963–1259) using AlphaFold v2.2 and only generating one structure per model. A sequence alignment between *E*. *coli* TamA and TamB with the *B*. *burgdorferi* BamA and TamB (residues 1113–1465) was created by hand based on substructural alignments within the AlphaFold models. Homology modeling was performed using RosettaCM [103] with the EcTamA-EcTamB model, residues 1–407 of the BbBamA model, and residues 1113–1325 from the BbTamB model as the templates. *Borreliella* sequences were threaded onto the templates, fragment libraries were generated, and homology modeling was done using the hybridize protocol within Rosetta [104]. 10,000 models were generated, and the top 100 models were visually inspected. Five were selected for a subsequent relaxation within Rosetta, generating 1,000 structures each. The lowest energy complex was selected and is shown in Fig 2. Commands run and files used are included in supplemental materials.

For the systemic models shown in Figs 6 and 10, DUF490 was modeled as a multimer with the POTRA1-3 domains of BamA or TamA as appropriate utilizing AlphaFold v2.0 [69]. The models were selected based on pTM, ipTM, pLDDT, and visual appearance. The AlphaFold v2.0 models of the remaining POTRA domains and transmembrane barrel of BamA, or the transmembrane barrel of TamA as appropriate, were joined end-to-end with the POTRA domains in the multimer model. The upstream amino acids of TamB were also modeled separately and similarly joined end-to-end with DUF490. The accessory lipoproteins were modeled individually without their signal peptides and maneuvered into the systemic model to approximate interactions. Input sequences and statistics of the resultant models are available in Tables 2 and 3 for the *Borreliella* and *Salmonella* systemic models, respectively.

**Table 2:** Sequences and statistics for the *Borreliella* systemic model.

Protein	Accession Number	pLDDT	iPTM	tPTM
**BamB (aa 25–349)**	WP_002655996.1	78.82	-	-
**BamD (aa 14–119)**	WP_002556921.1	92.9	-	-
**BamA POTRA4-5 + barrel (aa 275–821)**	WP_010889821.1	85.42	-	-
**BamA POTRA1-3 (aa 28–274) modeled with DUF490 (aa 1117–1443)**	WP_010889821.1, WP_023003307.1	68.78	0.41	0.16
**TamB ΔDUF490 (aa 1–1116)**	WP_023003307.1	63.29	-	-

**Table 3:** Sequences and statistics for the *Salmonella* systemic model.

Protein	Accession Number	pLDDT	iPTM	tPTM
**BamB (aa 20–392)**	WP_001177074.1	91.88	-	-
**BamC (aa 25–344)**	WP_000331877.1	87.09	-	-
**BamD (aa 20–245)**	WP_000197660.1	91.53	-	-
**BamE (aa 20–112)**	WP_001203445.1	91.18	-	-
**BamA POTRA4-5 + barrel (aa 264–804)**	WP_001240935.1	93.58	-	-
**BamA P1-3 (aa 24–263) modeled with DUF490 (aa 923–1259)**	WP_001240935.1, WP_000060815.1	67.08	0.41	0.19
**TamA barrel (aa 266–577)**	WP_001120231.1	96.70	-	-
**TamA POTRA 1–3 (aa 21–265) modeled with DUF490 (aa 923–1259)**	WP_001120231.1, WP_000060815.1	72.10	0.69	0.81
**TamB ΔDUF490 (aa 1–922)**	WP_000060815.1	92.21	-	-

### Generation of expression vectors for co-purification assays

The genes of interest were amplified using their respective primers, as listed in Table 4, from either *Borreliella burgdorferi* B31 genomic DNA or lysed *Salmonella enterica serovar* Typhimurium 14028s as appropriate. For all 6xHis-tagged constructs, amplicons were inserted into the pACYCDuet-1 vector (Novagen, Madison, WI). The vector and amplicons were digested with BamHI and EcoRI to allow subsequent ligation of the amplicon into the first multiple cloning site on the pACYCDuet-1 vector. An exception to this was the StTamA POTRA1-3 for which the vector and insert were digested with BamHI and XhoI due to a secondary EcoRI digestion site within the amplicon. For insertion into the pGEX-4T-1 vector (Cytiva Life Sciences, Marlborough, MA) for GST-tagged constructs, all amplicons and the vector were digested with BamHI and EcoRI and subsequently ligated into the multiple cloning site. The pACYCDuet-1 ligation reactions were electroporated into *E*. *coli* BL21 DE3 (New England BioLabs, Ipswich, MA) while the pGEX-4T-1 ligation reactions were electroporated into *E*. *coli* DH5α cells. All plasmids were sequenced through the multiple cloning site to verify that there were no mutations during cloning and that ligation placed the amplicon appropriately in-frame with the respective tags.

**Table 4:** Primers used in this study.

Primer	Sequence (5’-3’, restriction sites in bold, stop codon underlined)	Description
**OspC ACYC BamHI F**	GAG**GGATCC**TAATAATTCAGGGAAAGATGGGAATACATC	Forward primer for OspC into pACYCDuet-1. Nucleotide: 58-.
**B31 OspC EcoRI R**	GAG**GAATTC**TTAAGGTTTTTTTGGACTTTCTGC	Reverse primer for OspC into pACYCDuet-1. Nucleotide: -630.
**DUF490 BamHI F**	GAG**GGATCC**GGCGATTTTTCAATTGAAGGAAATGC	Forward primer for BbDUF490 and Segment 1 into pGEX-4T-1. Nucleotide: 3349-.
**DUF490-1 EcoRI R**	GAG**GAATTC**TTACTTAATAATAAAATCATCTGTTTTTGTGTCAGA	Reverse primer for Segment 1 into pGEX-4T-1. Nucleotide: -3663.
**DUF490-2 BamHI F**	GAG**GGATCC**GGAGATTTGAATATTGCAAG	Forward primer for Segment 2 into pGEX-4T-1. Nucleotide: 3664-.
**DUF490-2 EcoRI R**	GAG**GAATTC**TTAACTTACTATTCCAATTGCCATTTCAGCA	Reverse primer for Segment 2 into pGEX-4T-1. Nucleotide: -4002.
**DUF490-3 BamHI F**	GAG**GGATCC**GACATTGCTCTTGATTTTTTAATTCAACC	Forward primer for Segment 3 into pGEX-4T-1. Nucleotide: 4003-.
**DUF490 EcoRI R**	GAG**GAATTC**TTAGTAATCAAACTCATAATTAACCAAAAAAAATGGAG	Reverse primer for BbDUF490 and Segment 3 into pGEX-4T-1. Nucleotide: -4329.
**P1 ACYC BamHI F**	GAG**GGATCC**TAAGGGGAAAATAATAAAGGGTATTAATTTTG	Forward primer for BbPOTRA1 and BbPOTRA1-5 into pACYCDuet-1. Nucleotide: 82-.
**P1 EcoRI R**	GAG**GAATTC**TTATTCTTTTACAATAAATGTAATAAAAAGATCCT	Reverse primer for BbPOTRA1 into pACYCDuet-1. Nucleotide: -303.
**P2 ACYC BamHI F**	GAG**GGATCC**TAAATCTTTAGTTAATTCTGTTGTTTTTTCTG	Forward primer for BbPOTRA2 into pACYCDuet-1. Nucleotide: 304-.
**P2 EcoRI R**	GAG**GAATTC**TTATCCAGCTACTATATTAAAAATAATATCAACTA	Reverse primer for BbPOTRA2 into pACYCDuet-1. Nucleotide: -540.
**P3 ACYC BamHI F**	GAG**GGATCC**TCCCAAATATGTTGTTAAGGGGAT	Forward primer for BbPOTRA3 into pACYCDuet-1. Nucleotide: 541-.
**P3 EcoRI R**	GAG**GAATTC**TTAGCCTTCTGAAAGAAAATATTTCAAAAAA	Reverse primer for BbPOTRA3 into pACYCDuet-1. Nucleotide: -822.
**P4 ACYC BamHI F**	GAG**GGATCC**TAATGTTTTTAGATTTGGAAAGCTTG	Forward primer for BbPOTRA4 into pACYCDuet-1. Nucleotide: 823-.
**P4 EcoRI R**	GAG**GAATTC**TTAATCCTTTTCTAAAATTTTAATTAACAAATCAAC	Reverse primer for BbPOTRA4 into pACYCDuet-1. Nucleotide: -1062.
**P5 ACYC BamHI F**	GAG**GGATCC**TAAAGCTCATATTGAGTCTATTACTGTTT	Forward primer for BbPOTRA5 into pACYCDuet-1. Nucleotide: 1063-
**P5 EcoRI R**	GAG**GAATTC**TTAATTACTTGTTGCTCGCTCCT	Reverse primer for BbPOTRA5 and BbPOTRA1-5 into pACYCDuet-1. Nucleotide: -1299.
**Pal BamHI F**	GAG**GGATCC**AGCAATGACGGTAGCGAAGGCGGTATGCTGAAC	Forward primer for Pal into pGEX-4T-1. Nucleotide: 85-.
**Pal EcoRI R**	GAG**GAATTC**TTAGTAAACCAGTACAGCGCGACG	Reverse primer for Pal into pGEX-4T-1. Nucleotide: -522.
**BamB ACYC BamHI F**	GAG**GGATCC**TTCAAGCGAATCCATATTTTCACAAT	Forward primer for BbBamB into pACYCDuet-1. Nucleotide: 76-.
**BamB EcoRI R**	GAG**GAATTC**TTATTCTTTAGTTAATTTTCTGTTTTCCAATTTCC	Reverse primer for BbBamB into pACYCDuet-1. Nucleotide: -1047.
**BamD ACYC BamHI F**	GAG**GGATCC**TTATACGATTAACTTAGAAAAATTAACAAAAGAAAC	Forward primer for BbBamD into pACYCDuet-1. Nucleotide: 43-.
**BamD EcoRI R**	GAG**GAATTC**TTATTTTTTATTATTTTCTATTTTATTTAATATTTTTTTAGCTAAAGG	Reverse primer for BbBamD into pACYCDuet-1. Nucleotide: -357.
**StDUF490 BamHI F**	GAG**GGATCC**ACACAACAAGGGCAAATTAACC	Forward primer for StDUF490 into pGEX-4T-1. Nucleotide: 2767-.
**StDUF490 EcoRI R**	GAG**GAATTC**TTAAAACTCAAACTGATAGAGCAAATCAAGTG	Reverse primer for StDUF490 into pGGEX-4T-1. Nucleotide: -3777.
**StP1-3 ACYC BamHI F**	GAG**GGATCC**AGCCGCAAATGTTCGTCTGAAAG	Forward primer for StTamA POTRA1-3 into pACYCDuet-1. Nucleotide: 61-.
**StP1-3 XhoI R**	GCG**CTCGAG**TTATTCGGTTCGCGGCGAT	Reverse primer for StTamA POTRA1-3 into pACYCDuet-1. Nucleotide: -795.
**StP1-5 ACYC BamHI F**	GAG**GGATCC**ATTCGTAGTGAAGGACATTCATT	Forward primer for StBamA POTRA1-5 into pACYCDuet-1. Nucleotide: 70-.
**StP1-5 EcoRI R**	GAG**GAATTC**TTAGCGCTCTTTCACCTTGTACACC	Reverse primer for StBamA POTRA1-5 into pACYCDuet-1. Nucleotide: -1063.

### Generation of dual-expression cell strains

To generate dual-expressing strains that could be utilized for co-purification assays, the *E*. *coli* BL21 DE3 cells with validated pACYCDuet-1 constructs were prepared and electroporated with pGEX-4T-1 constructs. To prepare these electrocompetent cells, the *E*. *coli* BL21 DE3 strains containing pACYDuet-1 were used to inoculate a 5 ml culture of lysogeny broth (LB) supplemented with 34 μg/ml chloramphenicol. This 5 ml culture was grown overnight at 37°C. Two ml of the overnight culture were used to inoculate 30 ml of fresh LB supplemented with 34 μg/ml chloramphenicol. This second culture was incubated at 37°C with shaking until an OD_600_ of 0.5–0.7 was reached. The culture was then pelleted at 6,000 x *g* for 6 minutes at 4°C to harvest cells and washed twice with 10 ml cold sterile water. The final pellet was resuspended in sterile water and aliquoted for electroporation, and the cells were next electroporated with the respective pGEX-4T-1 constructs. The cells were then recovered in one ml LB at 37°C for 15–20 minutes. The strains were selected by streaking the recovered cells onto LB agar plates containing 100 μg/ml ampicillin sulfate and 34 μg/ml chloramphenicol. The resultant strains containing both constructs were used for protein co-expression.

### Co-expression of 6xHis-tagged and GST-tagged proteins in culture

Co-expression cultures were supplemented with both 34 μg/ml chloramphenicol and 100 μg/ml ampicillin. A 30 ml starter culture for strains containing both the 6xHis-tag and GST-tag constructs was incubated overnight at 37°C. This starter was used to inoculate a 250 ml culture, which was then grown to an OD_600_ of 0.5–1 before co-expression was induced by adding IPTG to a 1 mM concentration. Cultures were subsequently grown for an additional 3 hours before cells were harvested by centrifugation at 8,200 x *g* for 10 minutes at 4°C and the cell pellets frozen for later protein purification. For BbDUF490/POTRA experiments and respective BbOspC/POTRA controls, a starter volume of 150 ml was incubated overnight at 23°C. Then, 120 ml from the second culture was used to inoculate 1L. The 1L culture was grown at 23°C to an OD_600_ of 0.5–1 before co-expression was induced as with the other cultures. This larger, lower temperature culture was utilized for BbDUF490 co-expression experiments due to the insolubility of BbDUF490 expressed at 37°C. The exception was the BbPOTRA3 experiments which utilized the 250 ml induction cultures at 23°C.

### GST protein purification for co-purification assays

The frozen cell pellets were thawed on ice and resuspended in lysis buffer, which consists of 0.1 x PBS, 50 mM EDTA, 1 mM DTT, and 1:1,000 protease inhibitor cocktail. The resuspended cells were mixed with 10% Triton X-100 to a final concentration of approximately 1% Triton X-100. The cells were then lysed by sonication and subjected to centrifugation at 12,000 x *g* for 30 minutes at 4°C to remove cellular debris. For each set of experiments, the resultant supernatants were normalized by OD_600_ to the lowest OD_600_ in the set of samples, using lysis buffer as a diluent where necessary. Next, ATP and magnesium sulfate were added to a concentration of 4.5 mM and 10 mM, respectively, and samples were incubated in a 37°C water bath for 10 minutes. The samples were then bound to a volume of 1 ml glutathione agarose (Thermo Fisher Scientific, Waltham, MA) for the smaller cultures or 2 ml for the larger cultures by rocking the tubes end-to-end for 15 minutes at room temperature. The bound agarose was centrifuged at 500 x *g* for 1 minute to recover the unbound supernatant. The beads were then washed 8 times with wash buffer (50 mM Tris-Cl, 300 mM NaCl, 50 mM EDTA, pH 7.5) and twice with 1 X PBS. The bound agarose was then stored at 4°C for SDS-PAGE analysis.

For BbBamA POTRA2 co-expression experiments, the beads were then rocked end-to-end at room temperature with a bead volume of elution buffer (wash buffer with 50 mM reduced glutathione) for 3 minutes before centrifuging at 500 x *g* for 3 minutes to collect the purified protein from the soluble fraction. These elution fractions were then stored at 4°C for SDS-PAGE analysis. This extra step was necessary due to excess unbound BbPOTRA2 precipitating into the agarose matrix.

### SDS-PAGE and immunoblot analysis

All co-purification assays were assessed by SDS-PAGE and immunoblot. The whole cell lysate (WCL) samples were from an aliquot of the clarified and normalized cell lysate taken prior to agarose binding. WCL samples were prepared for SDS-PAGE analysis by mixing with final sample buffer (FSB: 62.5 μM Tris-Cl pH 6.8, 10% v/v glycerol, 5% v/v β-mercaptoethanol, 5% SDS, 0.001% bromophenol blue) at a 2:1 sample:FSB ratio before boiling for 10 minutes. These samples were then loaded in equal volumes onto SDS-PAGE gels with either a 4.5/12.5% stacking/separating gel for most experiments or a 4.5/15% stacking/separating gel for individual POTRA and His-tagged BamD experiments (Figs 4 and 5B).

The bead and elution samples were prepared at a 1:1 90% bead slurry:FSB or a 1:1 elution:FSB ratio before boiling. The exceptions to this were the OspC/Pal samples which required dilution at 1:10 ratio due to high purification yields and DUF490 samples which required a 2:1 ratio due to lower purification yields. The samples were then boiled for 10 minutes and loaded onto the SDS-PAGE gels as necessary for similar GST-tagged protein loading. For BbDUF490 co-purification assays, the volumes were as follows: BbDUF490- 133 X OspC volume, BbDUF490 Segment 1–25 X OspC volume, BbDUF490 Segment 2–35 X OspC volume, and BbDUF490 Segment 3–50 X OspC volume. For StDUF490 purifications, the volumes were as follows: StDUF490- 50 X Pal volume and Pal- 1 X Pal volume. In the control experiment where StDUF490 was purified from StBamA POTRA1-5 co-expression culture and BbOspC co-expression culture in Fig 8C, the volumes loaded between samples were equivalent.

After the SDS-PAGE gels were run, the proteins were transferred to polyvinylidene fluoride (PVDF) membrane for immunoblot analysis. Transfers and immunoblots were performed as previously described [58]. For analysis of His-tagged proteins, the immunoblots were incubated with 1:1,500 HRP-conjugated mouse-anti-His antibody (R&D Systems, Minneapolis, MN) for 75 minutes at room temperature for WCL samples or overnight at 4°C for bead/elution samples. For analysis of GST-tagged proteins, the immunoblots were incubated in 1:2,000 goat-anti-GST primary antibody (Cytiva Life Sciences, Marlborough, MA) at room temperature for 1 hour and subsequently incubated in 1:6,000 HRP-conjugated rabbit-anti-goat secondary antibody (Invitrogen, Waltham, MA) for 45 min at room temperature. The immunoblots were developed using the SuperSignal West Pico PLUS substrate (Thermo Fisher Scientific, Waltham, MA) and imaged using the ChemiDoc MP Imaging System (Bio-Rad, Hercules, CA).

## Supporting information

S1 Fig AlphaFold v2.0 Model of DUF490 Segment 1 and 2 compared to DUF490. Segment 1 and 2 from both models displayed. The structure from the full-length model of DUF490 is depicted in blue and the structure from the Segment 1 and 2 model in green. The structures are oriented with the N-terminus to the right.
(TIF)

S1 File Raw Blot Images for Figs 3, 4, 5, 8 and 9. Labels are as follows: Fig ID. Panel# left to right for Figs 4, 5, 8 and 9. Lane# left to right within panel. Sample Name.
(PDF)

S2 File Commands utilized for *In situ* interaction model. (PDF)

S3 File Files used by RosettaCMs. Available in repository at https://simtk.org/projects/bama_tamb_bb.
(DOCX)
