# Supplementary figures and images for: A conserved C-terminal domain of TamB interacts with multiple BamA POTRA domains in Borreliella burgdorferi
Source: PLoS One. 2024 Aug 29;19(8):e0304839. doi: 10.1371/journal.pone.0304839 (PMC11361582; doi:10.1371/journal.pone.0304839)

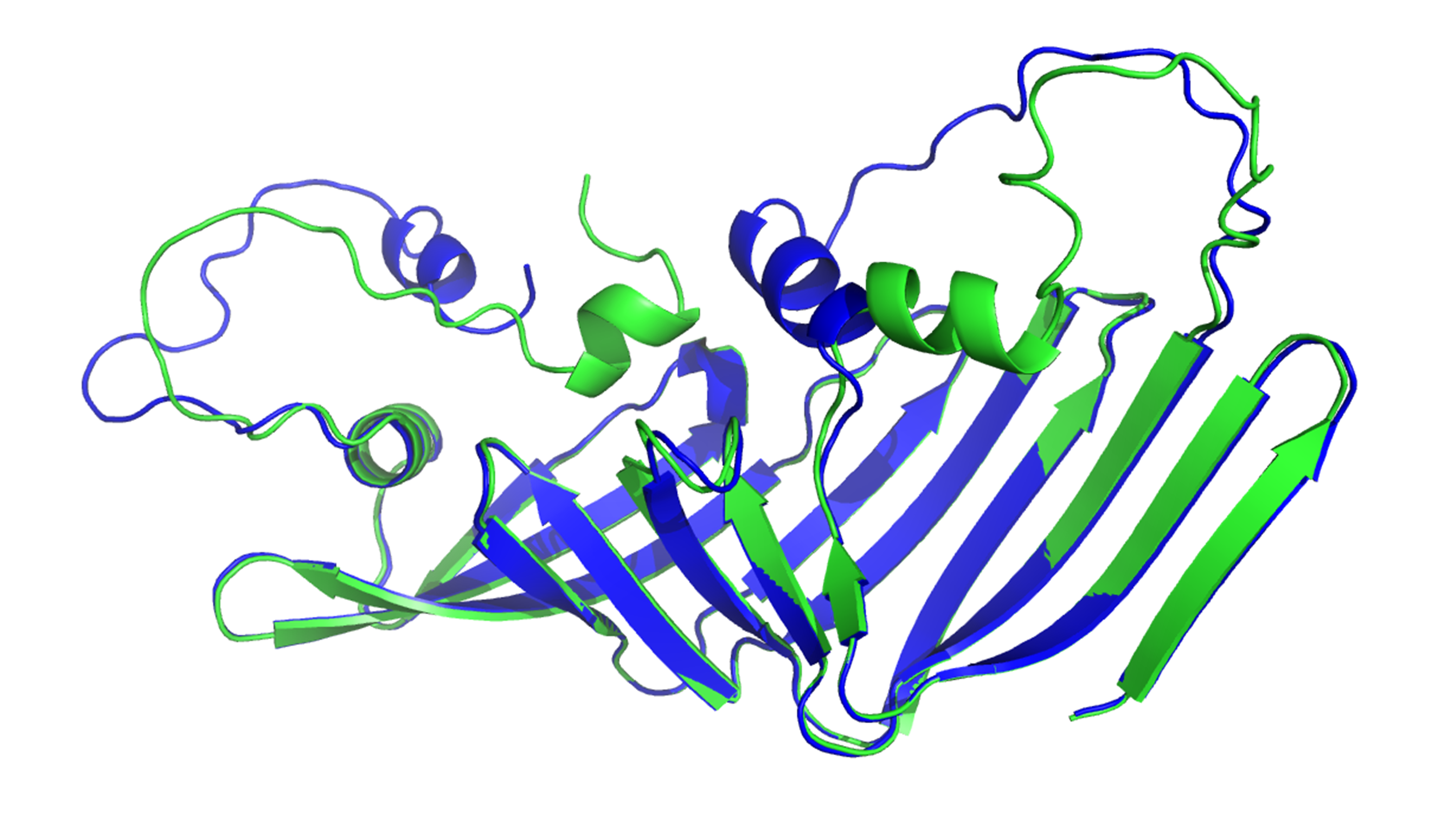

Supplement: S1 Fig — Segment 1 and 2 from both models displayed. The structure from the full-length model of DUF490 is depicted in blue and the structure from the Segment 1 and 2 model in green. The structures are oriented with the N-terminus to the right. (TIF) [file pone.0304839.s001.tif]
