# Supplementary material for: A conserved C-terminal domain of TamB interacts with multiple BamA POTRA domains in Borreliella burgdorferi
Source: PLoS One. 2024 Aug 29;19(8):e0304839. doi: 10.1371/journal.pone.0304839 (PMC11361582; doi:10.1371/journal.pone.0304839)

All images are whole-membrane images of PVDF membranes, collected using chemiluminescent imaging on a BioRad ChemiDoc MP Imaging System.

Samples not pertinent to the figure, as indicated above the image in bold, are marked with an ‘X’ above the lane.

BbBamA POTRA1-5 WCL anti-GST
Figure 3

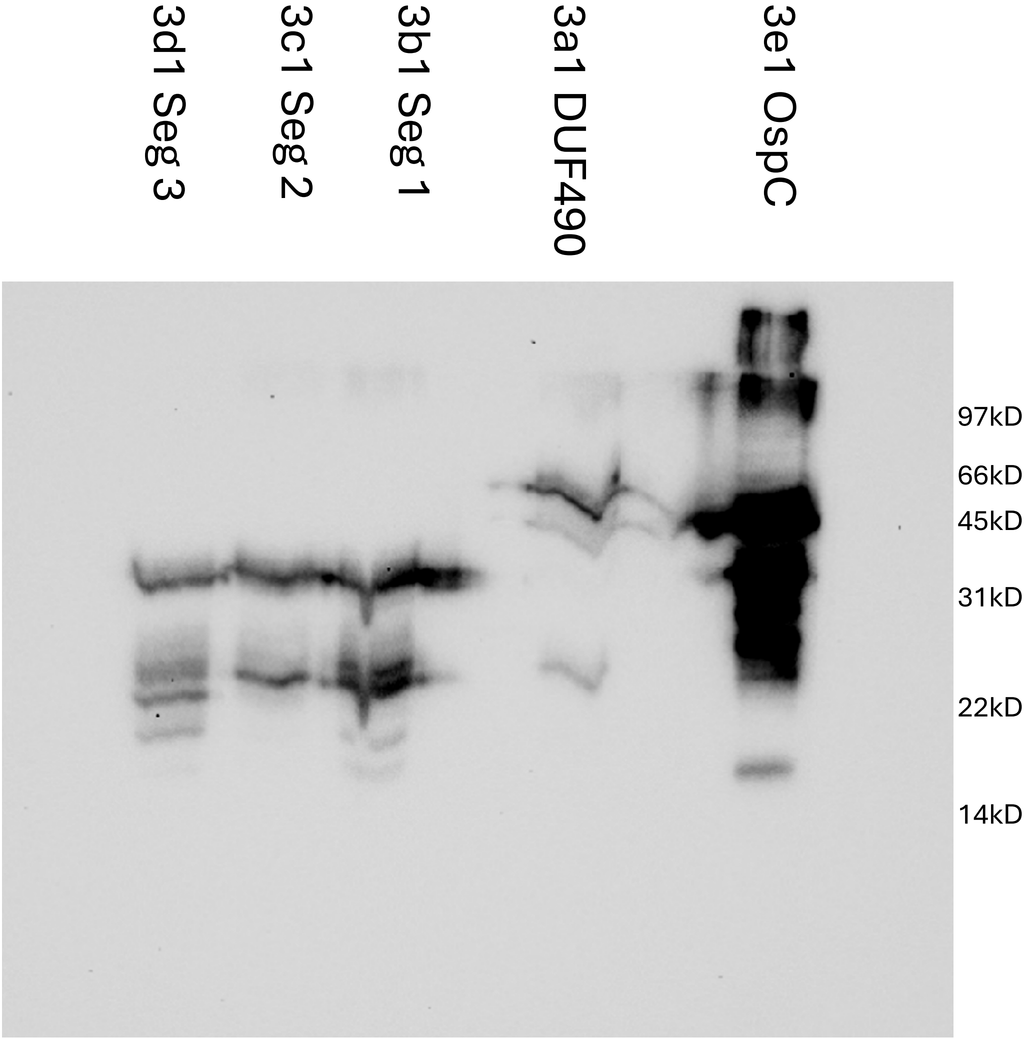

BbBamA POTRA1-5 WCL anti-His
Figure 3

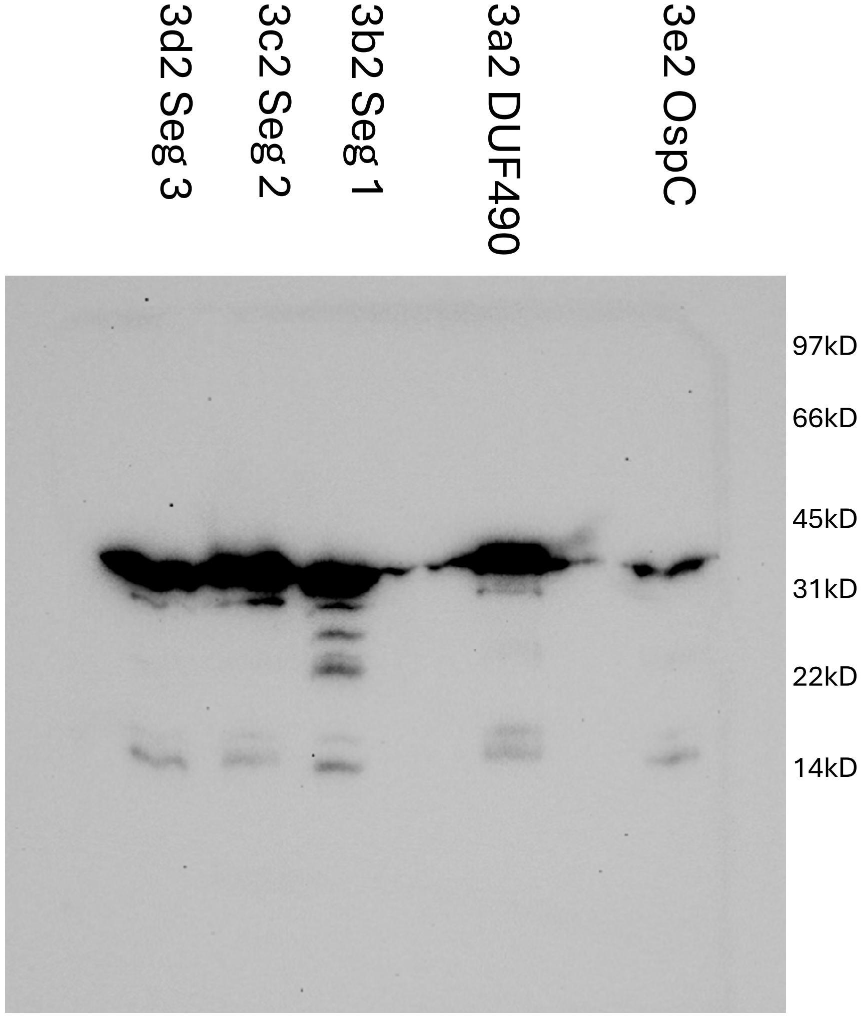

BbBamA POTRA1-5 Purification anti-GST
Figure 3

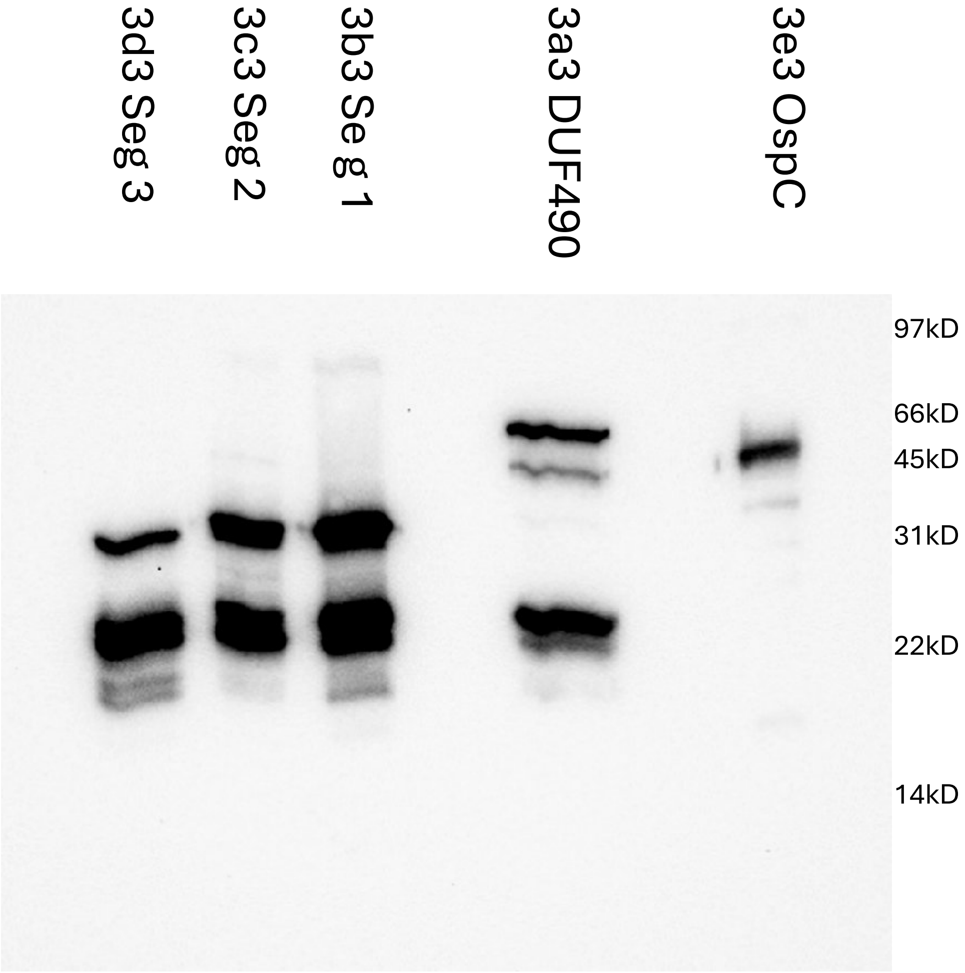

BbBamA POTRA1-5 Purification anti-His
Figure 3

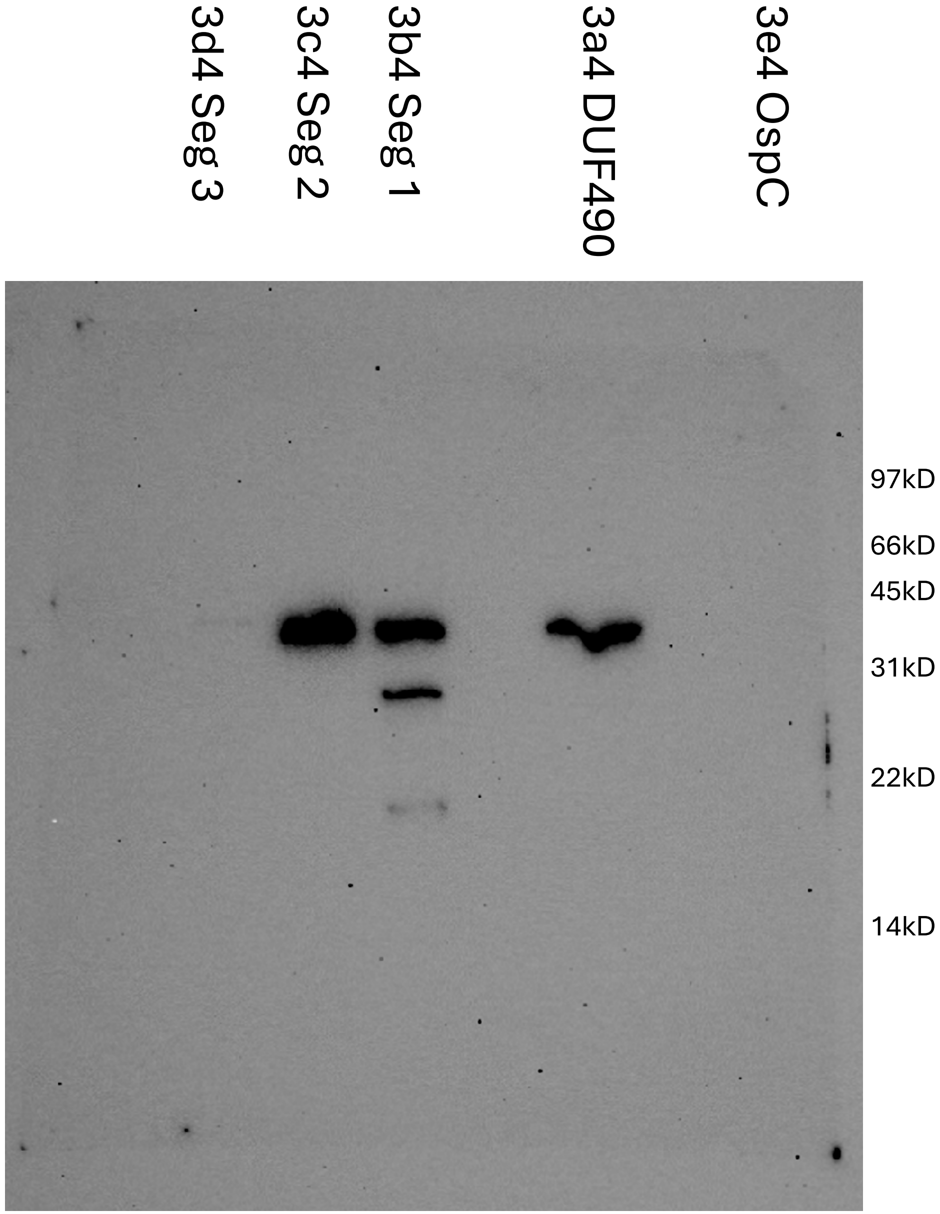

BbBamA POTRA1 WCL anti-GST
Figure 4a

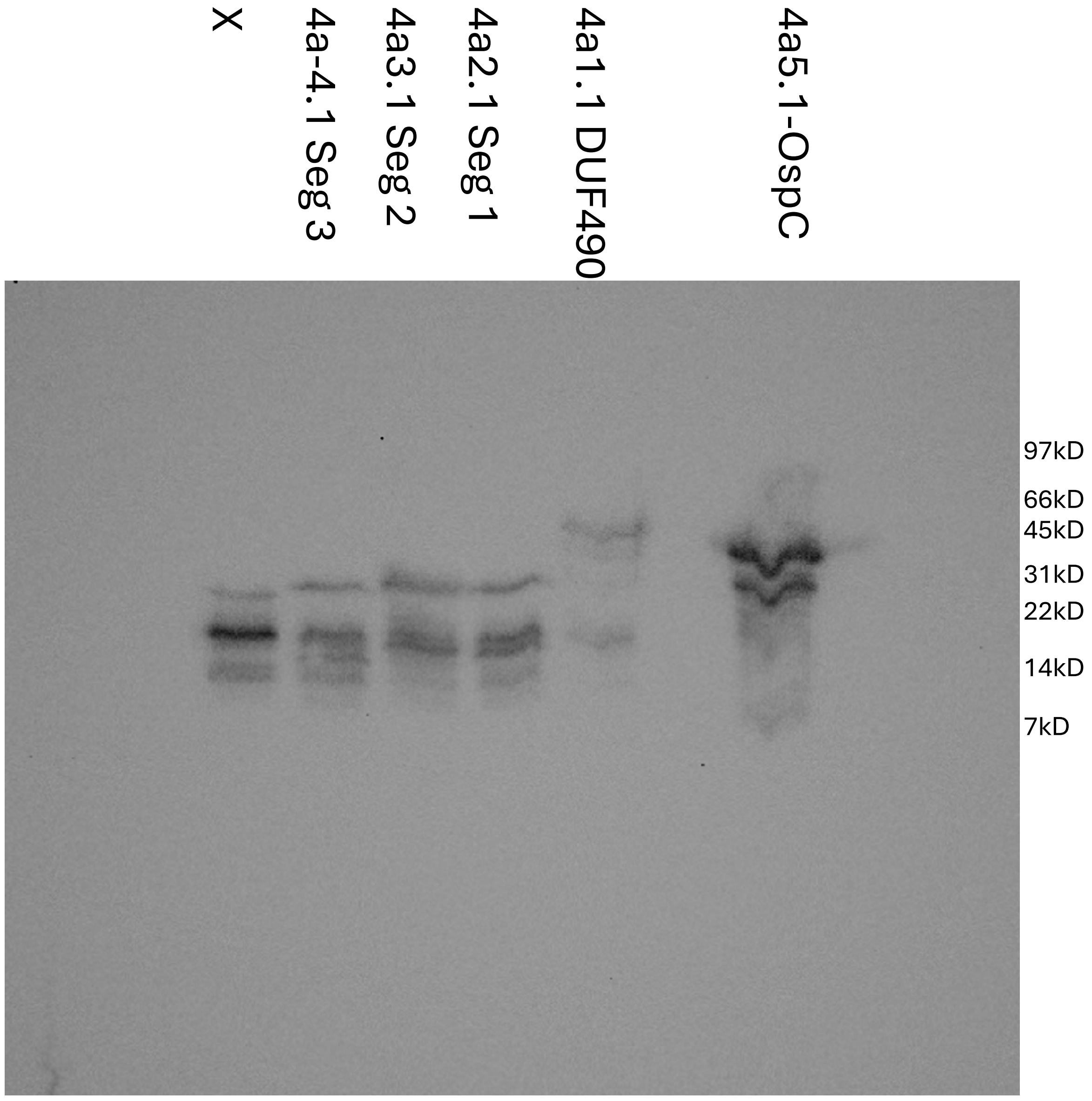

BbBamA POTRA1 WCL anti-His
Figure 4a

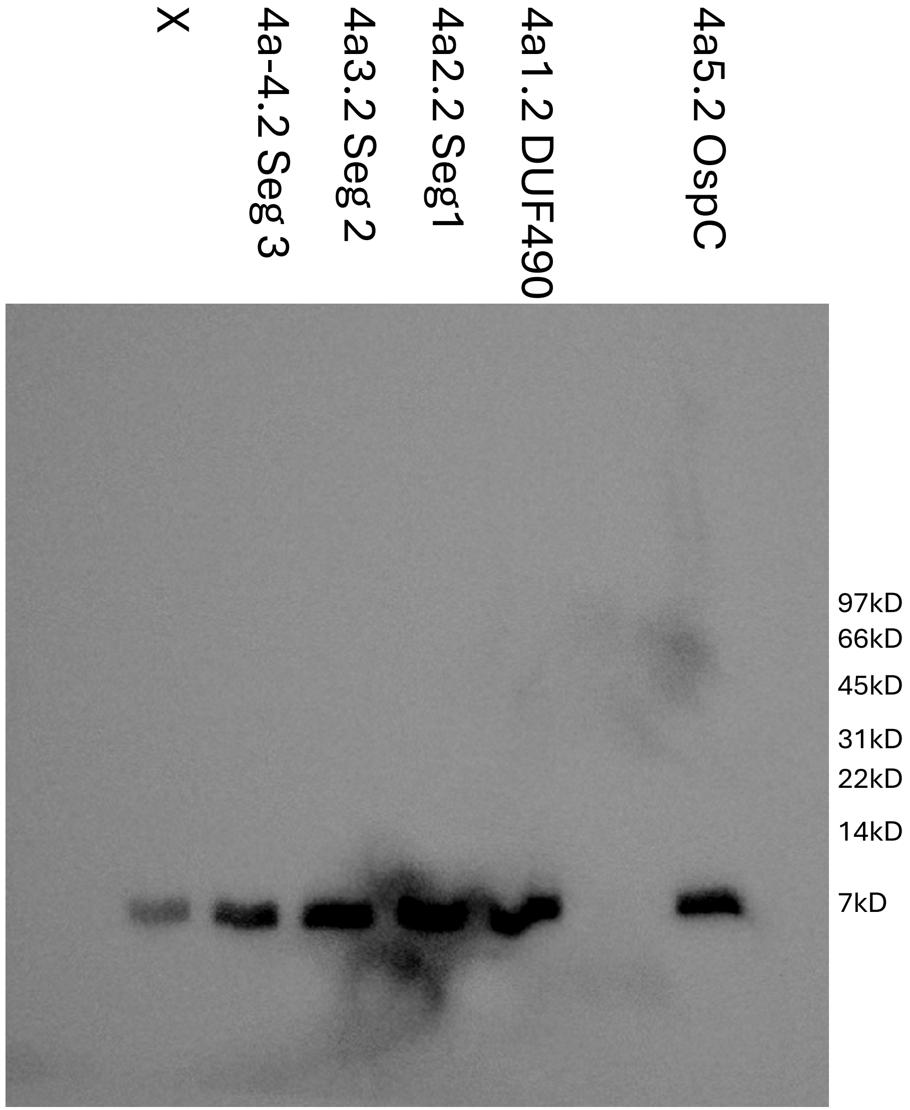

BbBamA POTRA1 Purification anti-GST
Figure 4a

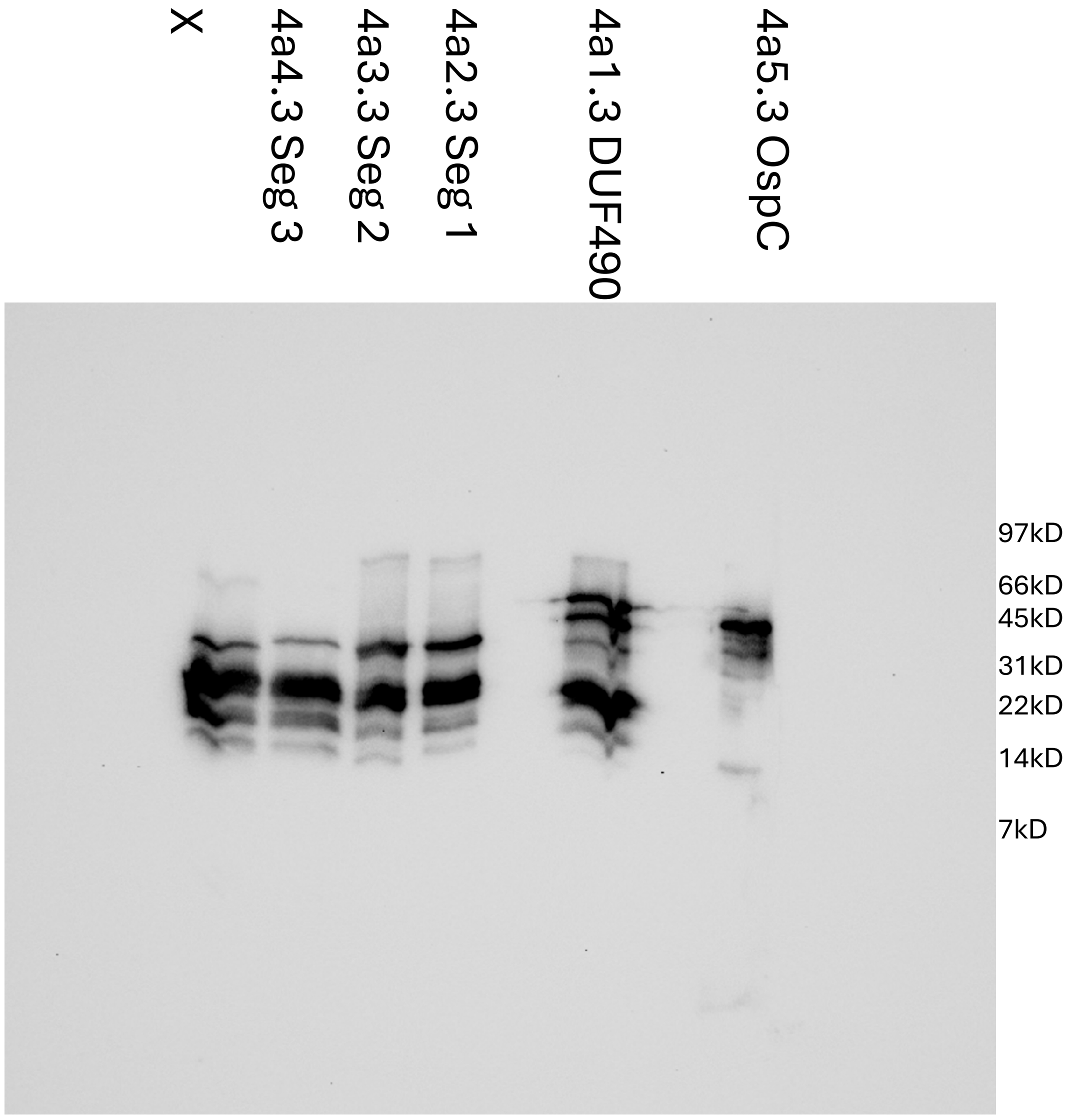

BbBamA POTRA1 Purification anti-His
Figure 4a

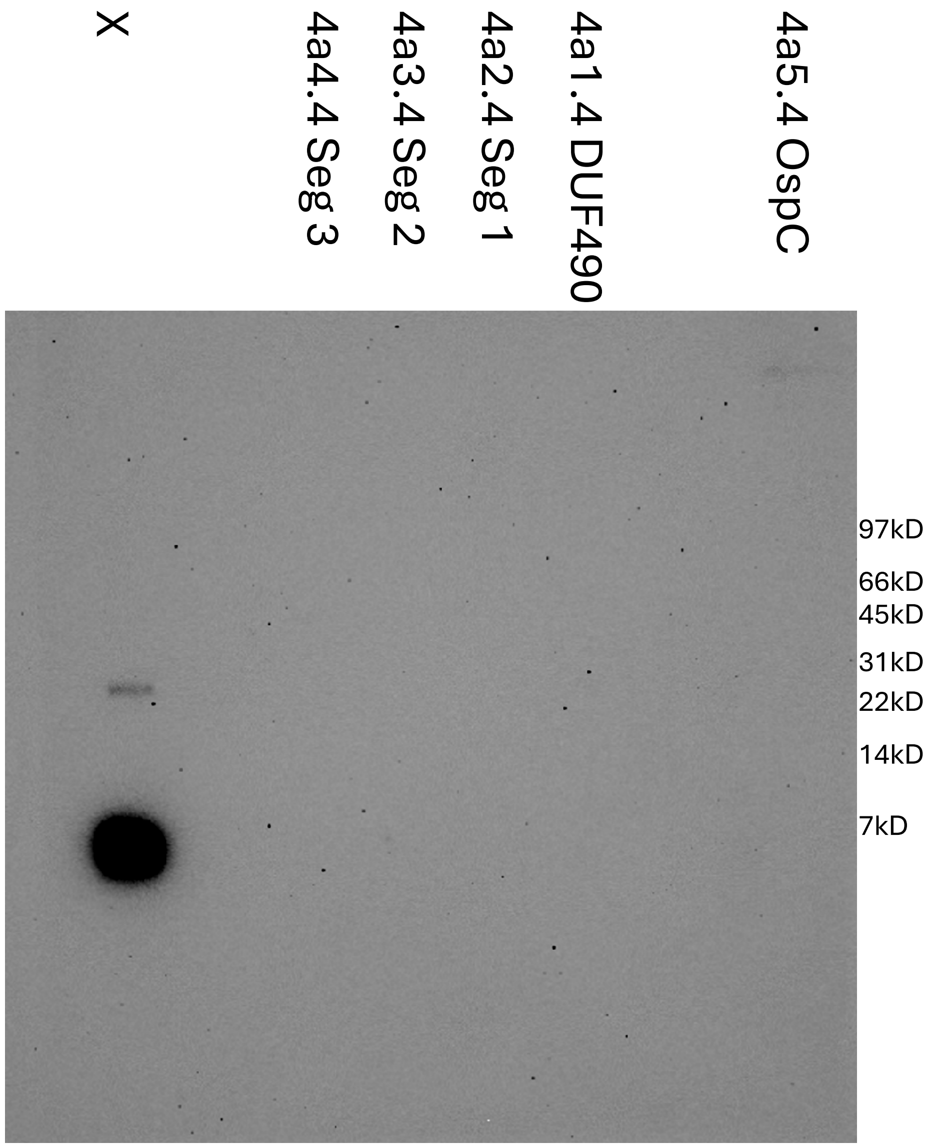

BbBamA POTRA2 WCL Anti-GST
Figure 4b

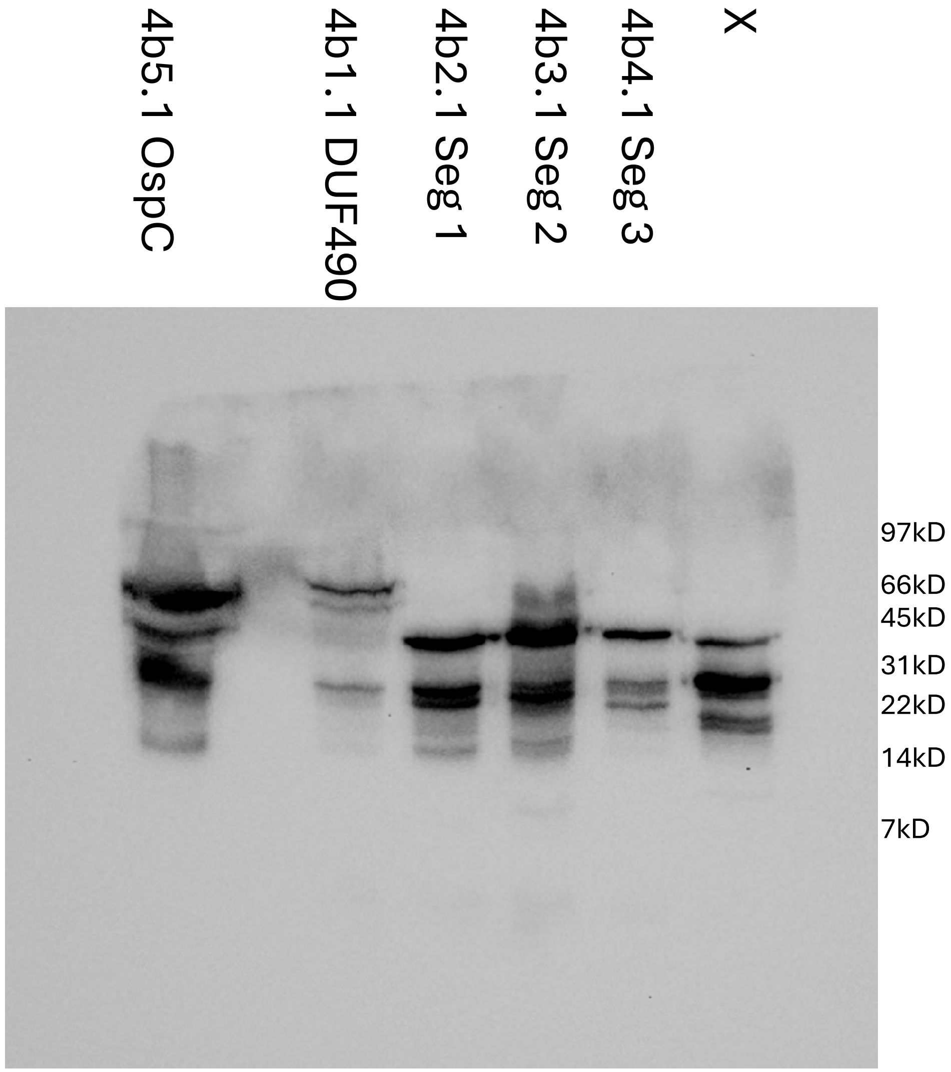

BbBamA POTRA2 WCL Anti-His
Figure 4b

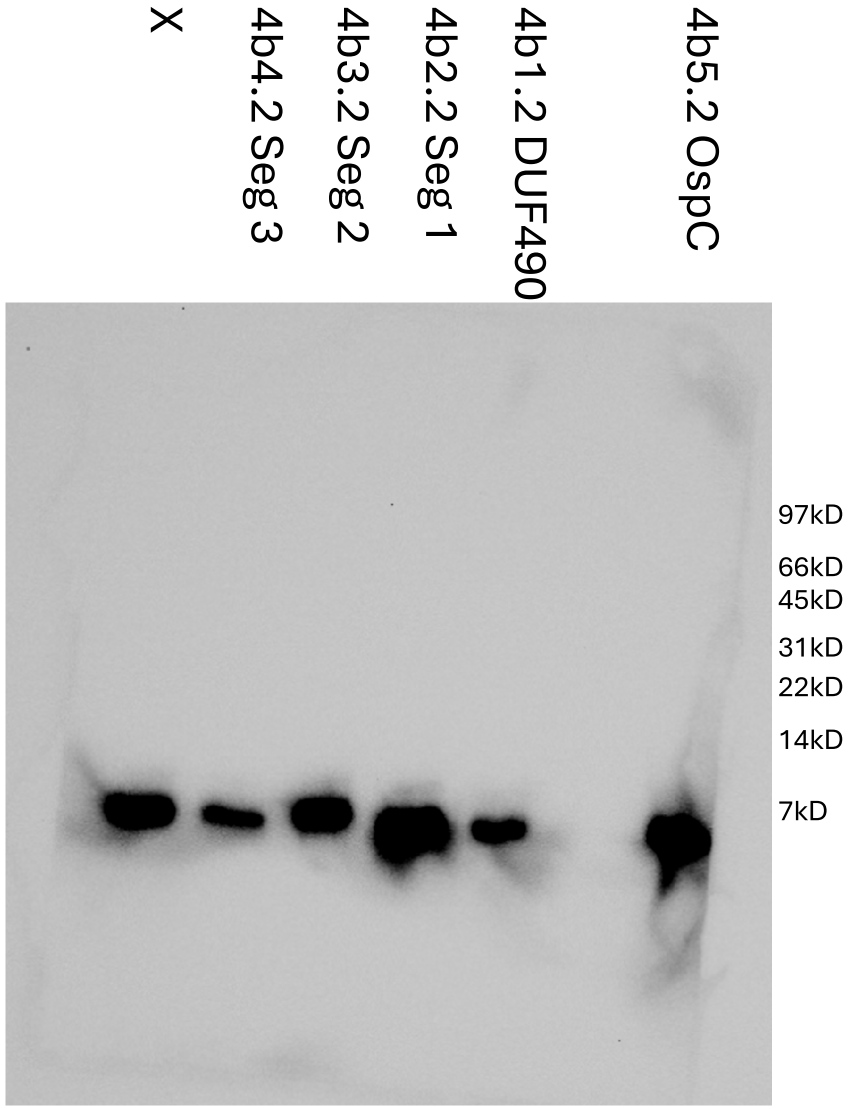

BbBamA POTRA2 Purification anti-GST
Figure 4b

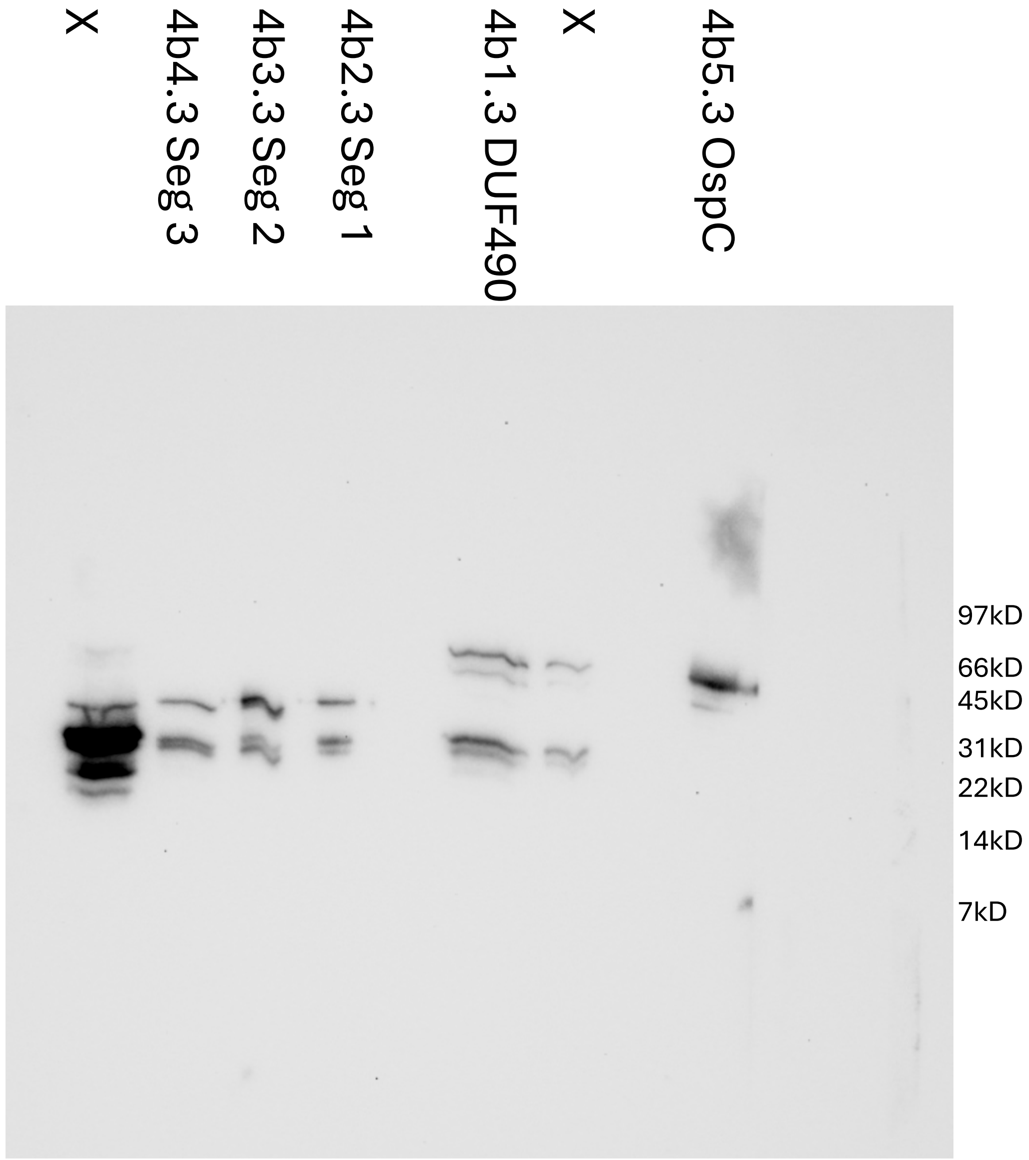

BbBamA POTRA2 Purification anti-His
Figure 4b

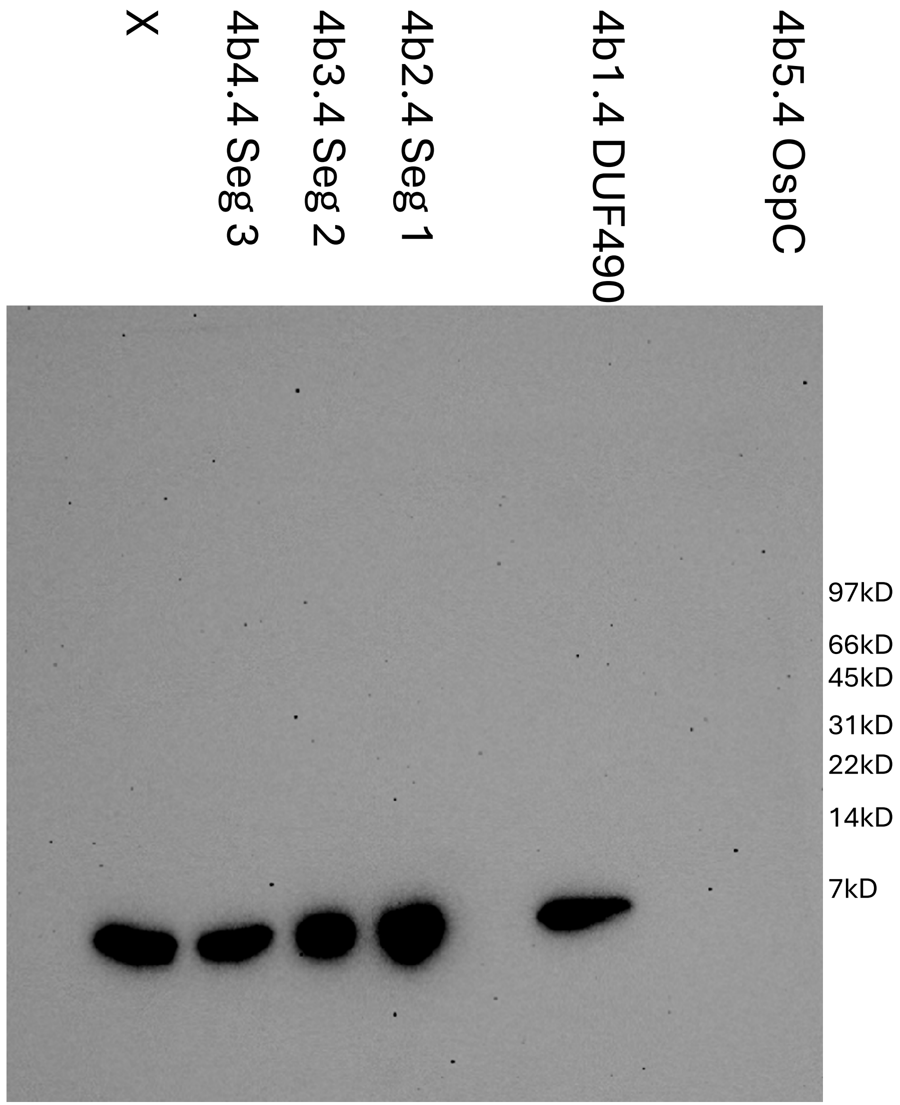

BbBamA POTRA3 WCL anti-GST Figure 4c

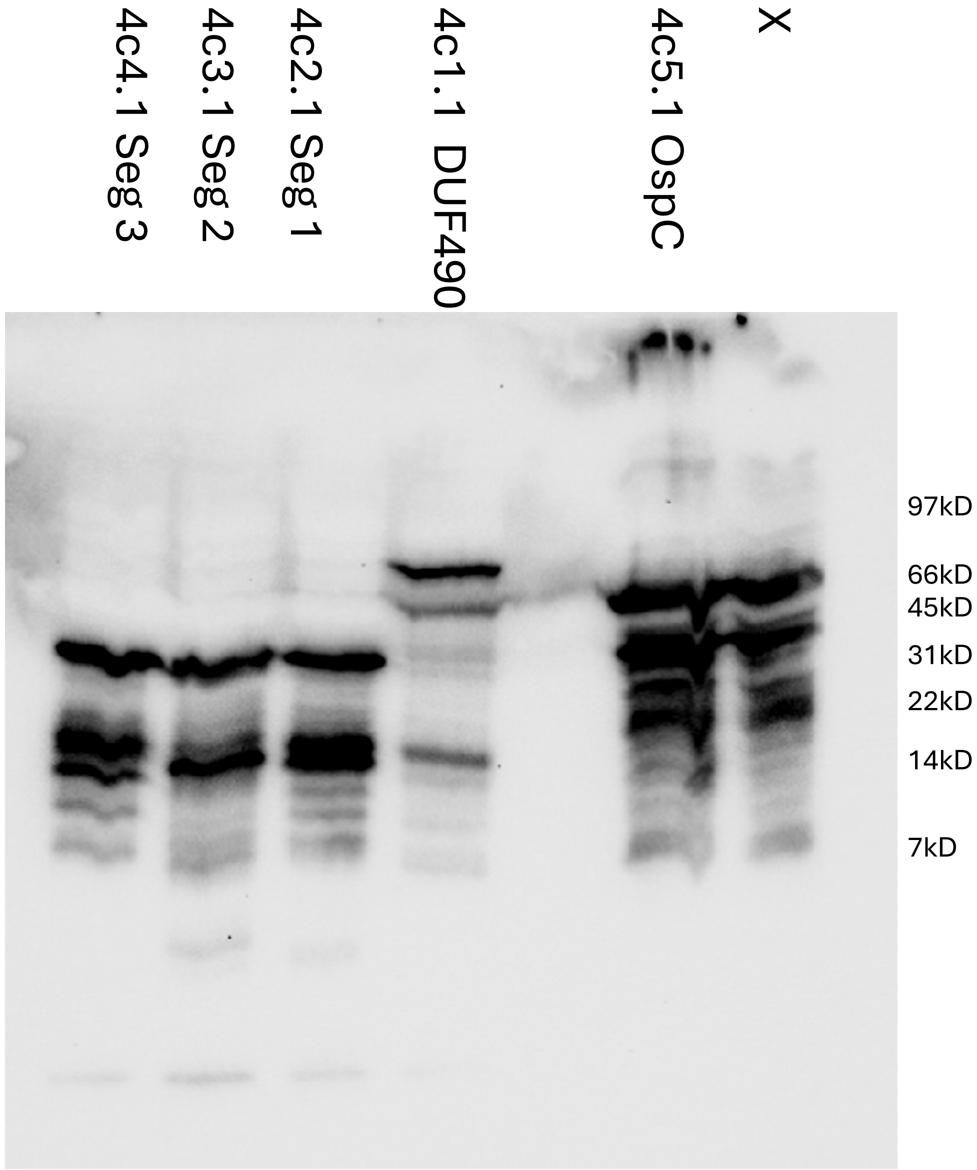

BbBamA POTRA3 WCL anti-His Figure 4c

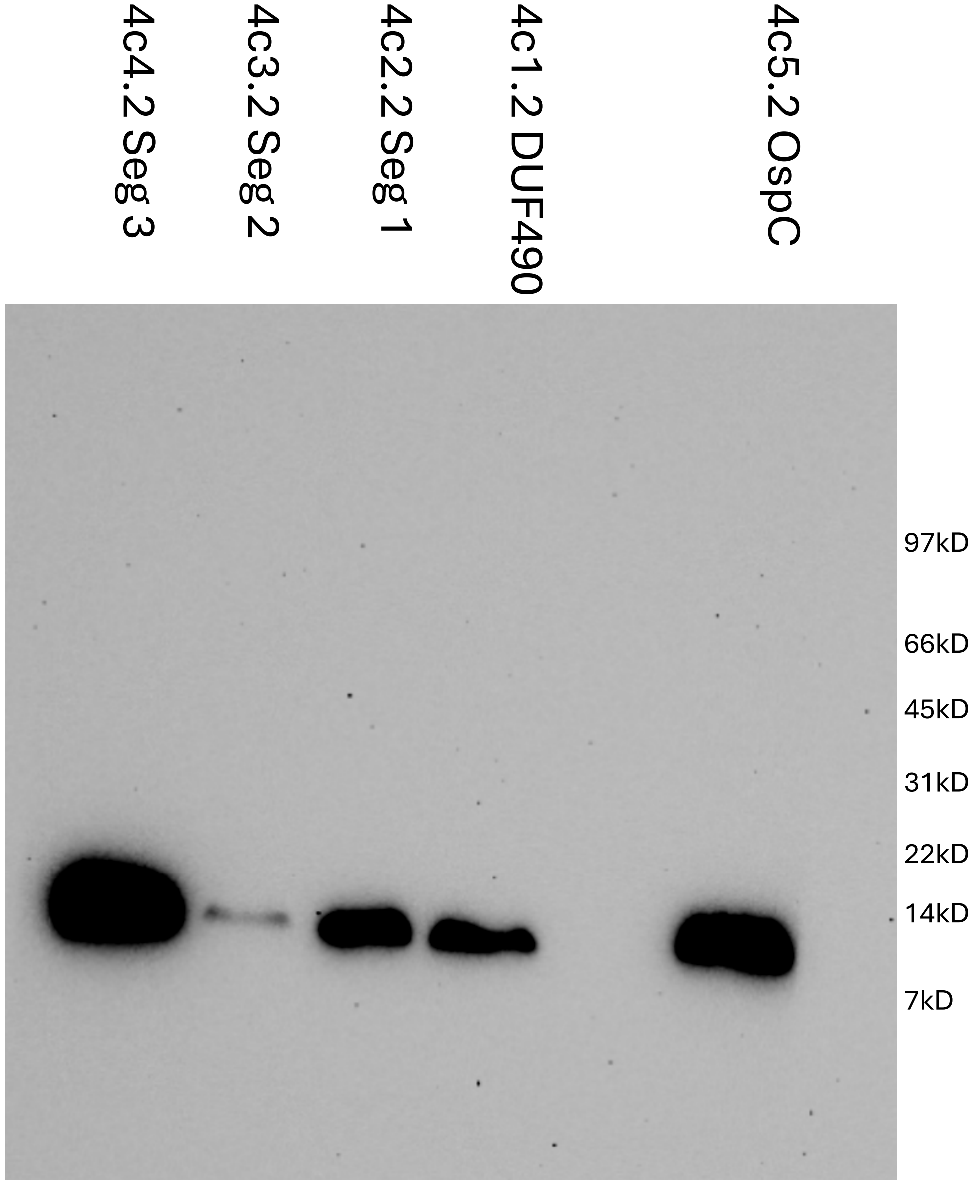

BbBamA POTRA3 Purification anti-GST
Figure 4c

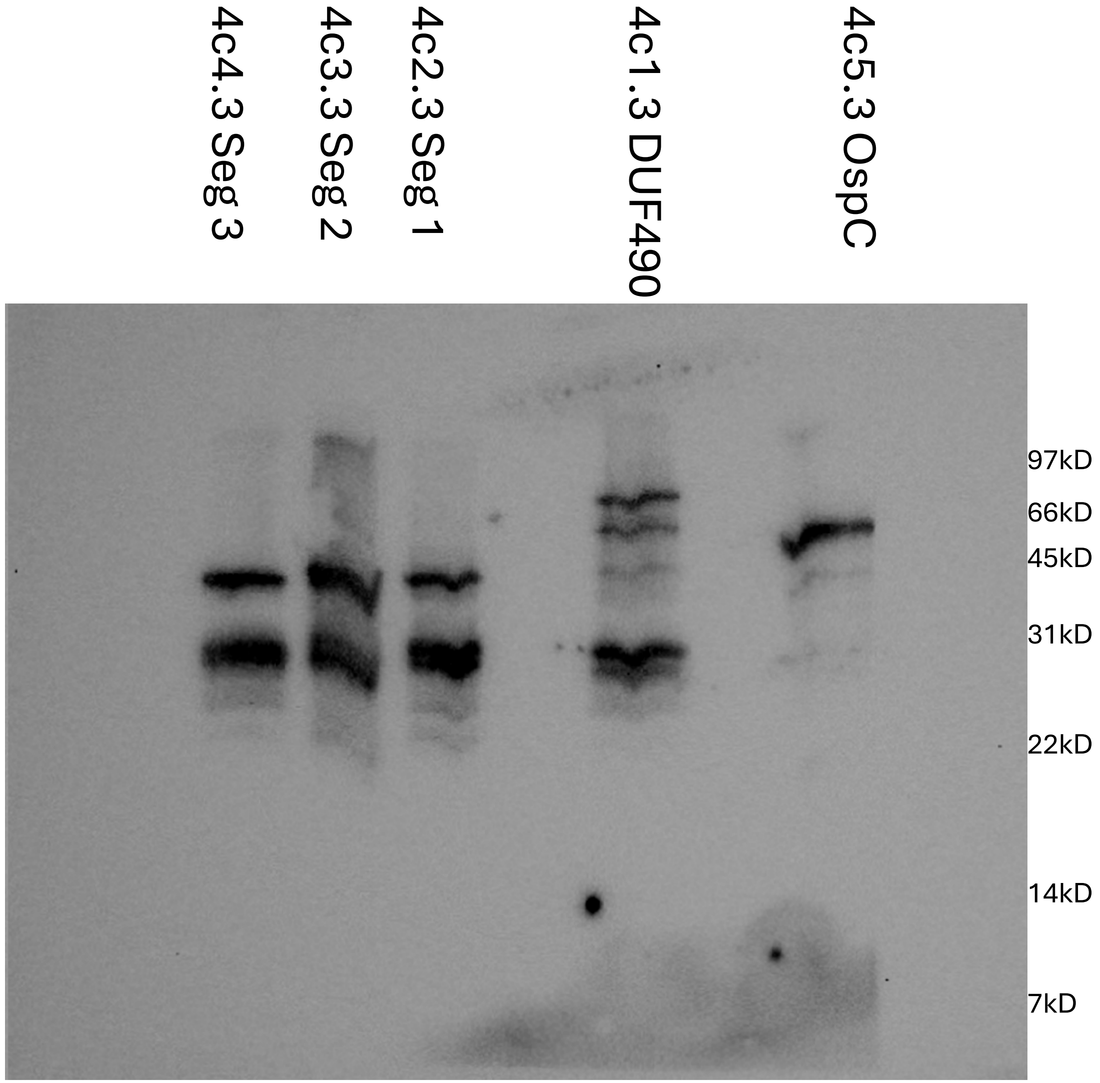

BbBamA POTRA3 Purification anti-His
Figure 4c

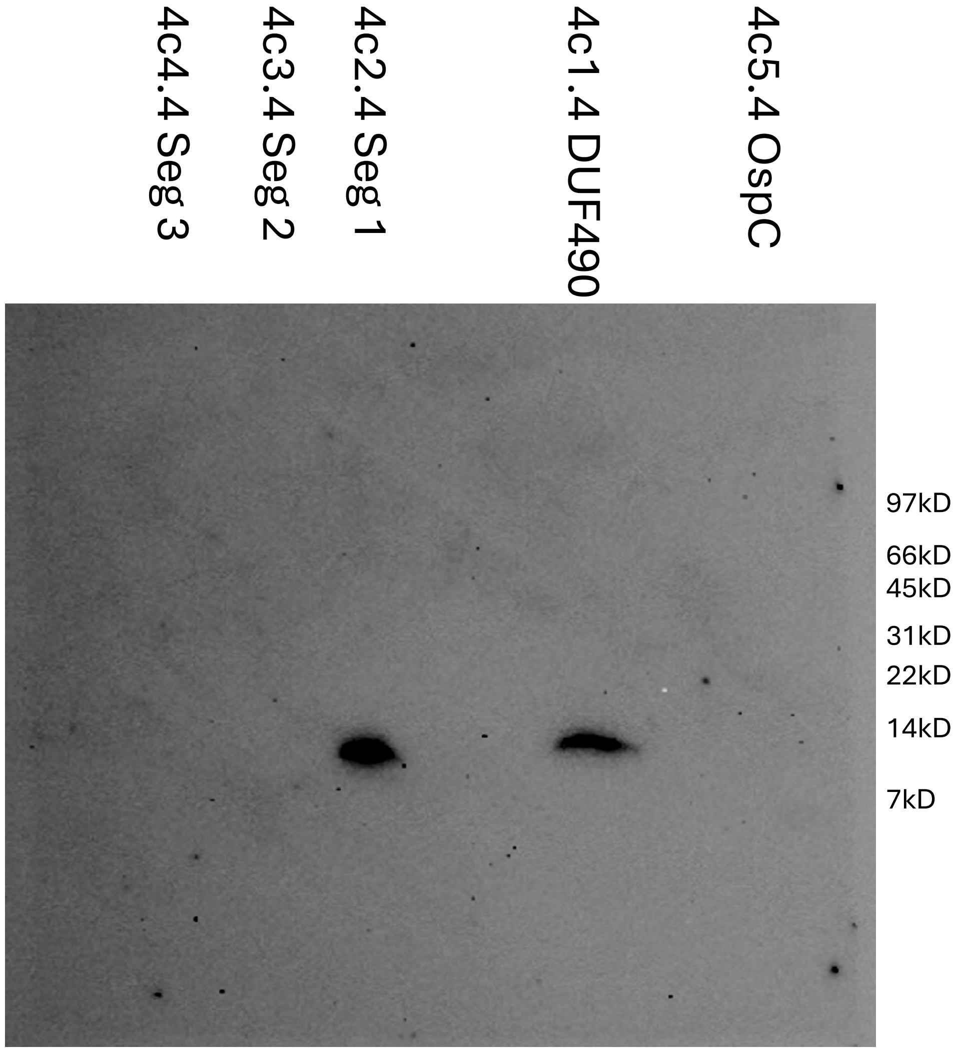

BbBamA POTRA4 WCL anti-GST
Figure 4d

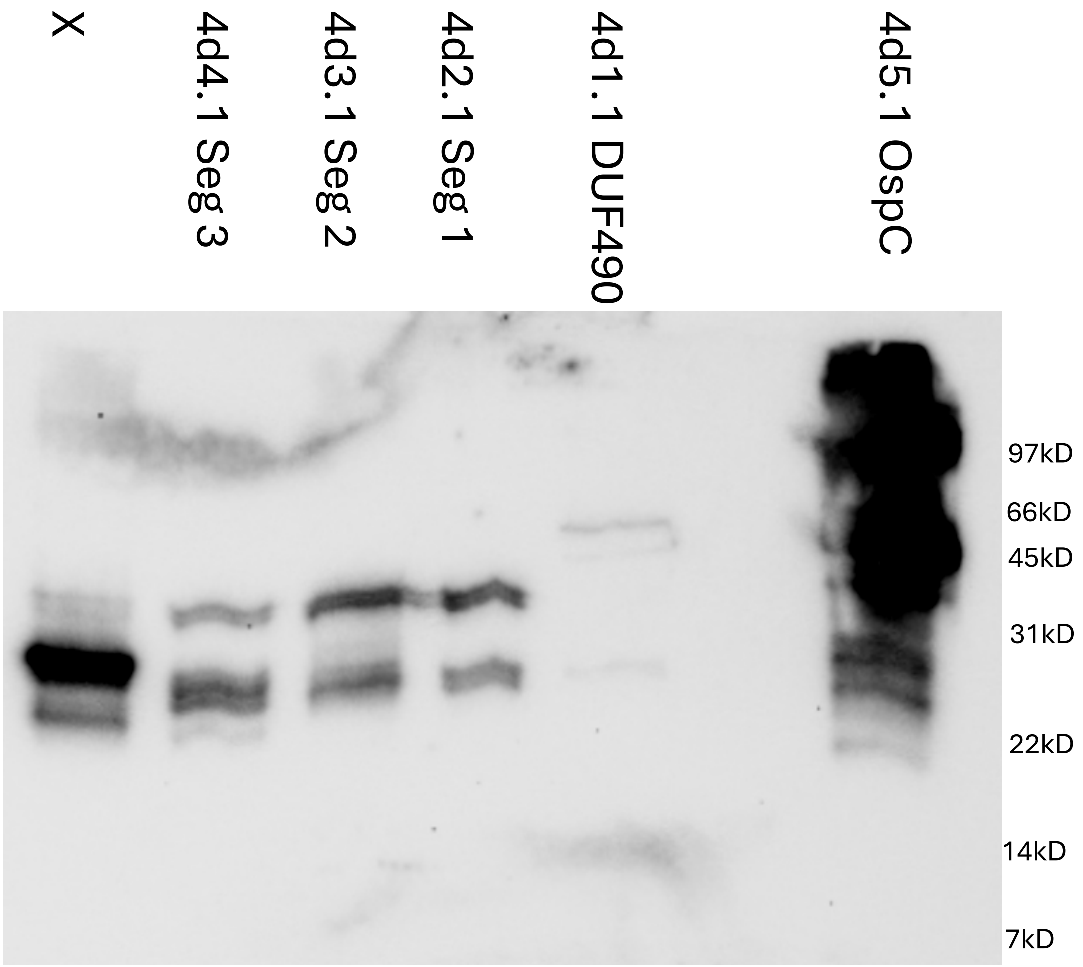

BbBamA POTRA4 WCL anti-His
Figure 4d

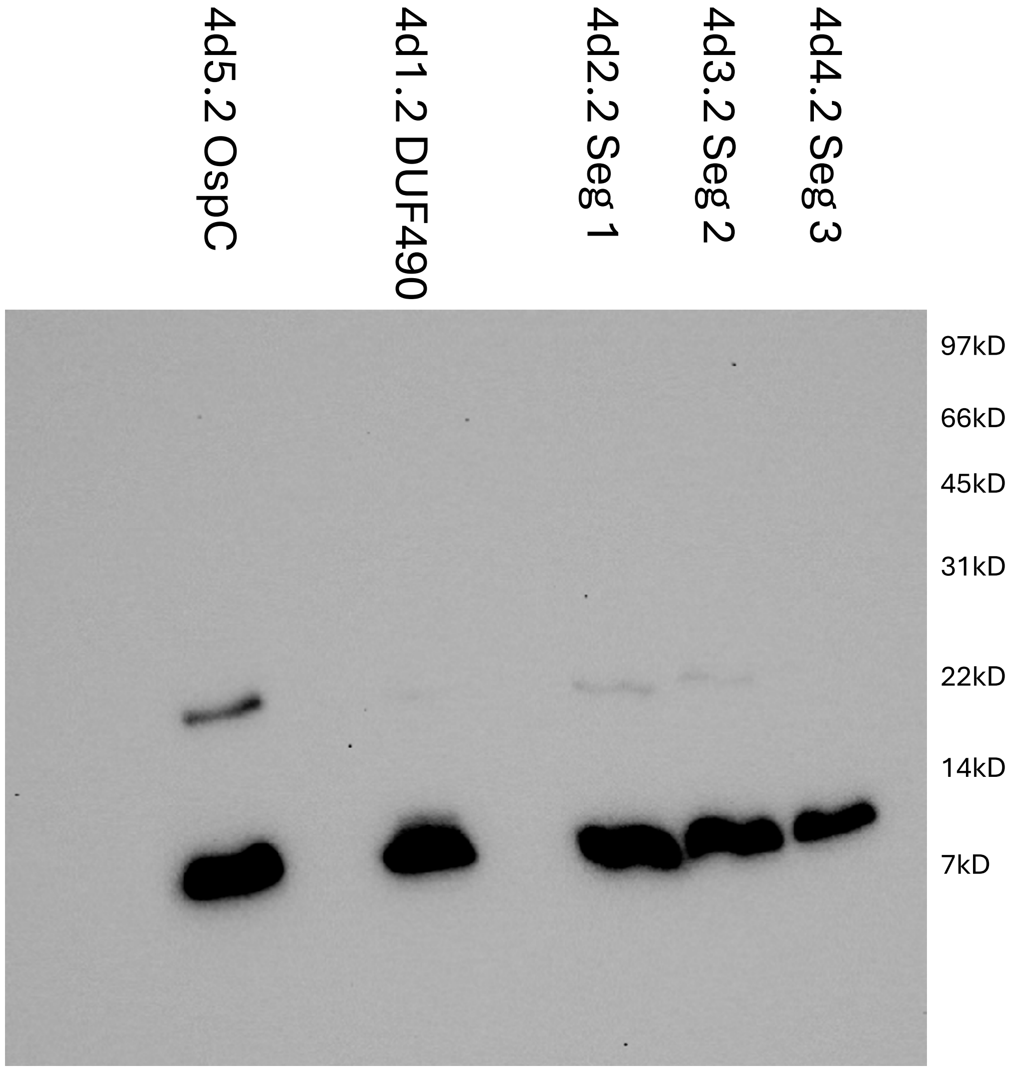

BbBamA POTRA4 Purification anti-GST
Figure 4d

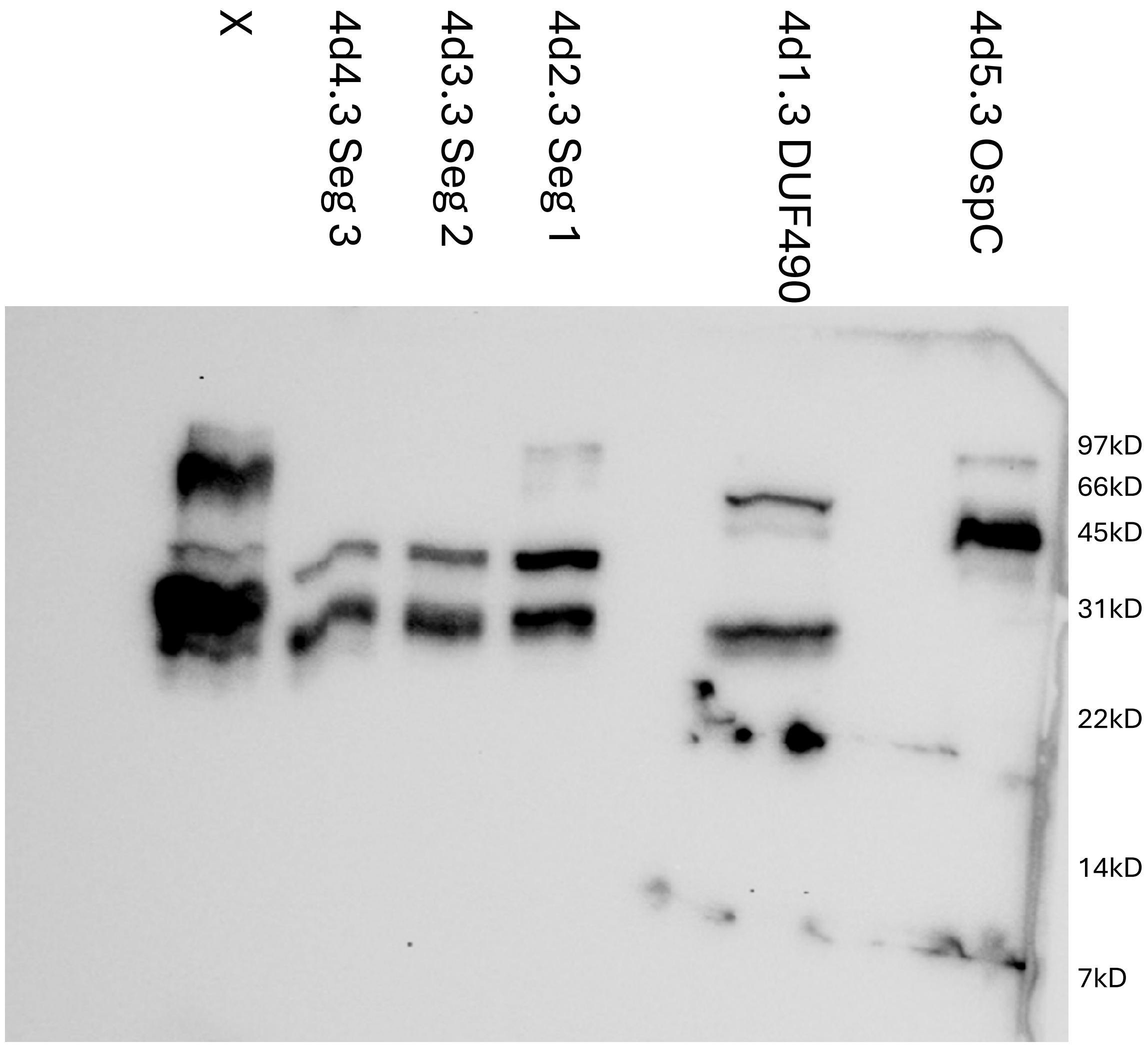

BbBamA POTRA4 Purification anti-His
Figure 4d

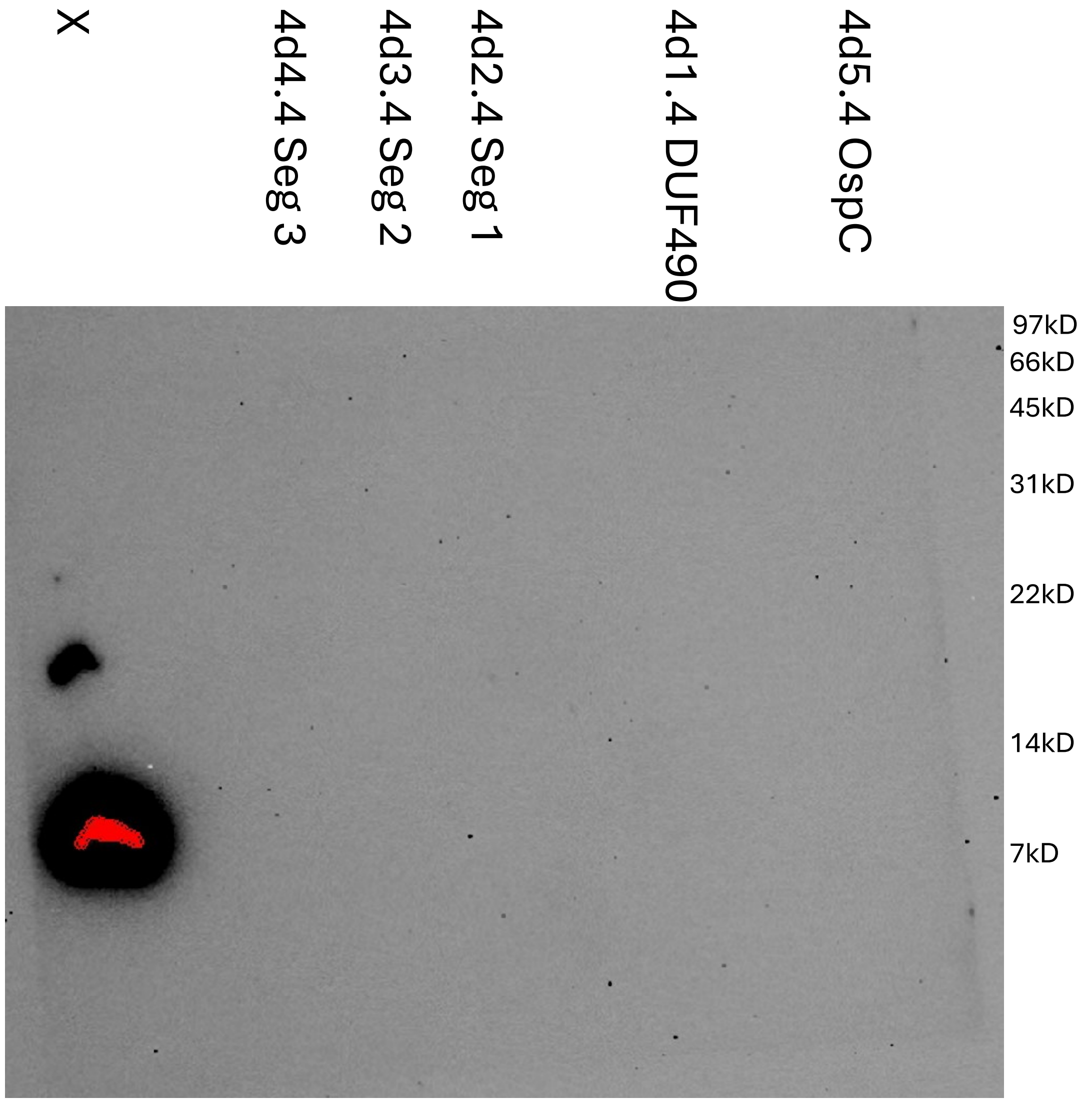

BbBamA POTRA5 WCL anti-GST
Figure 4e

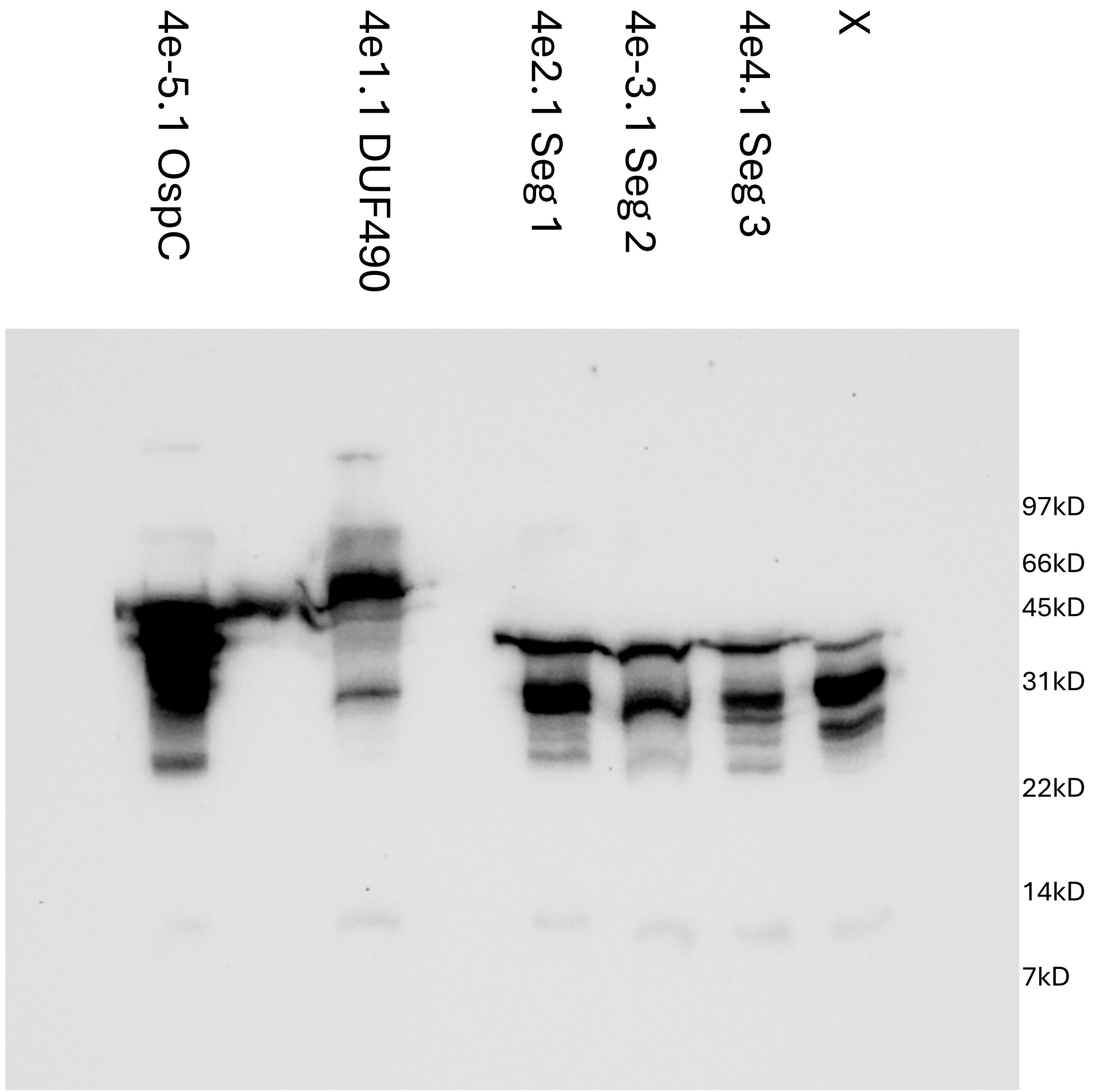

BbBamA POTRA5 WCL anti-His
Figure 4e

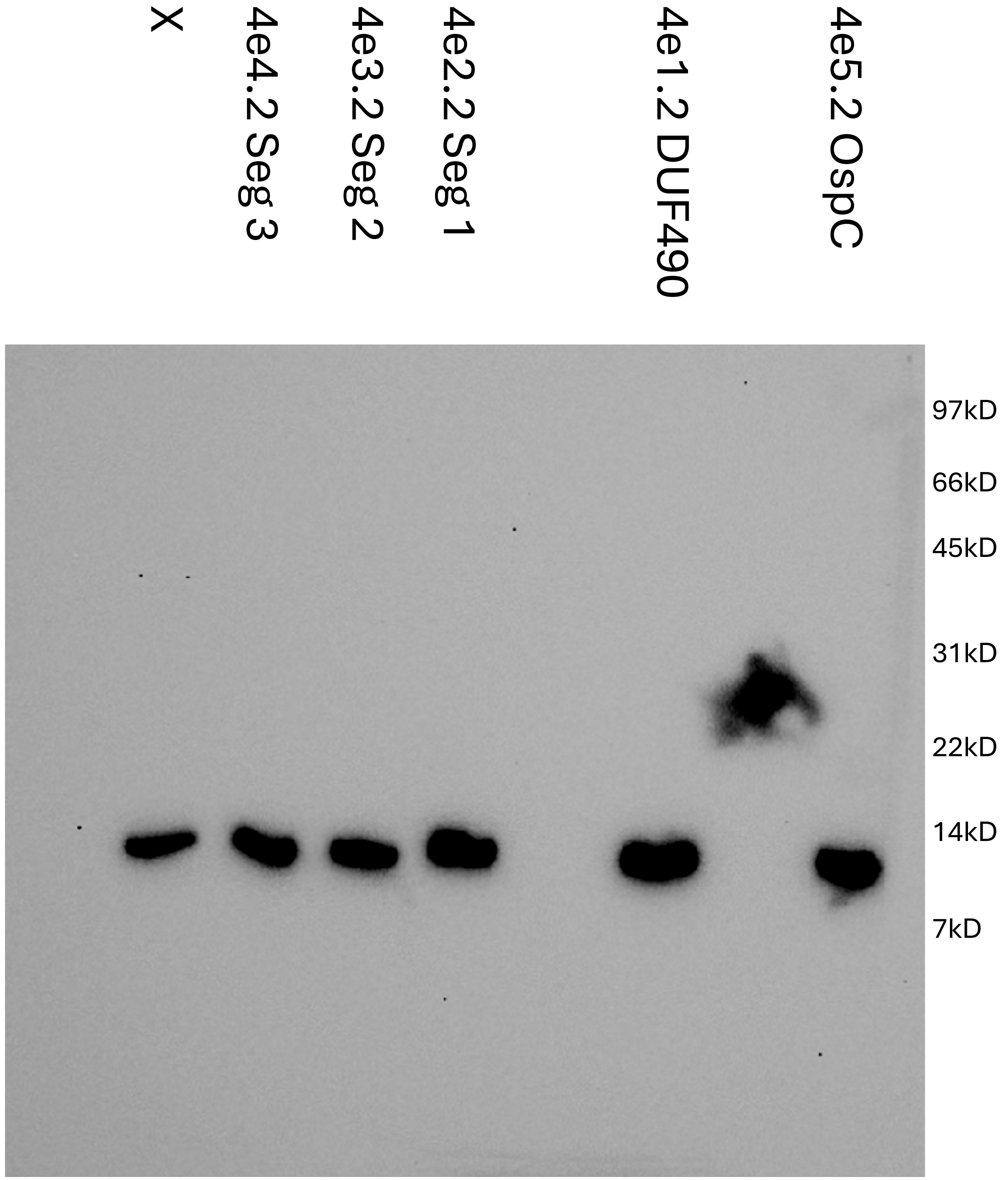

BbBamA POTRA5 Purification anti-GST
Figure 4e

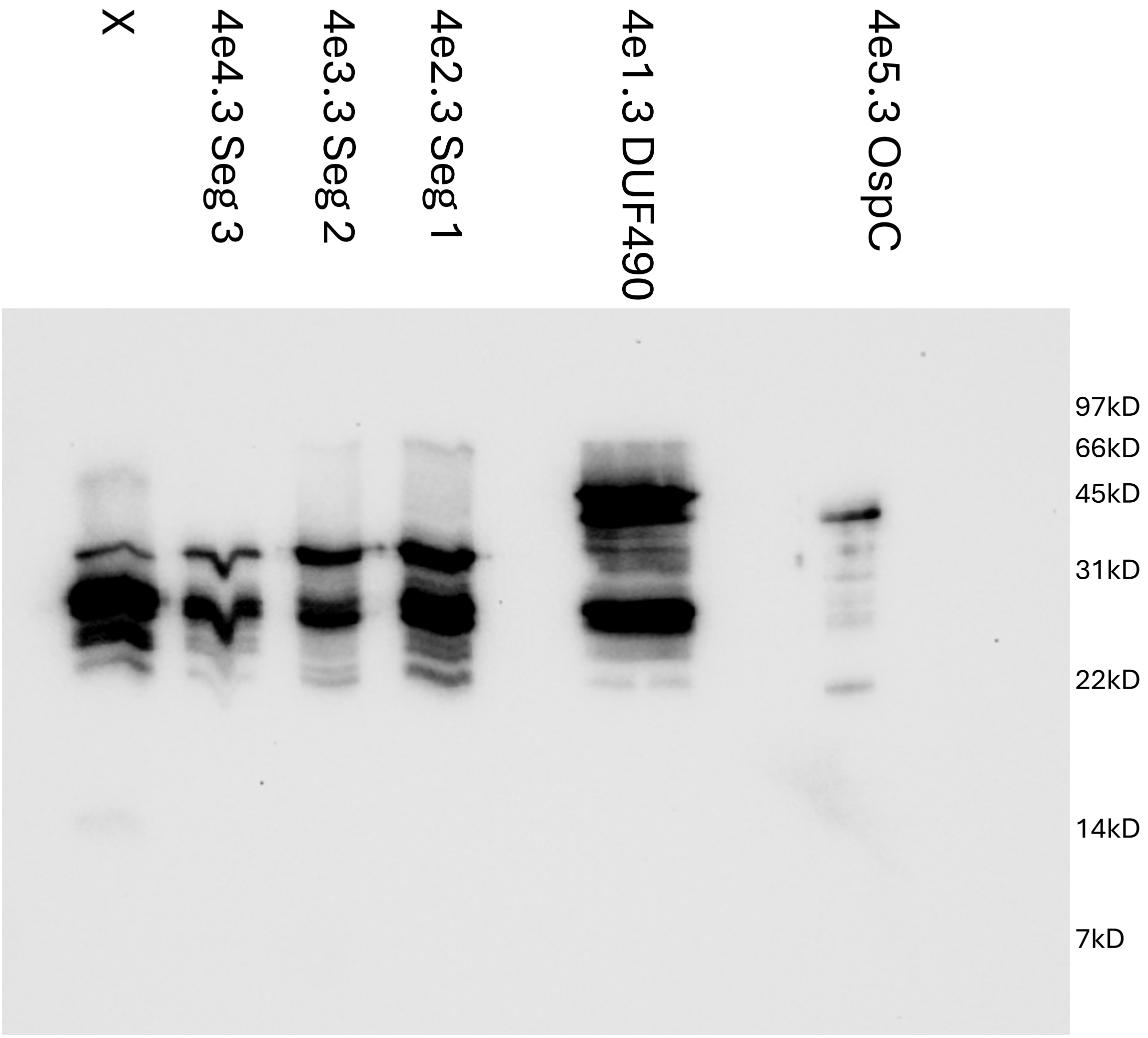

BbBamA POTRA5 Purification anti-His
Figure 4e

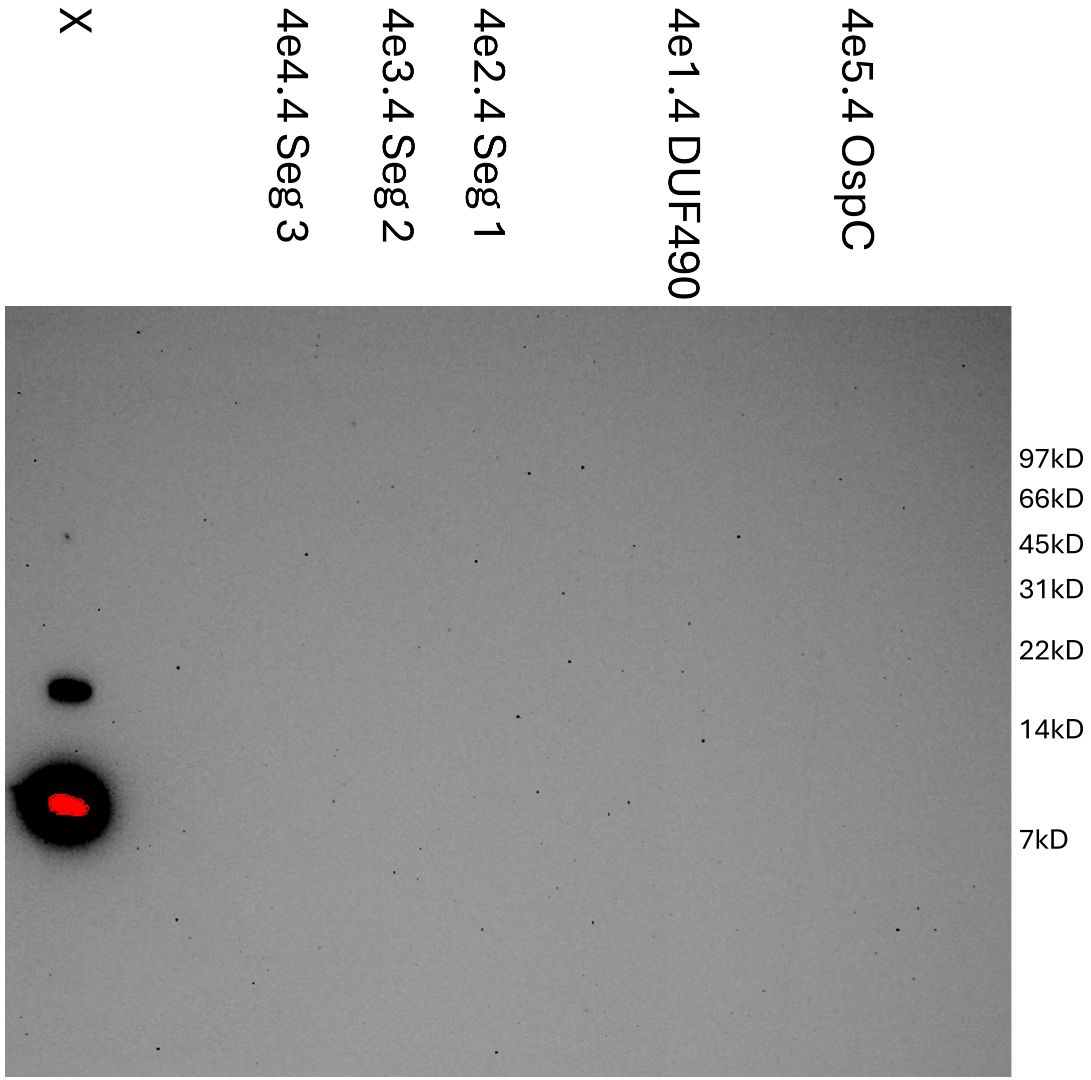

BbBamB WCL anti-GST
Figure 5a

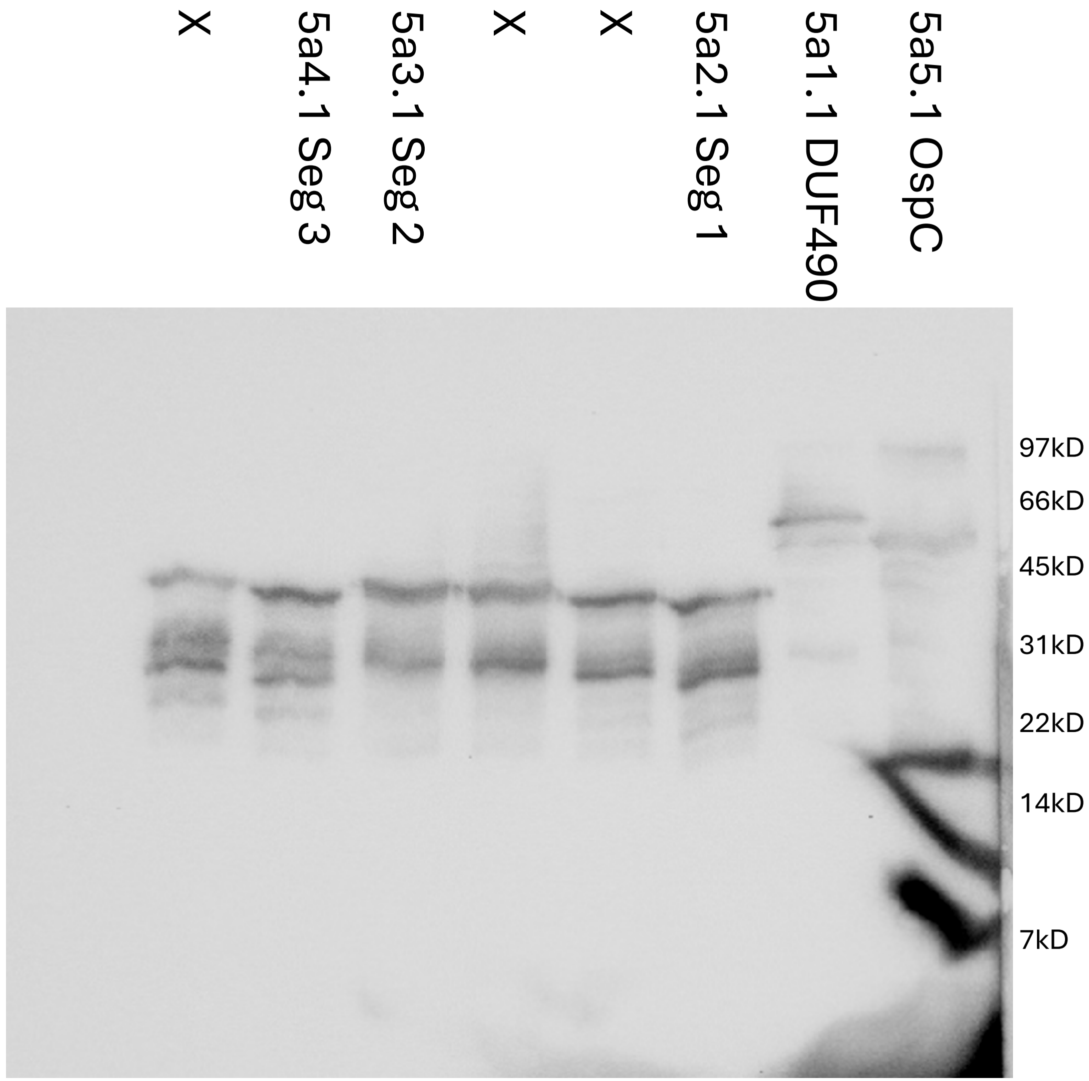

BbBamB WCL anti-His
Figure 5a

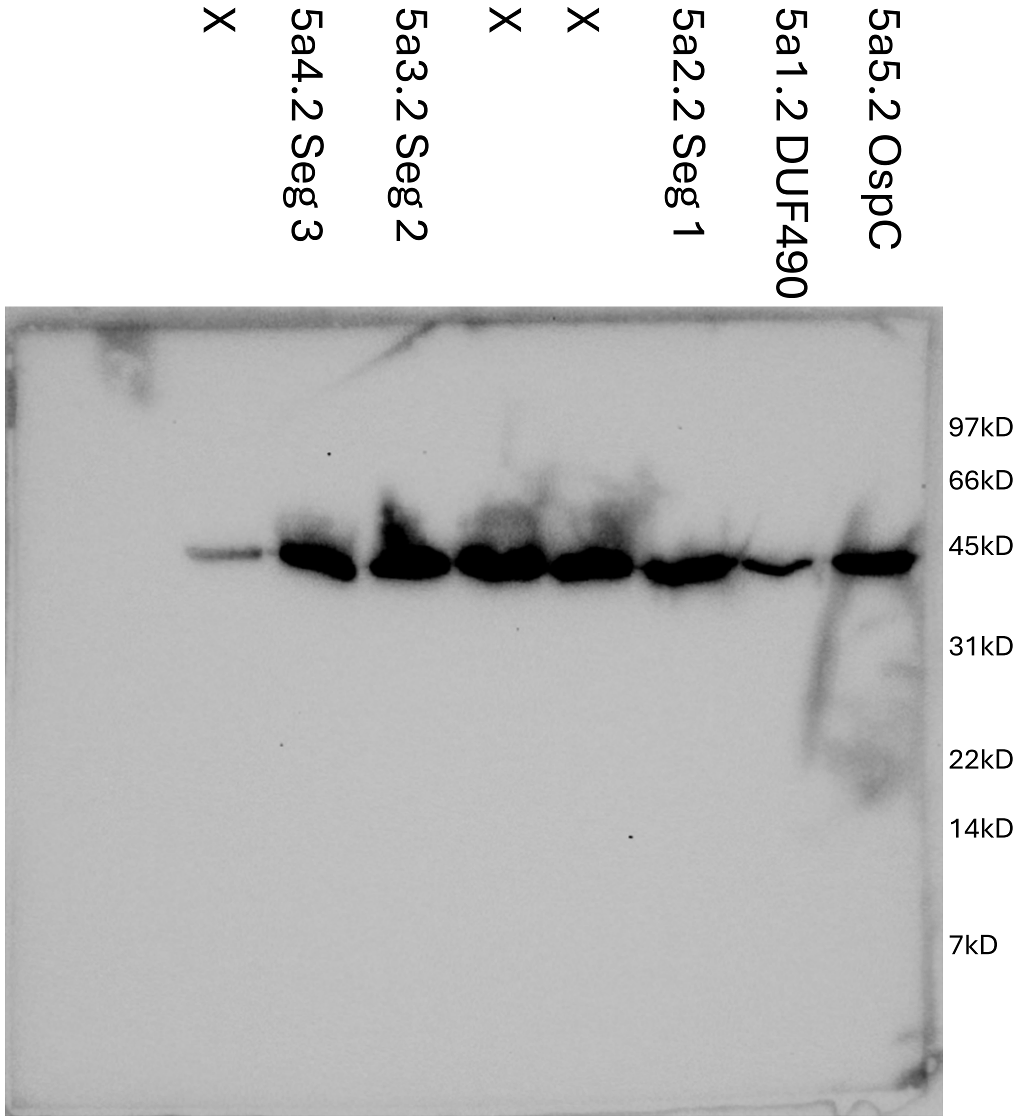

BbBamB Purification anti-GST
Figure 5a

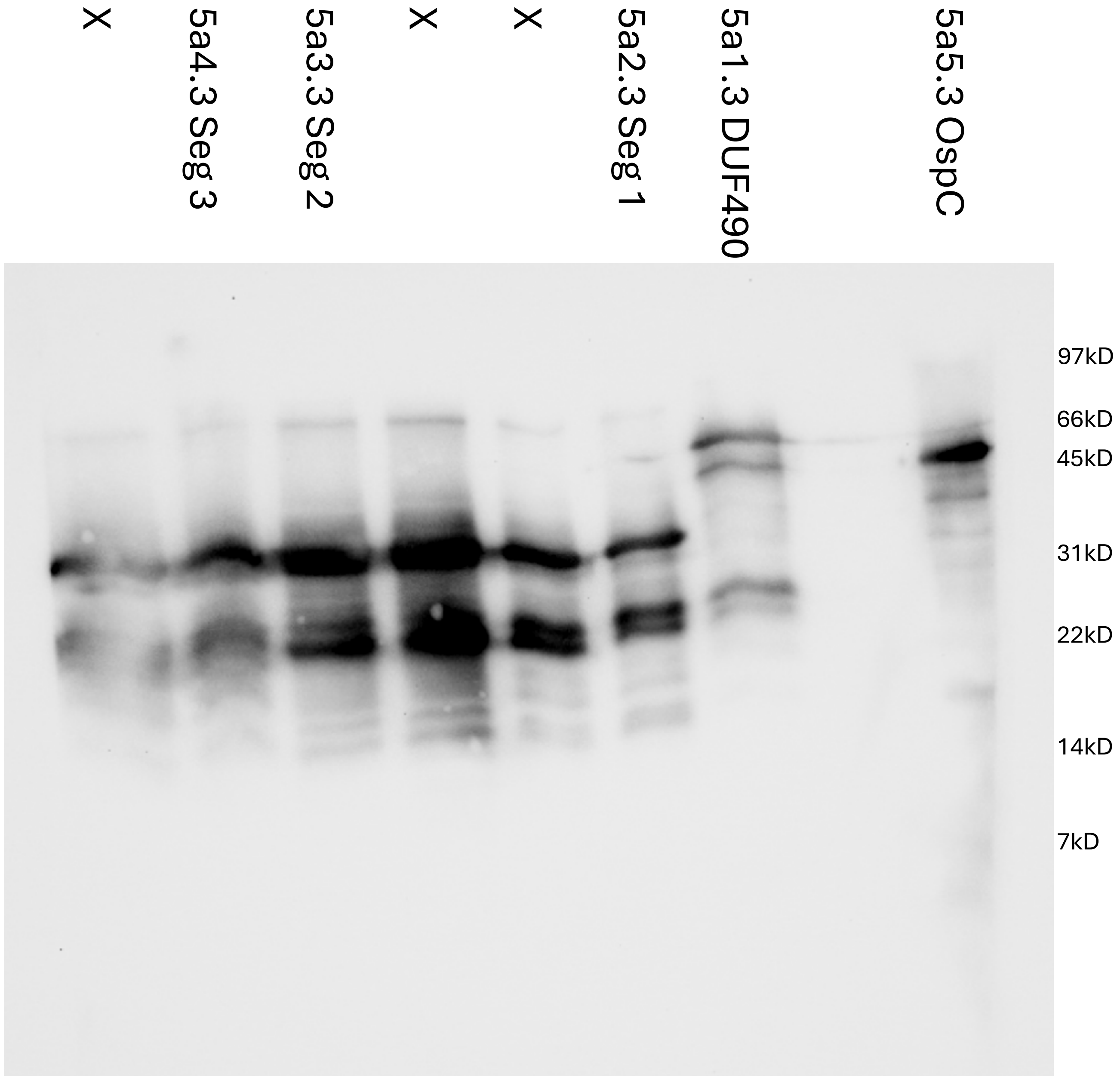

BbBamB Purification anti-His
Figure 5a

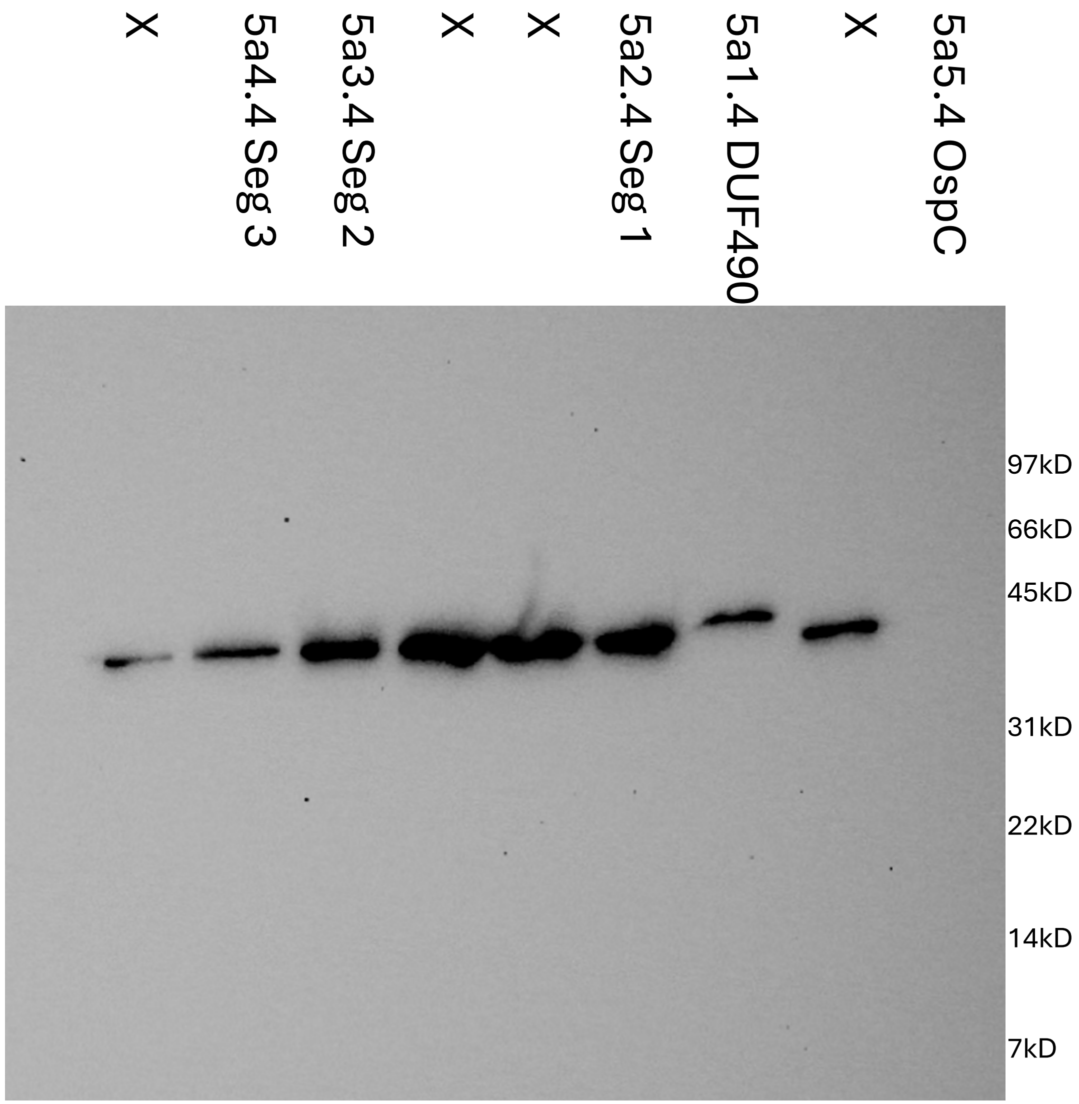

BbBamD WCL anti-GST
Figure 5b

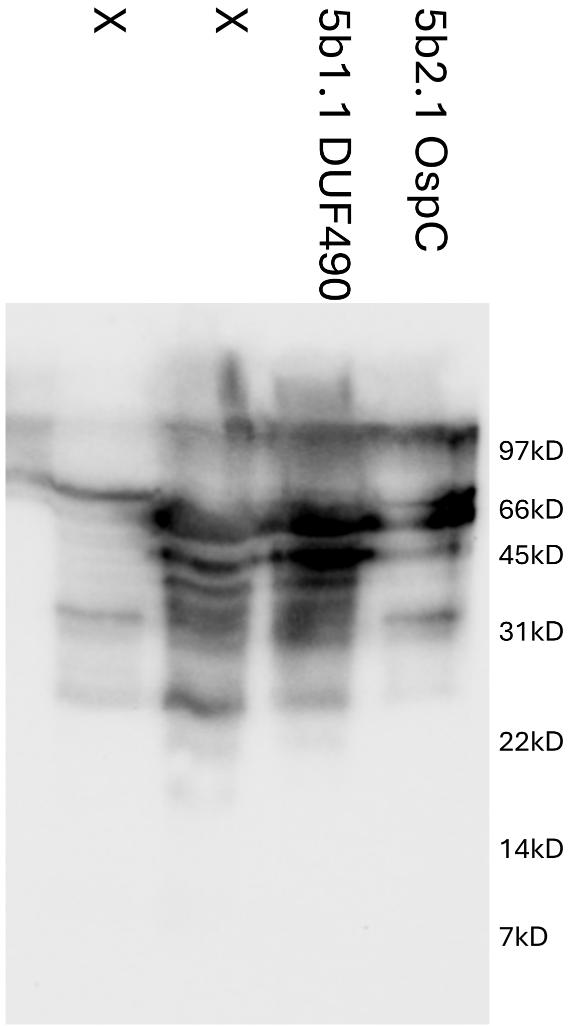

BbBamD WCL anti-His
Figure 5b

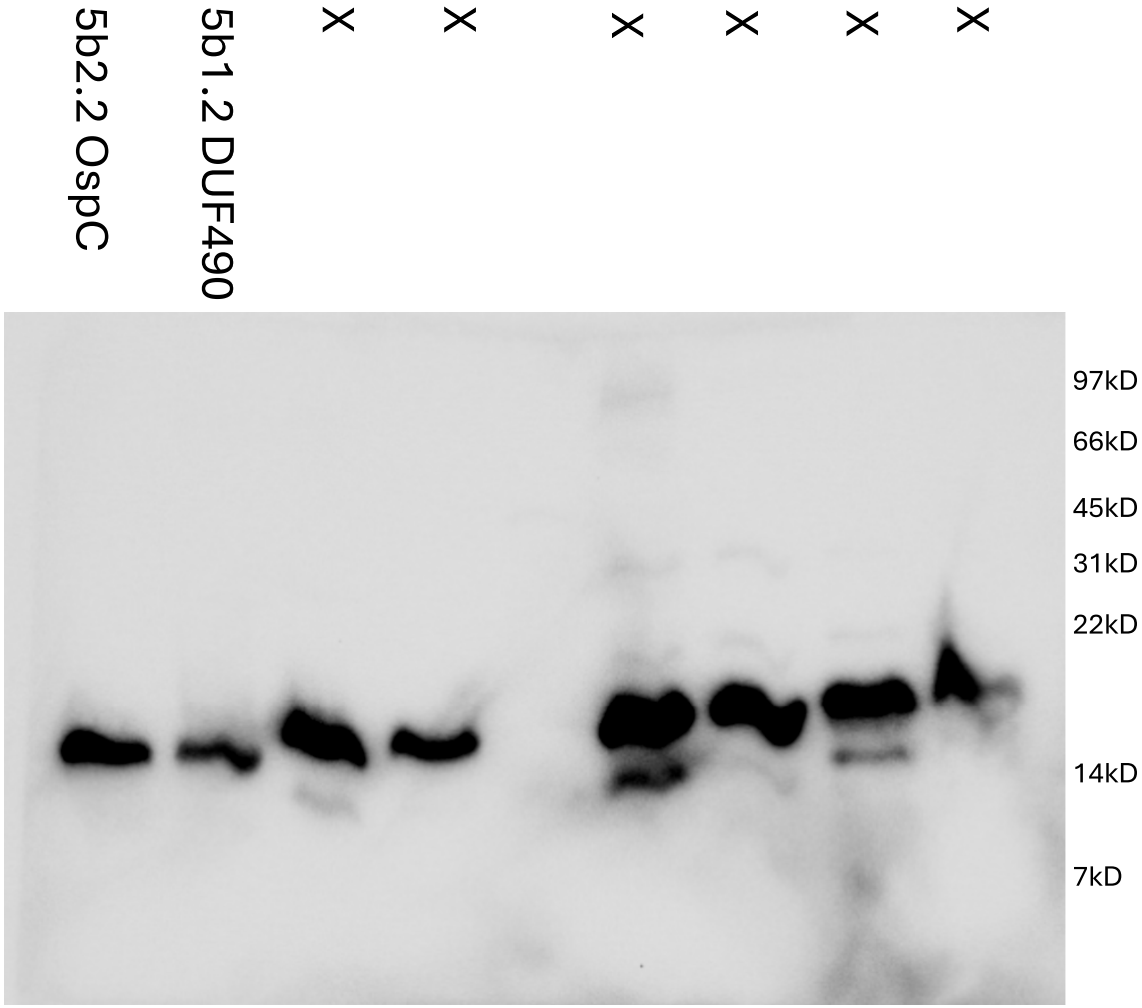

BbBamD Purification anti-GST
Figure 5b

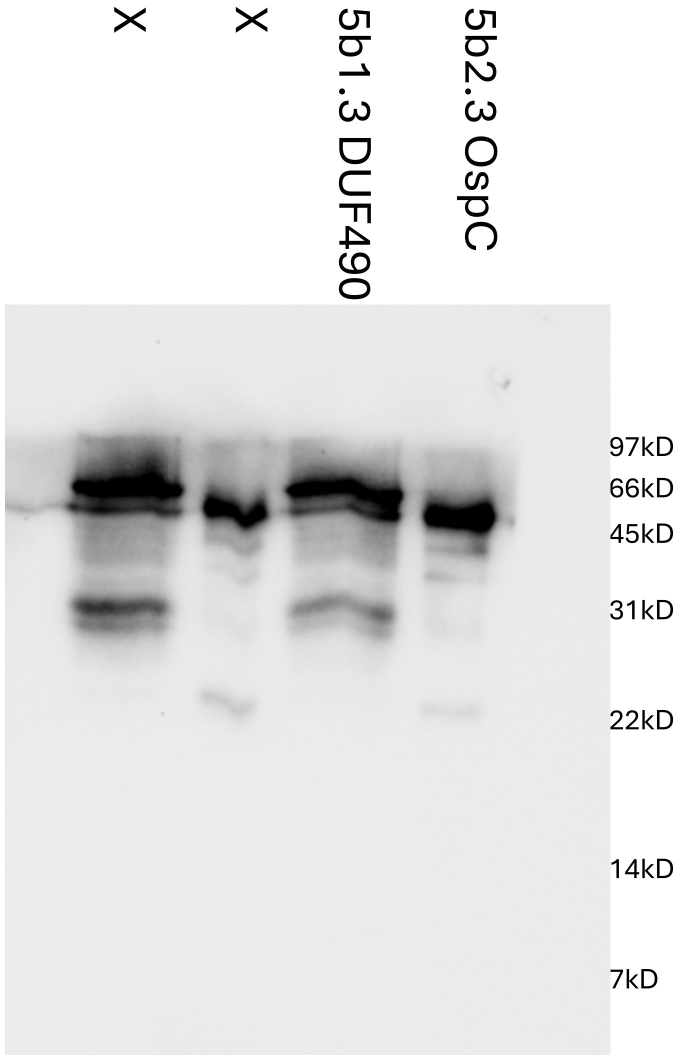

BbBamD Purification anti-His
Figure 5b

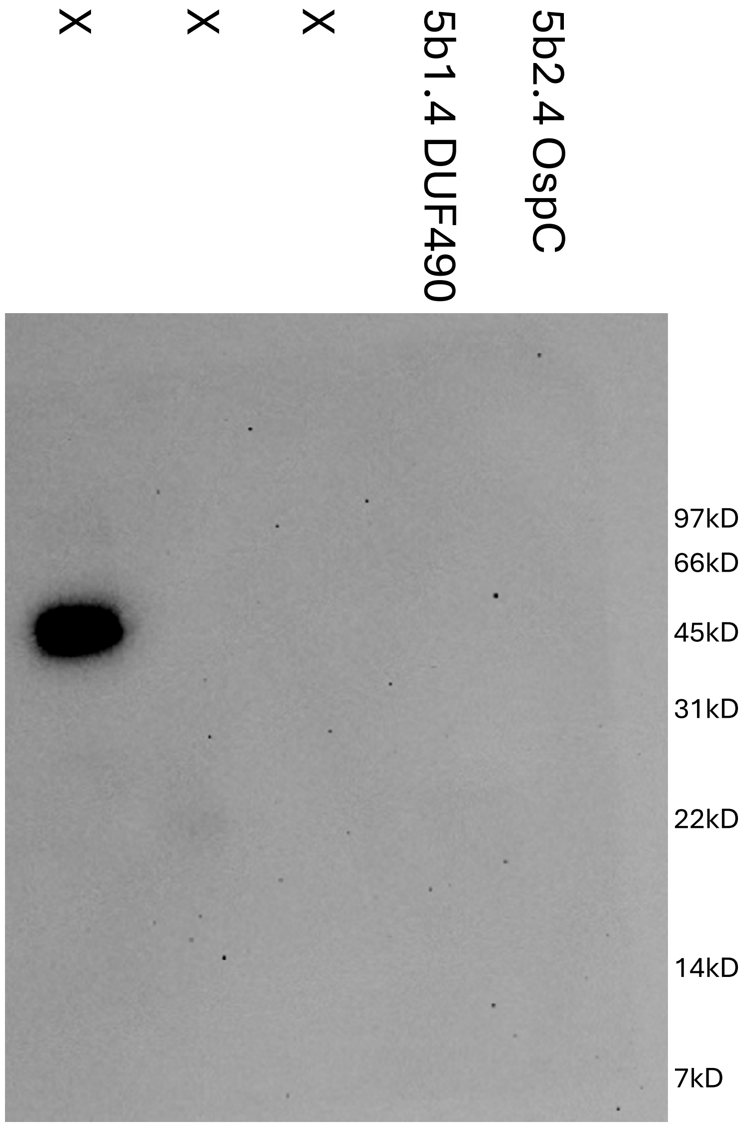

StTamA POTRA1-3 WCL anti-GST
Figure 8a

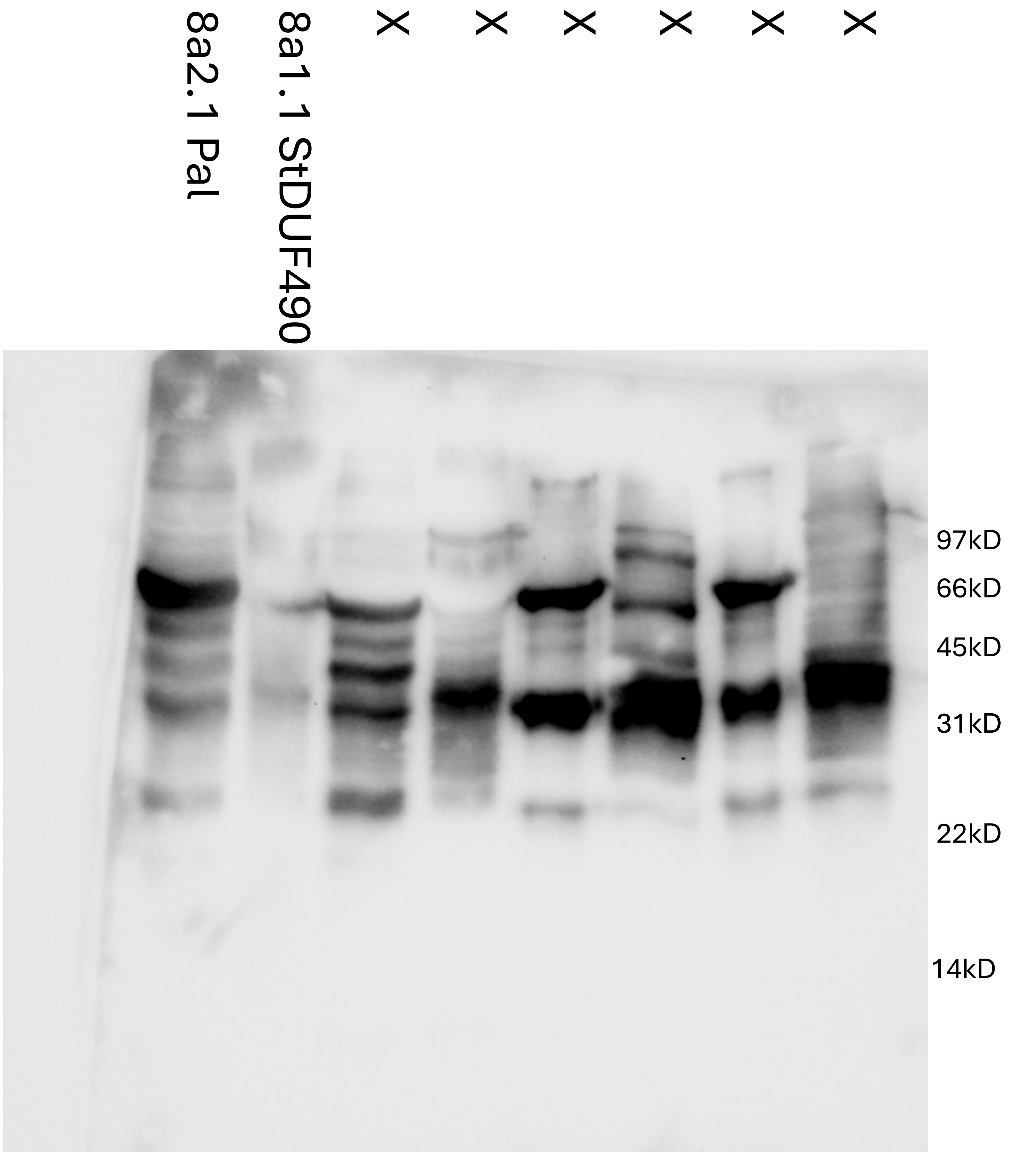

StTamA POTRA1-3 WCL anti-His
Figure 8a

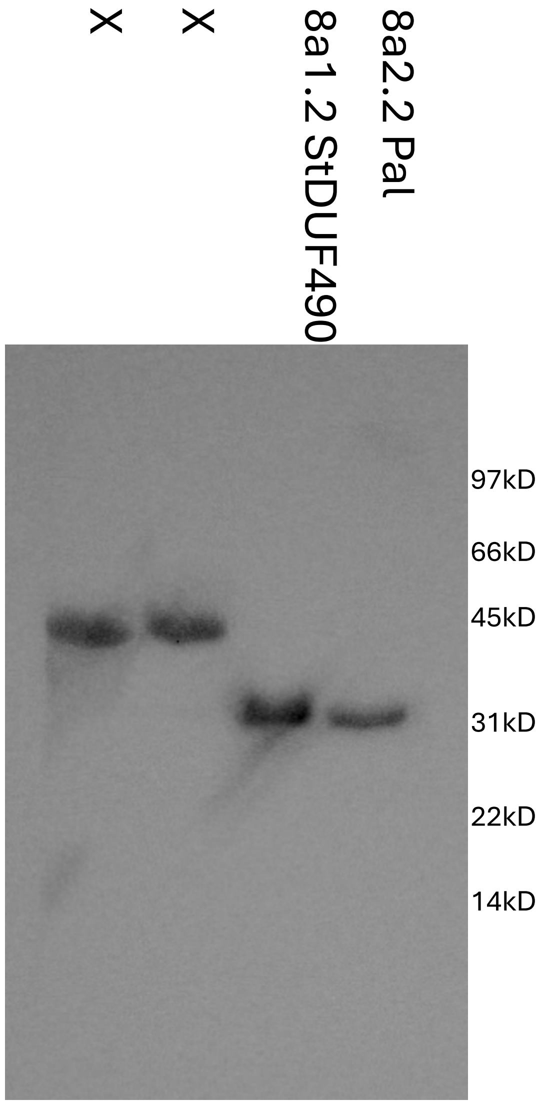

StTamA POTRA1-3 Purification anti-GST
Figure 8a

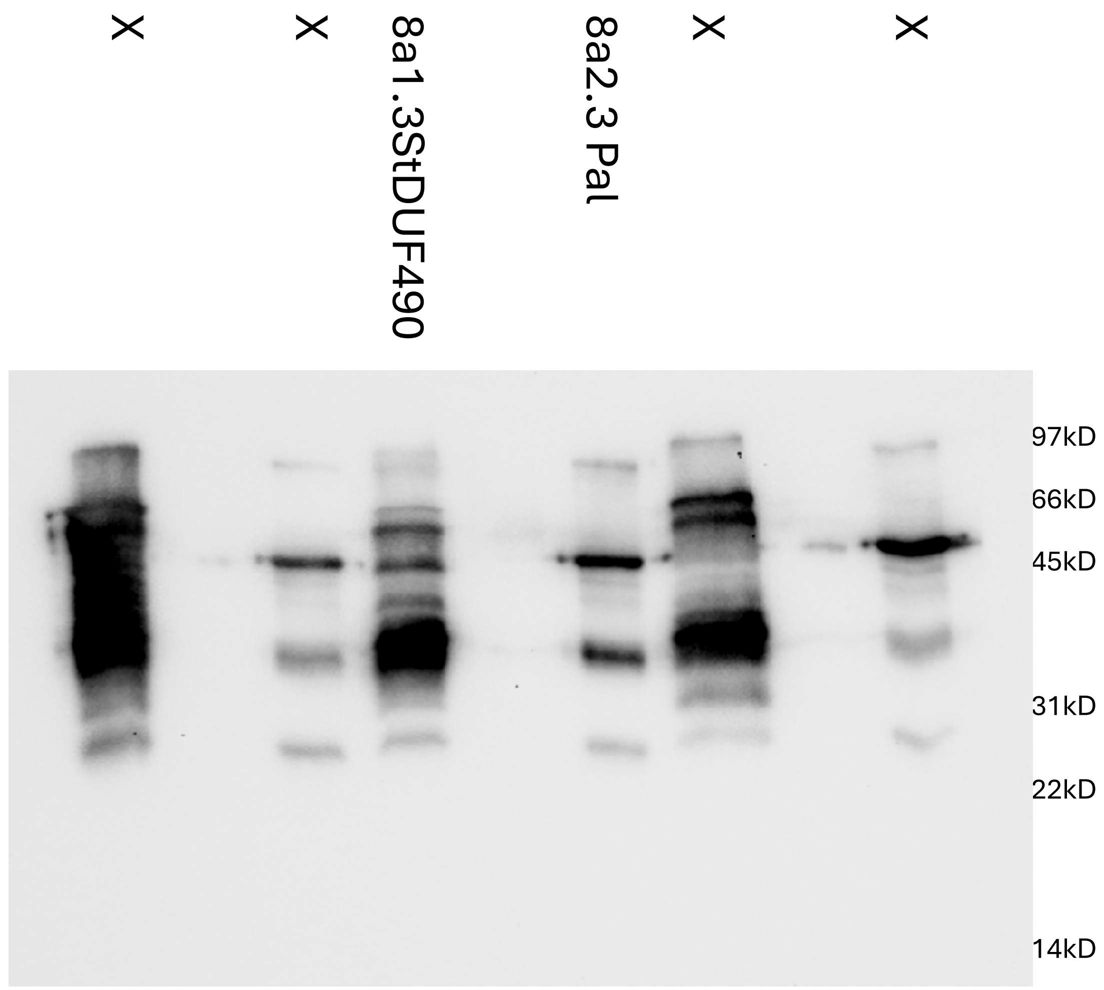

StTamA POTRA1-3 Purification anti-His
Figure 8a

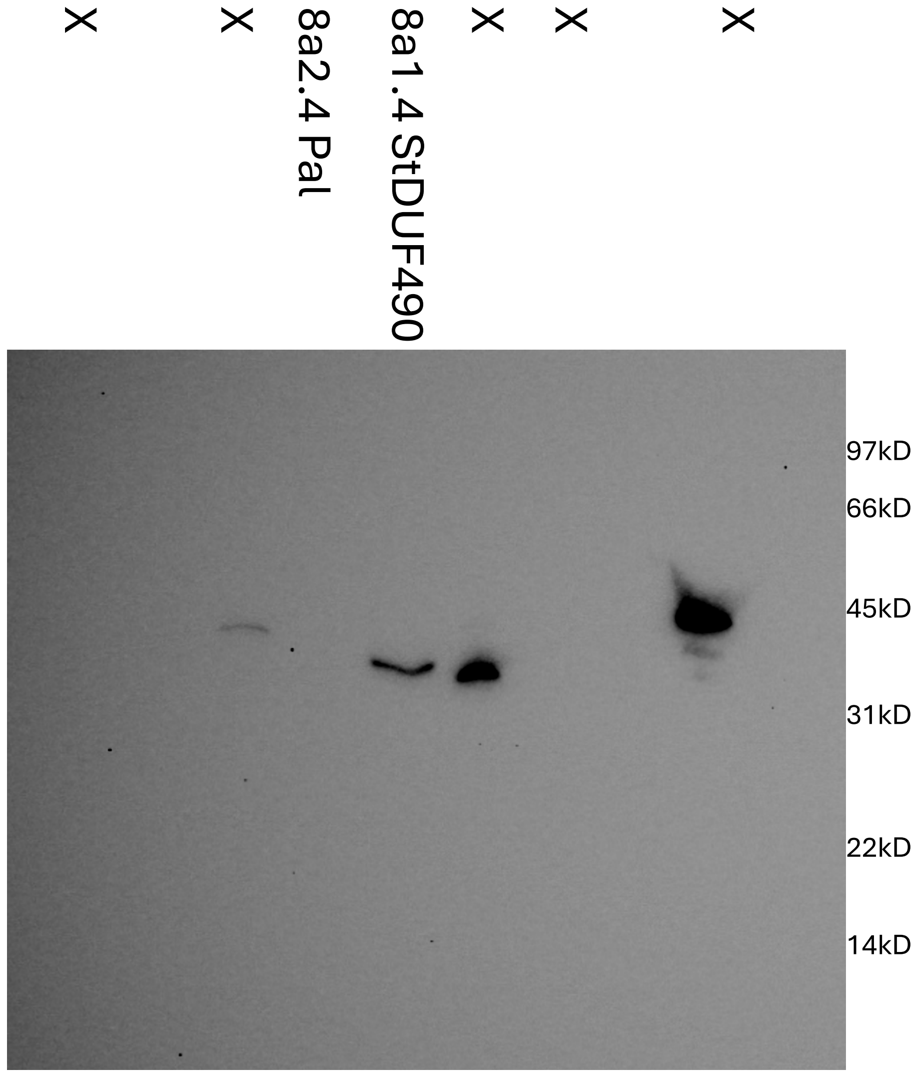

StBamA POTRA1-5 WCL anti-GST
Figure 8b

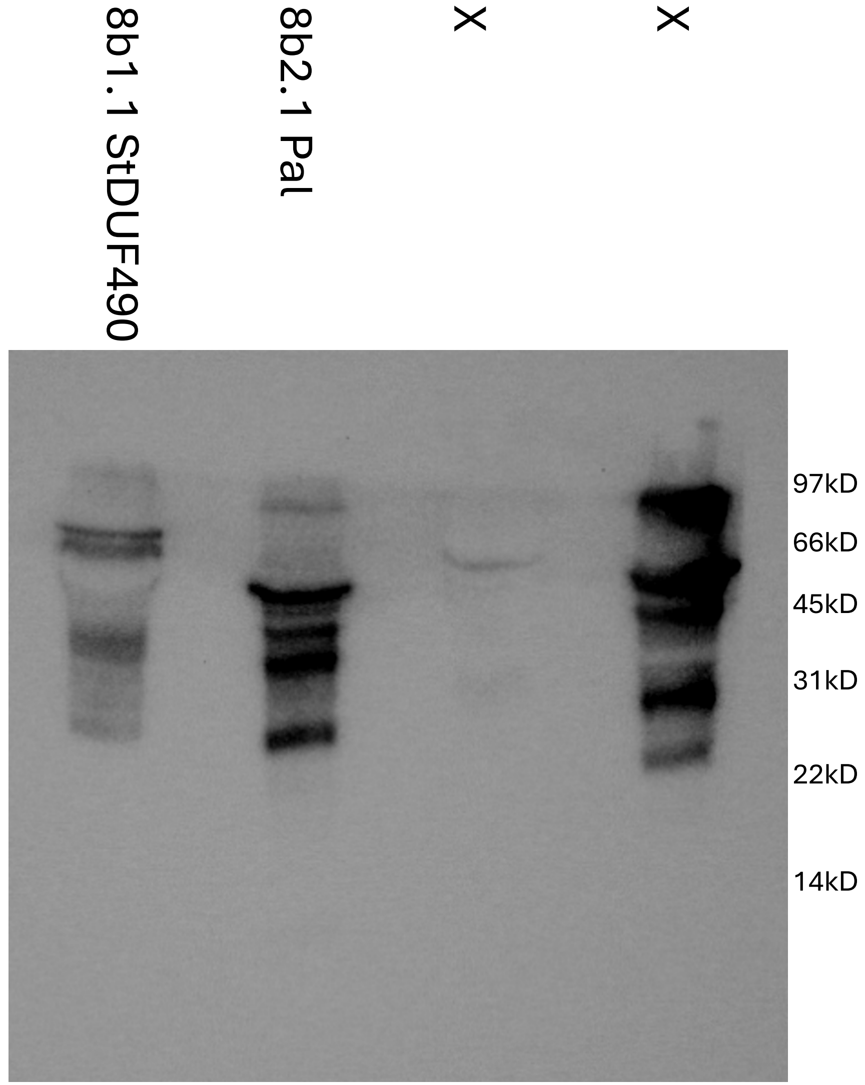

StBamA POTRA1-5 WCL anti-His
Figure 8b

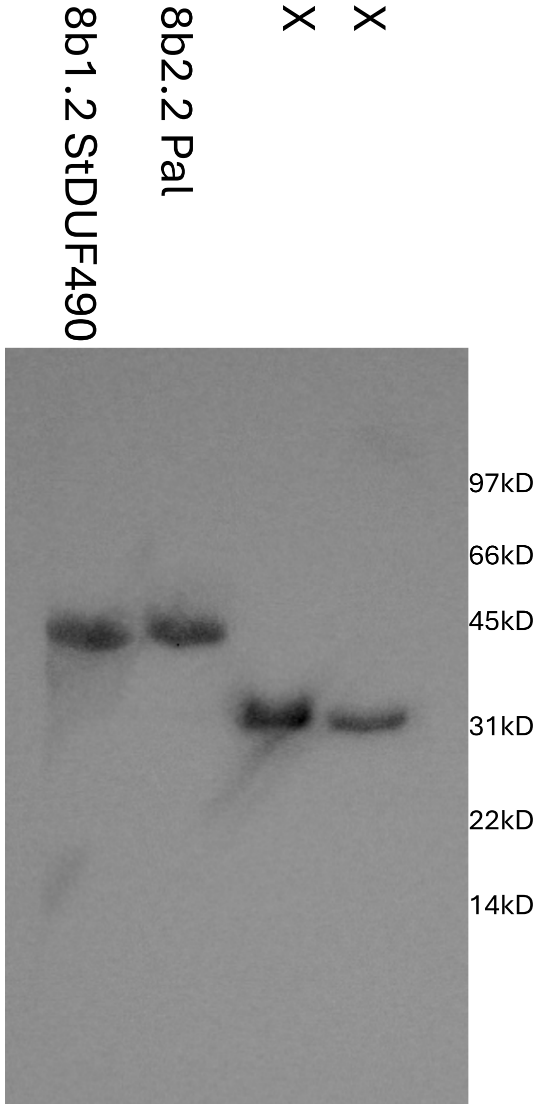

StBamA POTRA1-5 Purification anti-GST
Figure 8b

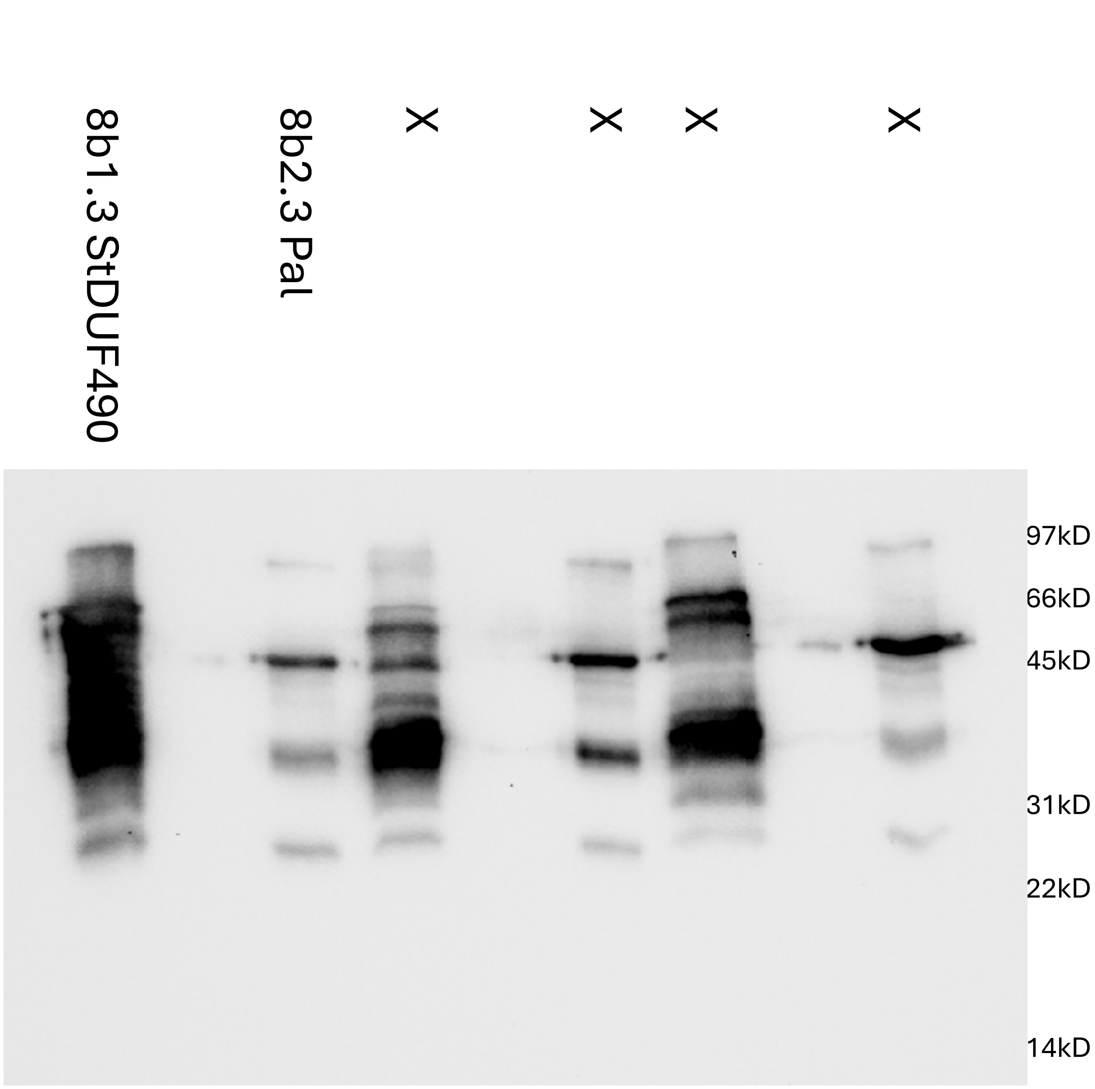

StBamA POTRA1-5 Purification anti-His
Figure 8b

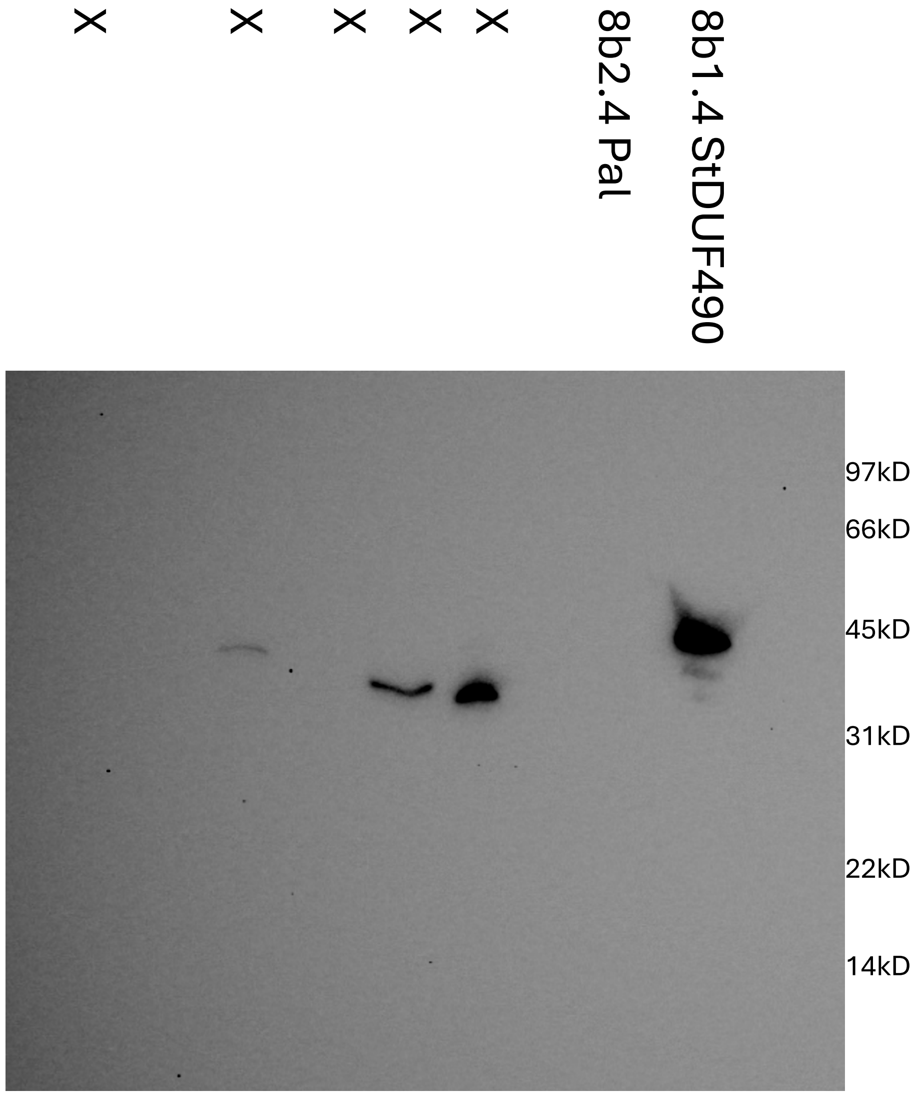

StDUF490 WCL anti-GST
Figure 8c

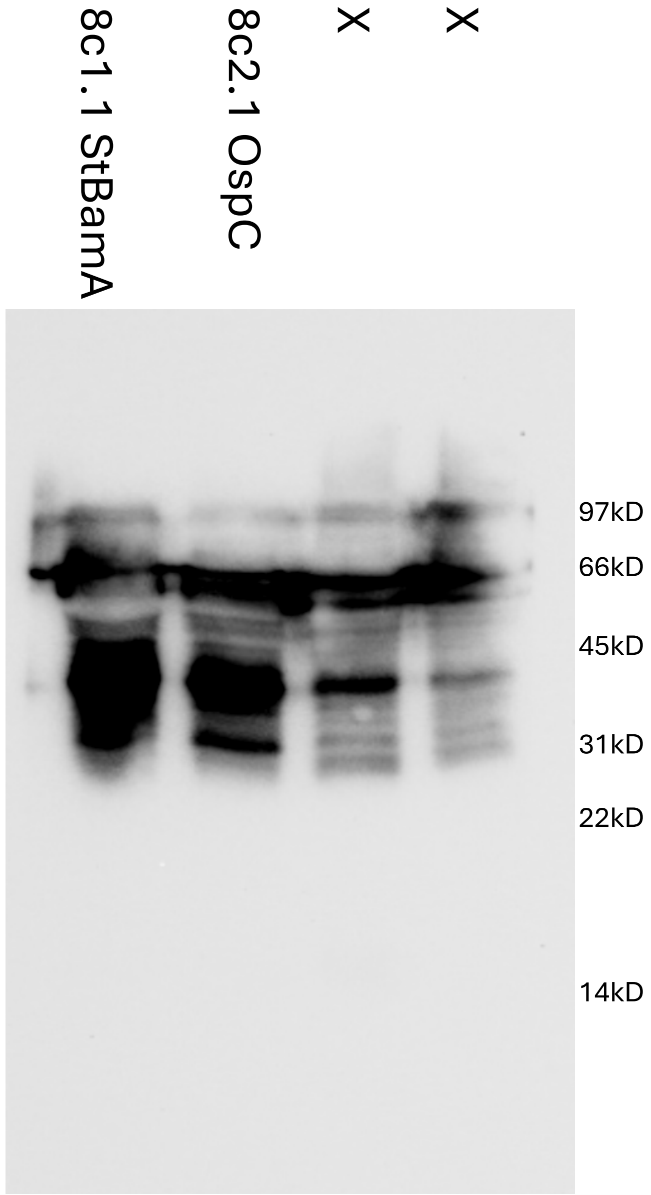

StDUF490 WCL anti-His
Figure 8c

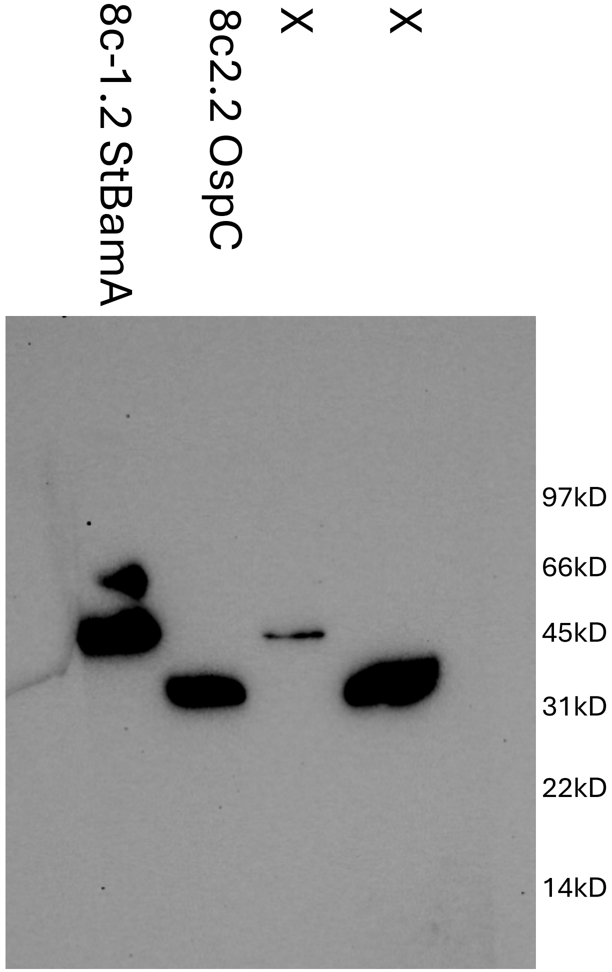

StDUF490 Purification anti-GST
Figure 8c

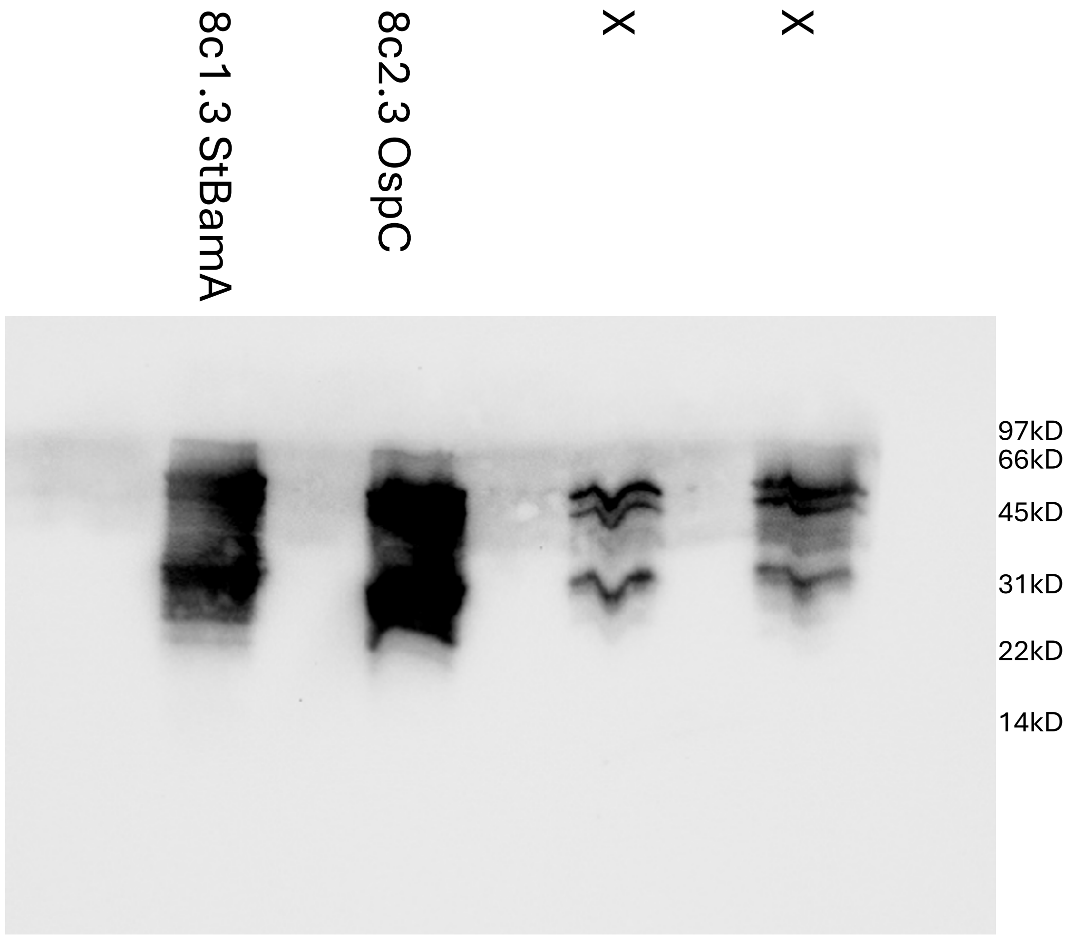

StDUF490 Purification anti-His
Figure 8c

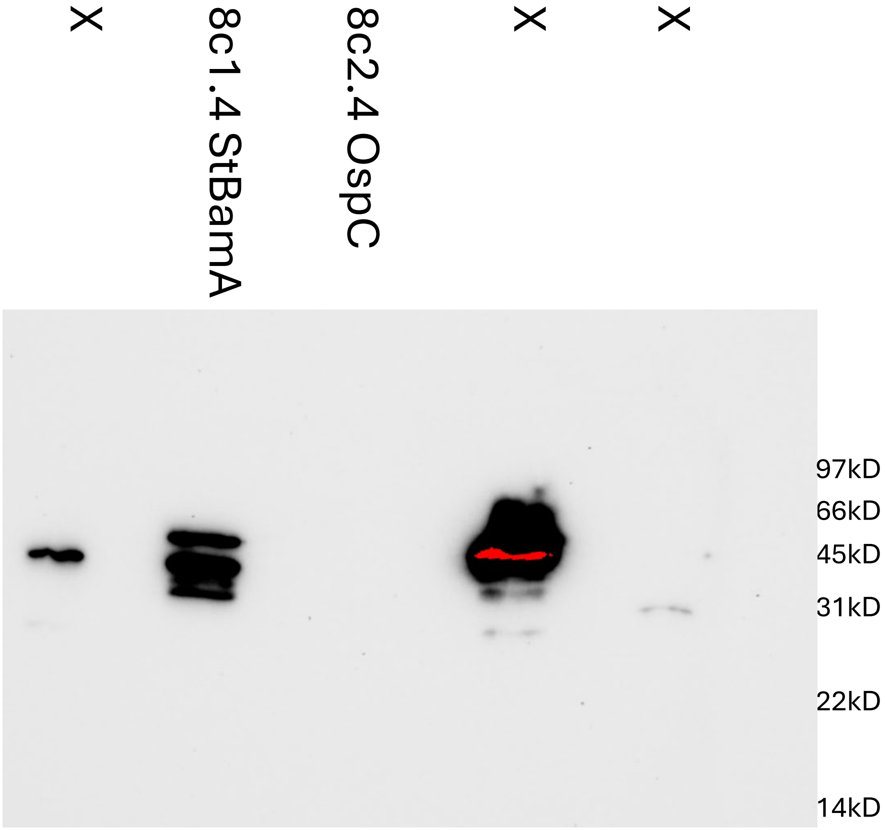

StTamA POTRA1-3 WCL anti-GST
Figure 9a

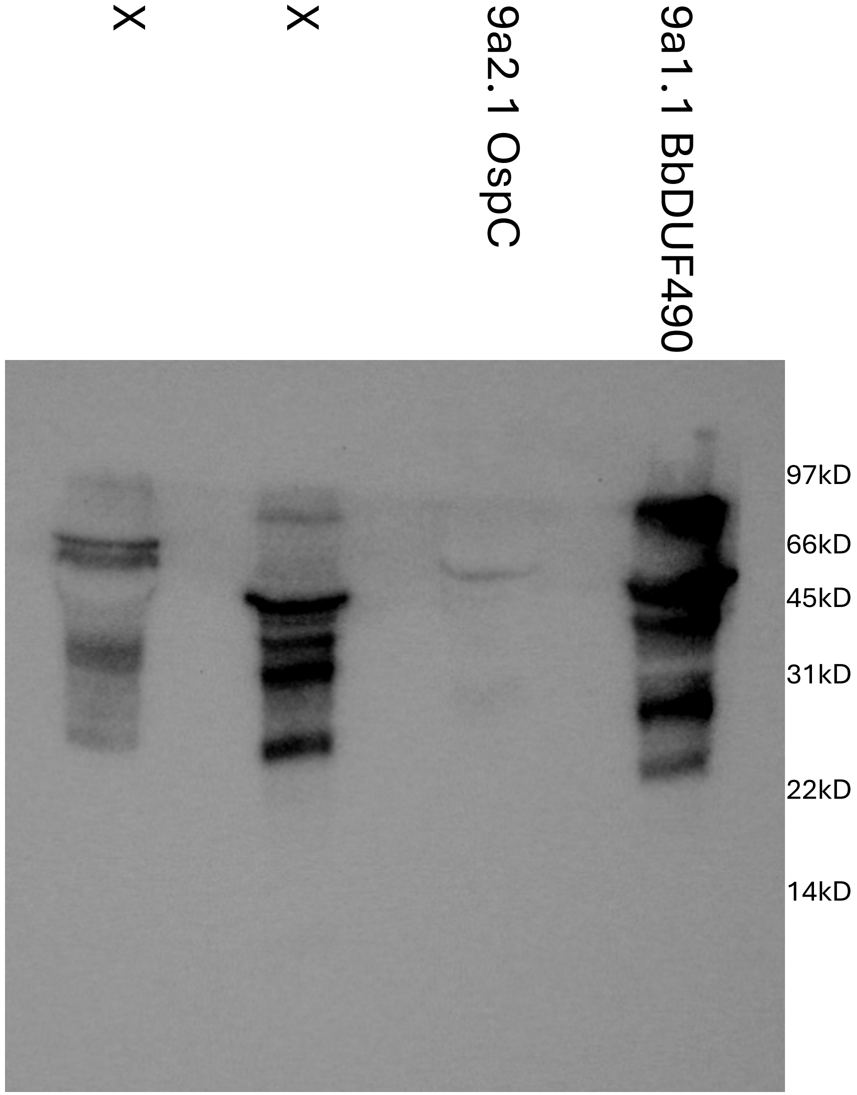

StTamA POTRA1-3 WCL anti-His
Figure 9a

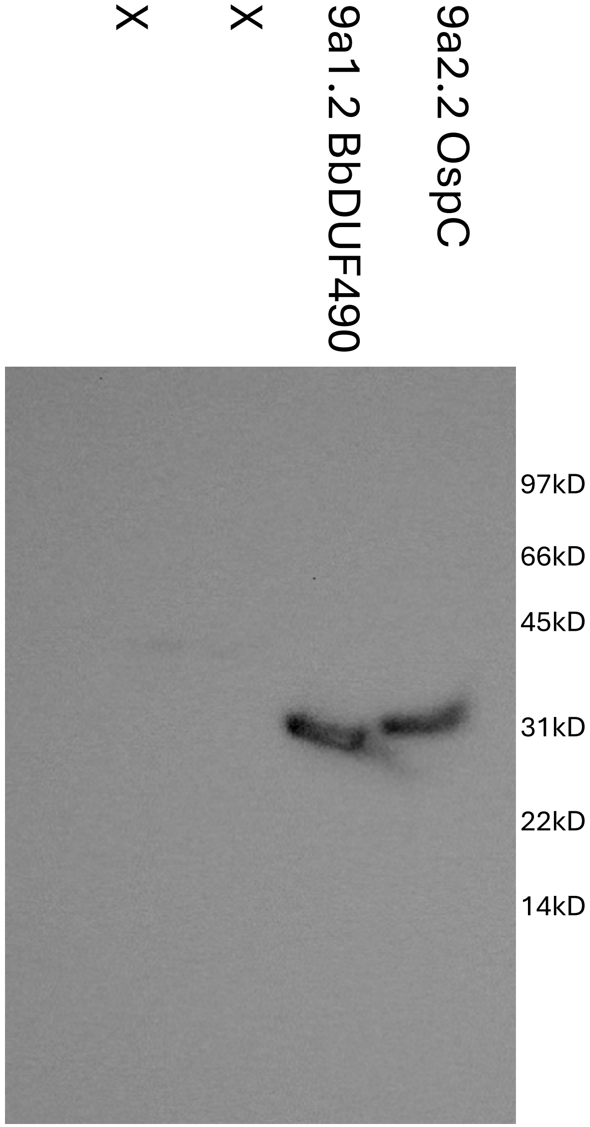

StTamA POTRA1-3 Purification anti-GST
Figure 9a

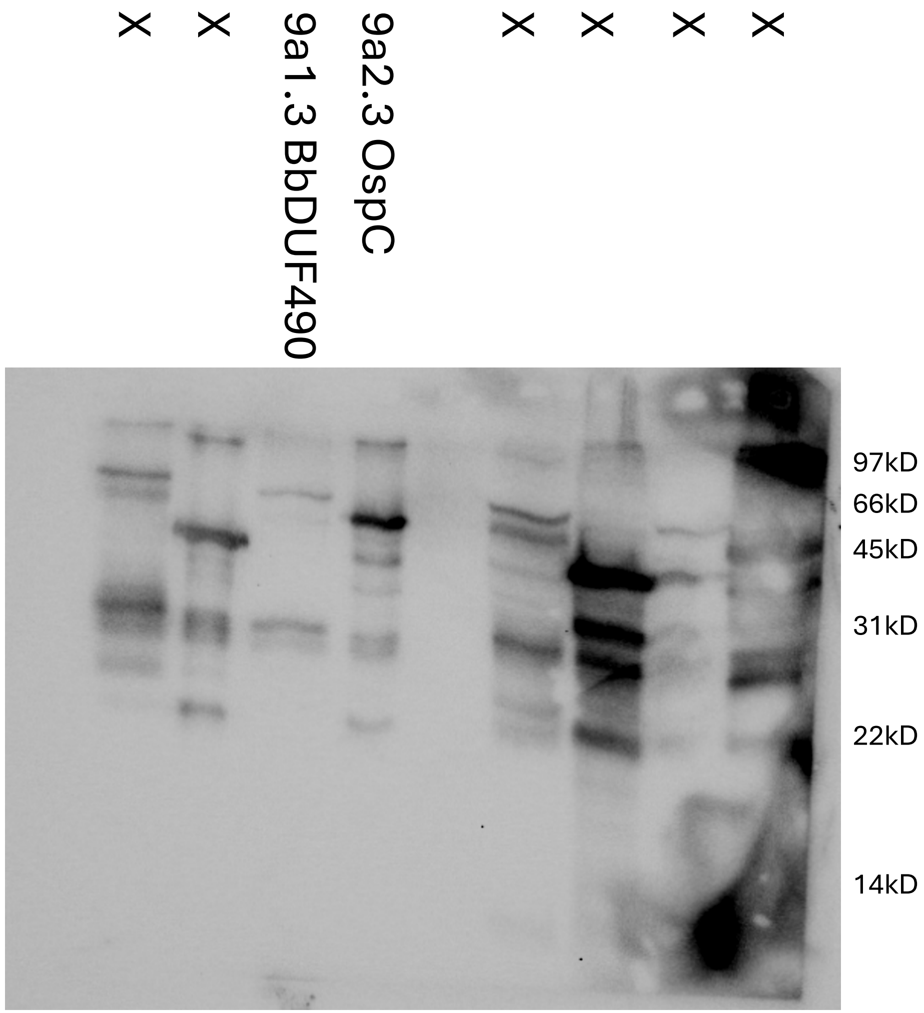

StTamA POTRA1-3 Purification anti-His
Figure 9a

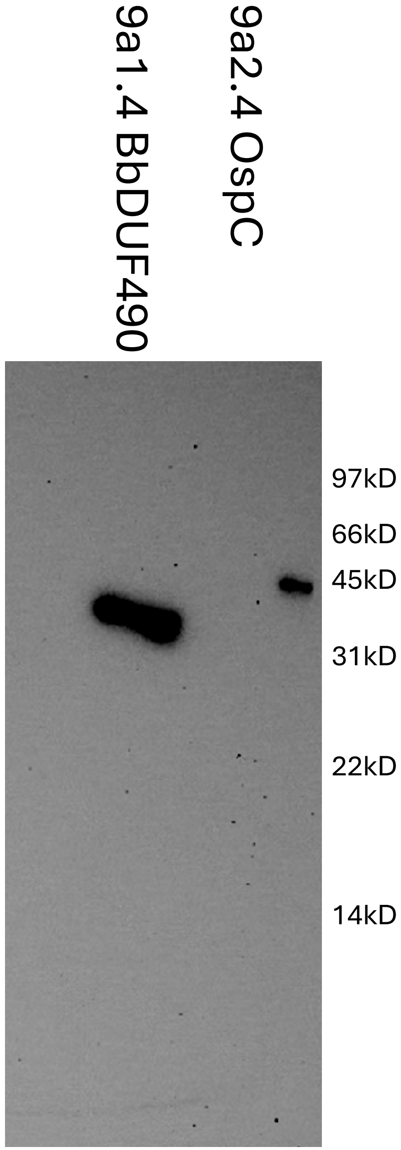

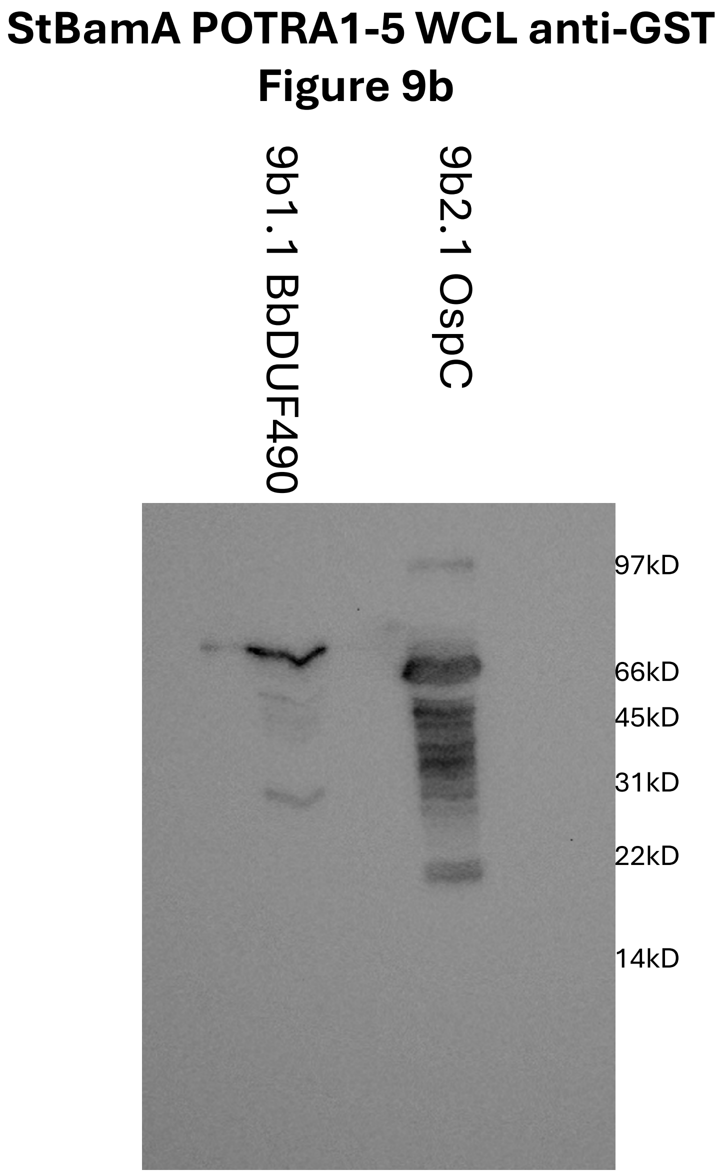
StBamA POTRA1-5 WCL anti-GST
Figure 9b
9b1.1 BbDUF490
9b2.1 OspC
97kD
66kD
45kD
31kD
22kD
14kD

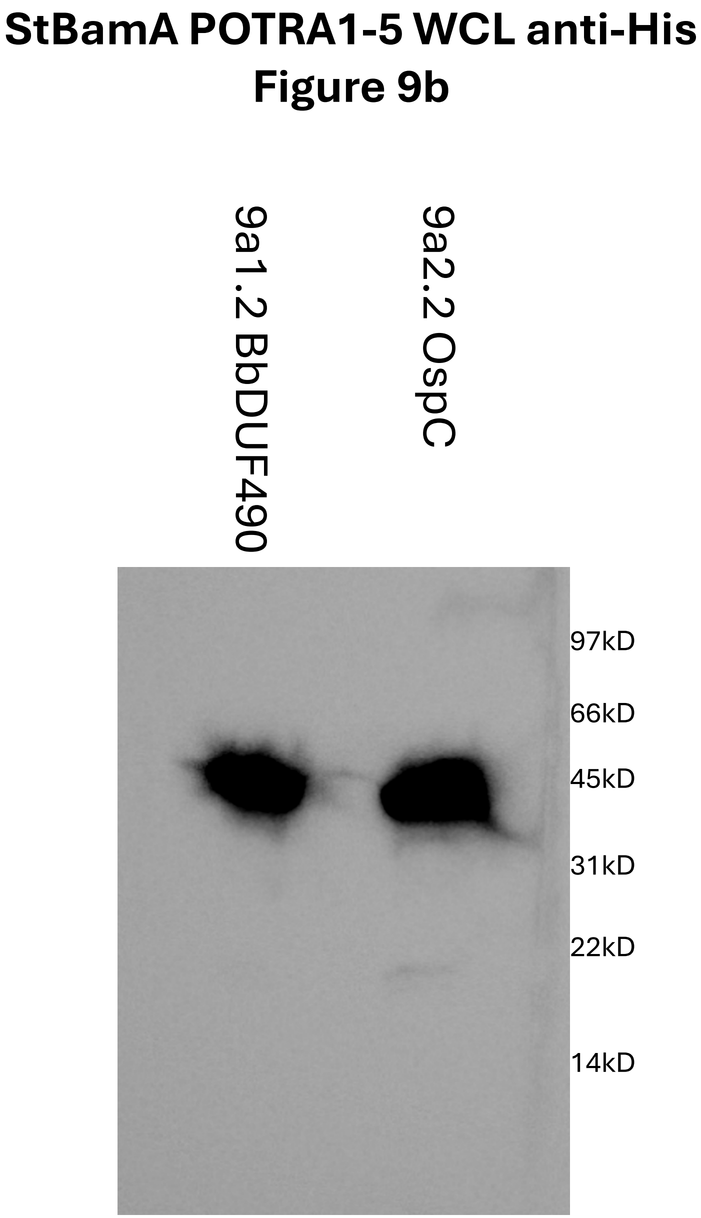
StBamA POTRA1-5 WCL anti-His
Figure 9b
9a1.2 BbDUF490
9a2.2 OspC
97kD
66kD
45kD
31kD
22kD
14kD

StBamA POTRA1-5 Purification anti-GST
Figure 9b

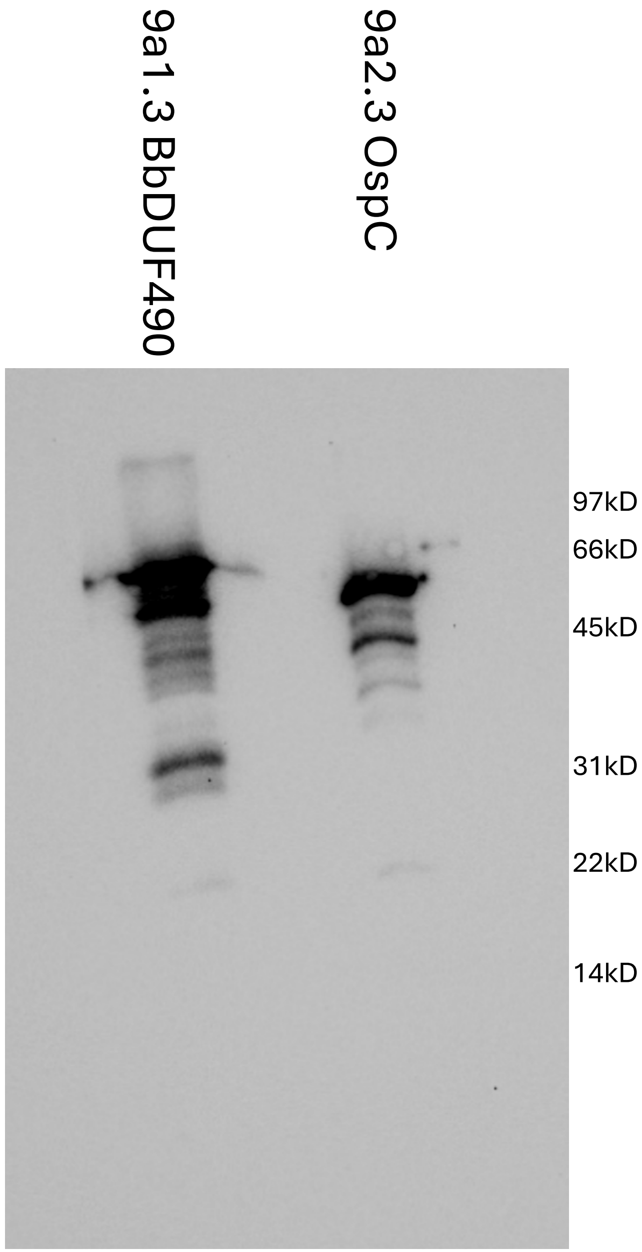

StBamA POTRA1-5 Purification anti-His
Figure 9b

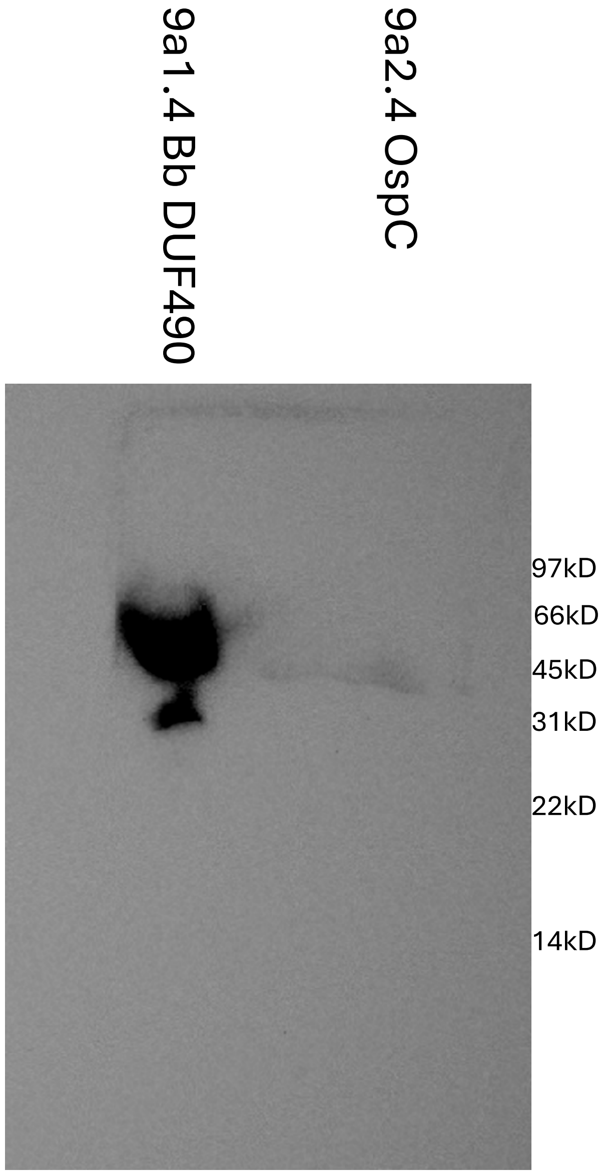

BbBamA POTRA1-5 WCL anti-GST
Figure 9c

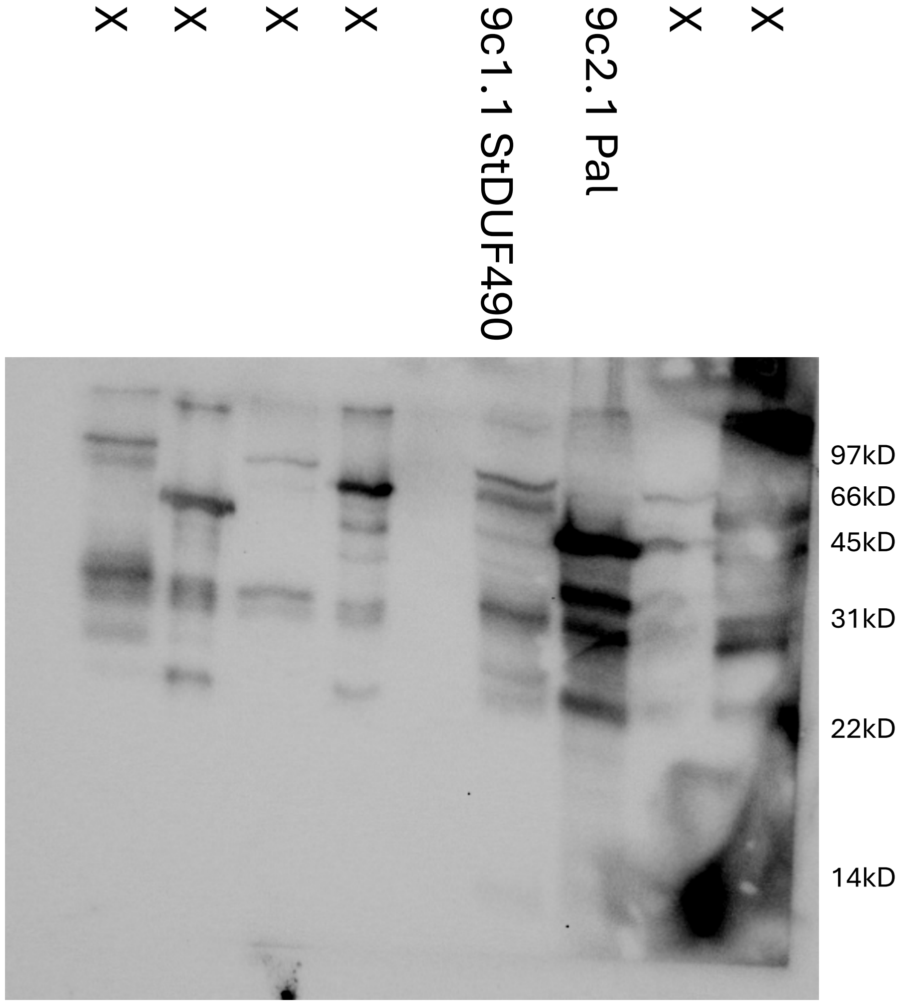

BbBamA POTRA1-5 WCL anti-His
Figure 9c

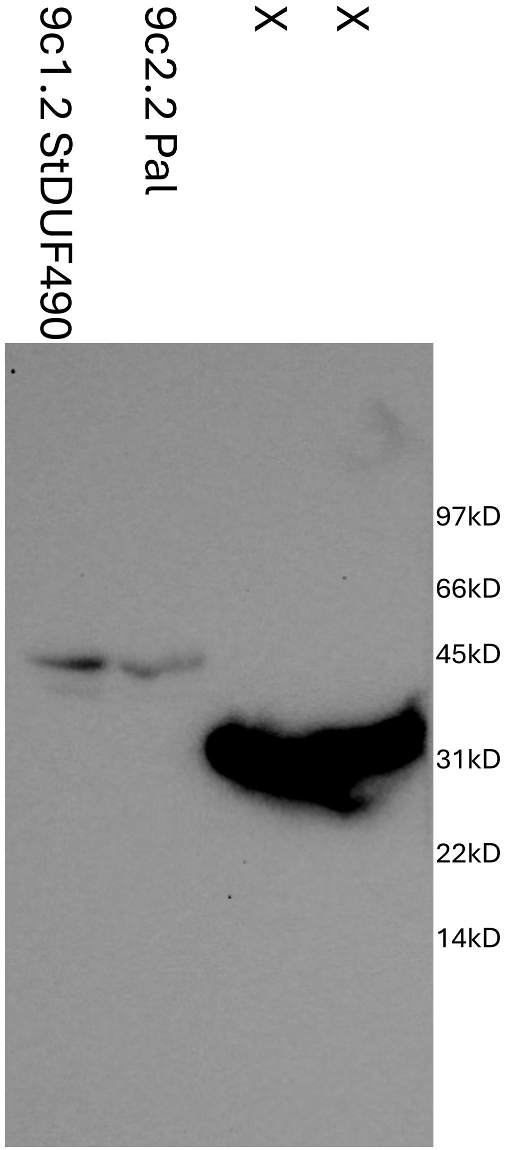

BbBamA POTRA1-5 Purification anti-GST
Figure 9c

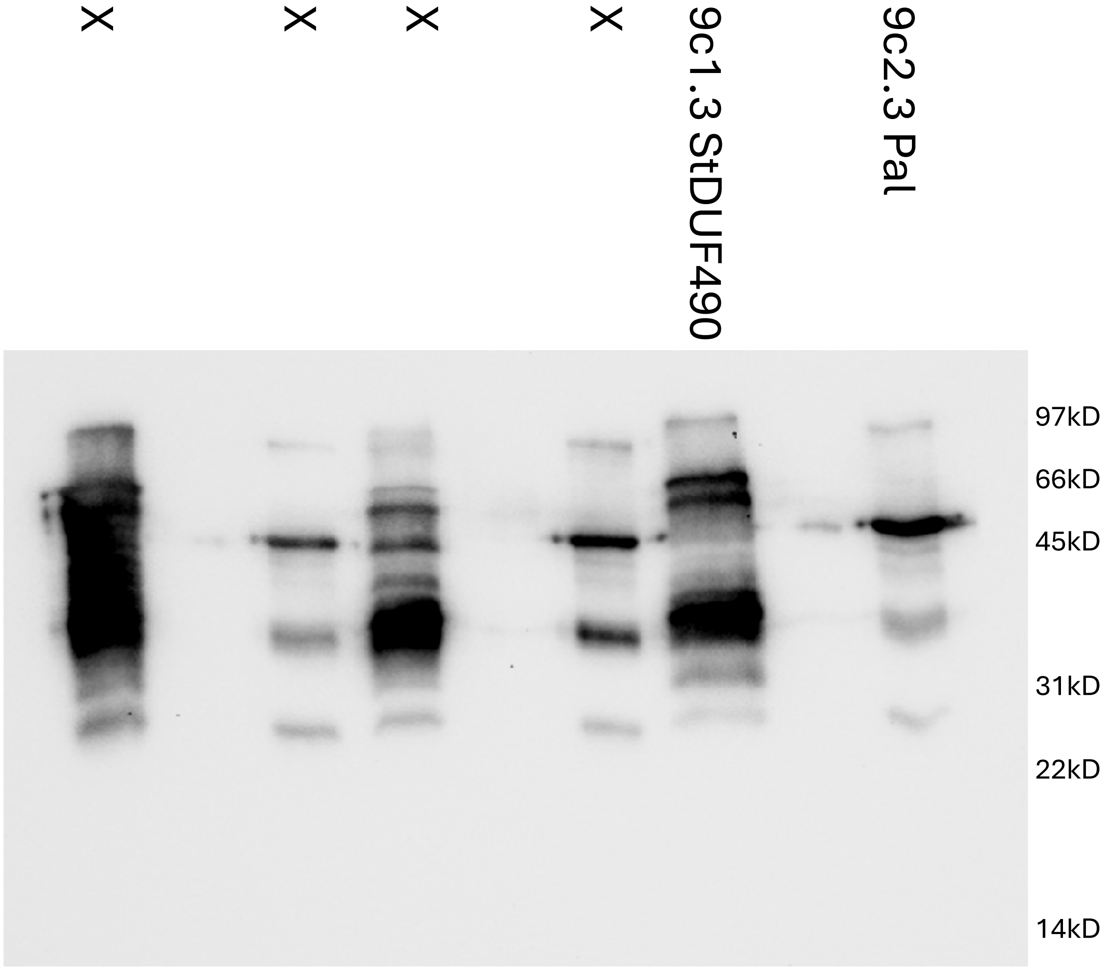

BbBamAPOTRA1-5 Purification anti-His
Figure 9c

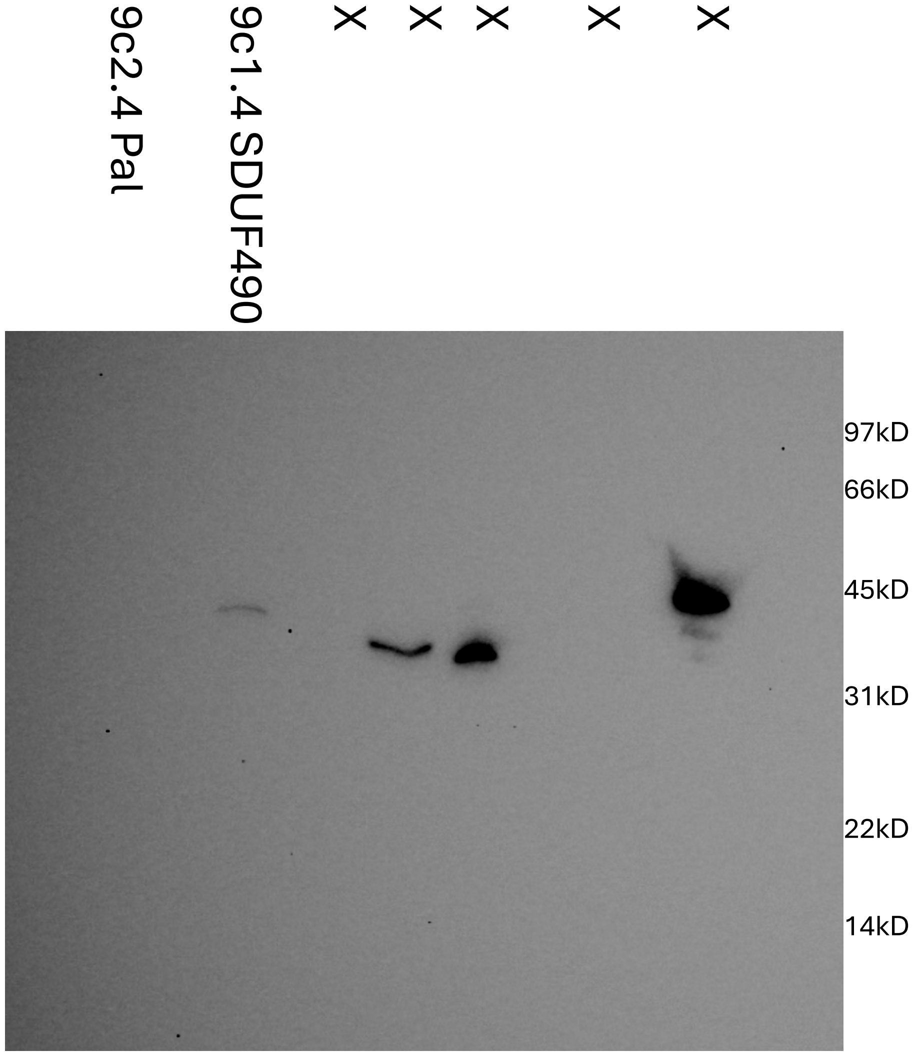

Supplement: S1 File — Labels are as follows: Fig ID. Panel# left to right for Figs 4, 5, 8 and 9. Lane# left to right within panel. Sample Name. (PDF) [file pone.0304839.s002.pdf]
