# Supplementary material for: A conserved C-terminal domain of TamB interacts with multiple BamA POTRA domains in Borreliella burgdorferi
Source: PLoS One. 2024 Aug 29;19(8):e0304839. doi: 10.1371/journal.pone.0304839 (PMC11361582; doi:10.1371/journal.pone.0304839)
Supplement: S2 File — (PDF) [file pone.0304839.s003.pdf]

Threading the sequence onto the templates:

```
partial_thread.macosclangrelease -in:file:fasta Bb.fasta -in:file:alignment EcTamATamB.grishin -in:file:template_pdb EcTamATamB.template.pdb

partial_thread.macosclangrelease -in:file:fasta Bb.fasta -in:file:alignment BbBamA.grishin -in:file:template_pdb BbBamA.template.pdb

partial_thread.macosclangrelease -in:file:fasta Bb.fasta -in:file:alignment BbTamB.grishin -in:file:template_pdb BbTamB.template.pdb
```

Building fragment libraries

```
Rosetta/tools/fragment_tools/make_fragments.pl -verbose Bb.fasta -frag_sizes 3,9 -n_frags 200
```

Generating models

```
rosetta_scripts.linuxgccrelease @flags
```

Relaxing models

```
relax.linuxgccrelease @relax.flags
```
