# Supplementary material for: A conserved C-terminal domain of TamB interacts with multiple BamA POTRA domains in Borreliella burgdorferi
Source: PLoS One. 2024 Aug 29;19(8):e0304839. doi: 10.1371/journal.pone.0304839 (PMC11361582; doi:10.1371/journal.pone.0304839)
Supplement: S3 File — Available in repository at https://simtk.org/projects/bama_tamb_bb. (DOCX) [file pone.0304839.s004.docx]

**Files used by RosettaCMs****.** Available in repository at <https://simtk.org/projects/bama_tamb_bb>
